# Core Outcome Set–STAndards for Reporting: The COS-STAR Statement

**DOI:** 10.1371/journal.pmed.1002148

**Published:** 2016-10-18

**Authors:** Jamie J. Kirkham, Sarah Gorst, Douglas G. Altman, Jane M. Blazeby, Mike Clarke, Declan Devane, Elizabeth Gargon, David Moher, Jochen Schmitt, Peter Tugwell, Sean Tunis, Paula R. Williamson

**Affiliations:** 1 MRC North West Hub for Trials Methodology Research, Department of Biostatistics, University of Liverpool, Liverpool, United Kingdom; 2 Centre for Statistics in Medicine, Nuffield Department of Orthopaedics, Rheumatology and Musculoskeletal Sciences, University of Oxford, United Kingdom; 3 MRC ConDuCT II Hub for Trials Methodology Research, School of Social and Community Medicine, University of Bristol, Bristol, United Kingdom; 4 Northern Ireland Hub for Trials Methodology Research, Centre for Public Health, Queen's University Belfast, Belfast, United Kingdom; 5 National University of Ireland Galway and HRB Trials Methodology Research Network, Ireland; 6 Ottawa Methods Centre, Ottawa Hospital Research Institute, Ottawa, Canada; 7 Center for Evidence-Based Healthcare, Medizinische Fakultät, Technische Universität Dresden, Dresden, Germany; 8 Institute of Population Health, University of Ottawa, Ottawa, Canada; 9 Center for Medical Technology Policy, Baltimore, Maryland, United States of America

## Abstract

**Background:**

Core outcome sets (COS) can enhance the relevance of research by ensuring that outcomes of importance to health service users and other people making choices about health care in a particular topic area are measured routinely. Over 200 COS to date have been developed, but the clarity of these reports is suboptimal. COS studies will not achieve their goal if reports of COS are not complete and transparent.

**Methods and Findings:**

In recognition of these issues, an international group that included experienced COS developers, methodologists, journal editors, potential users of COS (clinical trialists, systematic reviewers, and clinical guideline developers), and patient representatives developed the Core Outcome Set–STAndards for Reporting (COS-STAR) Statement as a reporting guideline for COS studies. The developmental process consisted of an initial reporting item generation stage and a two-round Delphi survey involving nearly 200 participants representing key stakeholder groups, followed by a consensus meeting. The COS-STAR Statement consists of a checklist of 18 items considered essential for transparent and complete reporting in all COS studies. The checklist items focus on the introduction, methods, results, and discussion section of a manuscript describing the development of a particular COS. A limitation of the COS-STAR Statement is that it was developed without representative views of low- and middle-income countries. COS have equal relevance to studies conducted in these areas, and, subsequently, this guideline may need to evolve over time to encompass any additional challenges from developing COS in these areas.

**Conclusions:**

With many ongoing COS studies underway, the COS-STAR Statement should be a helpful resource to improve the reporting of COS studies for the benefit of all COS users.

## Abstract 2

Paula Williamson and colleagues present reporting standards for studies that report core outcome sets for health research.

## Introduction

There is growing recognition that insufficient attention has been paid to the outcomes measured in clinical trials, which need to be relevant to health service users and other people making choices about health care if the findings of research are to influence practice and future research. In addition, outcome reporting bias, whereby results are selected for inclusion in a trial report on the basis of those results, has been identified as a problem for the interpretation of published data.

The development and implementation of core outcome sets (COS) is drawing increasing attention across all areas of research in health [1] and has relevance for research practice on a global scale. A recent survey reveals that some trialists, systematic reviewers, and guideline developers (COS users) do now refer to COS studies as a starting point for outcome selection in their work [1]. The use of COS can help improve the consistency in outcome measurement and reduce outcome reporting bias, which has led to much unnecessary waste in the production and reporting of research [2].

A recently updated systematic review identified over 200 published COS studies [1], and many more are known to be under development [3]. The first step in COS development is typically “what to measure,” whereas the “how” and “when” usually come later. The value of a COS depends largely on why and how it was developed. The credibility of the group that has developed the COS, in relation to their experience of outcome assessment and how they have engaged with key stakeholders during the development process, may influence subsequent uptake of the COS. Furthermore, the reporting quality of COS development studies is also relevant to implementation. Recent work shows that reporting quality is currently variable [4], restricting the ability of potential users of COS, for example clinical trialists, systematic reviewers, and guideline developers, to assess the relevance to their own work.

A COS has previously been defined as an agreed standardised set of outcomes that should be measured and reported, as a minimum, in all clinical trials in specific areas of health or health care [2]. COS are being developed for settings other than clinical trials. Although previous recommendations have been made regarding the reporting of a Delphi survey component of a COS study [5], it is timely to gain wider consensus given the increasing activity in this area, particularly because various other methods and components are incorporated in the COS development process [1]. In this article, we present the results of a research project, in which the objective was to develop a guideline (Core Outcome Set–STAndards for Reporting [COS-STAR]) for the reporting of studies developing COS using an approach proposed by the Enhancing the QUAlity and Transparency Of health Research (EQUATOR) Network [6]. The reporting checklist is relevant regardless of the consensus methodology used in the development of the COS and can be applied to COS developed for effectiveness trials, systematic reviews, or routine care [7].

## Terminology

A COS describes what should be measured in a particular research or practice setting, with subsequent work needed to determine how each outcome should be defined or measured. A previous review found that only 38% of published studies contained recommendations about how to measure the outcomes in the COS [4]. This reporting guideline was developed to address this first stage of development, namely, what should be measured.

## Ethical Approval

The University of Liverpool Ethics Committee was consulted and granted ethical approval for this study (Reference RETH000841). Informed consent was assumed if a participant responded to the Delphi survey or agreed to attend the consensus meeting.

## Development of the COS-STAR Statement

A full protocol outlining the Delphi procedures for the COS-STAR study was published elsewhere [8], including the intention to produce an associated Explanation and Elaboration (E+E) document in which the meaning and rationale for each checklist item is given.

A preliminary list of 48 reporting items was developed from a previous systematic review of COS studies involving a Delphi survey [5], the personal experiences of COS development, and reporting by the project management group (The COS-STAR Group). This preliminary list of potential reporting items was included in an international two-round Delphi survey in order to ascertain the importance of these reporting items.

The Delphi survey involved four key stakeholder groups, chosen to encompass aspects of COS development, reporting, and uptake. Invitations by email were sent to the following: (i) lead authors of 196 published COS studies in the Core Outcome Measures in Effectiveness Trials (COMET) database, with a request to also forward on the invitation to any methodologist involved; (ii) editors of 250 journals in which COS studies have been published and 70 journals involved with CoRe Outcomes in WomeN’s health ([CROWN], http://www.crown-initiative.org/), an initiative endorsing the uptake of COS; (iii) potential users of COS who were (a) principal investigators of open phase III/IV trials, commercial or non-commercial, registered on clinicaltrials.gov (20% random sample from 8,449 registered trials) or (b) 76 Coordinating Editors from 53 Cochrane Review Groups; and (iv) 33 patient representatives from previous COMET workshops and the COMET Public and Patient Participation, Involvement and Engagement (PoPPIE) Working Group. It did not prove possible to invite clinical guideline developers listed on the Guidelines International Network website (http://www.gin2015.net/about/) due to this being a member organisation, so a question about involvement in clinical guideline development was added to the survey instead. The aim was to recruit as many individuals from each stakeholder group as possible for the Delphi exercise. Participants were sent a personalised email outlining the project as described in the study protocol together with a copy of the first systematic review of COS studies [4].

Delphi participants rated the importance of each reporting item on a scale from 1 (not important) to 9 (critically important) as defined in the protocol [8]. In round one of the Delphi study, participants could suggest new reporting items to be included in the second round. In round two, each participant who participated in round one was shown the number of respondents and distribution of scores for each item for all stakeholder groups separately, together with their own score from round one. An additional nine reporting items were suggested in round one of the Delphi exercise and were scored in round two. Consensus, defined a priori [8], was achieved if at least 70% of the voting participants from each stakeholder group scored between 7 and 9. COS developers (*n* = 25), COS users (*n* = 107), medical journal editors (*n* = 40), and patient representatives (*n* = 11) participated in both rounds, with 13 individuals also having been involved in clinical guideline development. The variable number of respondents per stakeholder group did not affect the results, because feedback in round two was presented by group. The Delphi process was conducted and managed using DelphiManager software developed by the COMET Initiative (http://www.comet-initiative.org/delphimanager/). The anonymised data from both rounds of the Delphi process, itemised by stakeholder group, are available in S1 Delphi Data.

The consensus meeting was a one-day event held in London, United Kingdom, in January 2016, with 17 international participants, including COS developers (*n* = 6), medical journal editors (*n* = 4), COS users (*n* = 5) [trialists (*n* = 1), Cochrane systematic review co-editors (*n* = 2), and clinical guideline developers (*n* = 2)], and patient representatives (*n* = 2). Individuals were selected to be invited to the meeting using the following broad principles: (i) Delphi participants who completed both rounds; (ii) a balance across the four stakeholder groups; (iii) a balance amongst COS developers of those using Delphi surveys and those using other methods; (iv) a balance across COS users of trialists, systematic reviewers, and guideline developers; and (v) a reasonable geographic spread. Initial invitations were determined by the authors based on their knowledge of individuals’ expertise. If an individual could not attend, they were replaced by someone else from the same stakeholder group wherever possible.

The roles of each participant were not mutually exclusive, and there was a mix of clinical and methodological experience. The objective of the meeting was to discuss and vote on the series of 57 reporting items thought to be potentially important for inclusion in the COS-STAR checklist. Three additional participants (one facilitator and two note takers) attended the meeting but did not participate in the discussion or voting.

The Delphi results for all 57 reporting items were presented to the consensus meeting participants prior to and during the meeting (S1 Consensus Matrix Delphi). A copy of the consensus meeting slides showing the response rates, geographical distribution, and Delphi round two results for each stakeholder group can be found in S1 Consensus Meeting Presentation. Although the response rate to the survey was low, over 86% (183/214) of round one participants completed round two, with no evidence of attrition bias between rounds (S1 Consensus Meeting Presentation).

Members of the COS-STAR group (study authors) who could not attend the meeting were contacted in advance to discuss the results; their comments were documented and fed into the meeting discussion. Following presentation of the Delphi result for each potential reporting item, the item was discussed and then voted on by the meeting participants, and retained if more than 70% of the voting participants (at least 12 of the 17 voting participants) scored between 7 and 9. Voting was undertaken using OMBEA response (http://www.ombea.com/). During discussion of the second Consensus Process item, “Description of what information was presented to participants about the consensus process at its start,” one participant commented on the absence of an item related to ethical approval. The group agreed to vote on this, and consensus was achieved that this item should be included in the reporting guideline (S1 Consensus Meeting Critical Scores). This issue has become more relevant as the inclusion of patients as participants in COS development has increased.

Discussion of the format of the reporting checklist was given consideration by the management group. It was confirmed that the required items would be relevant to the reporting of a COS regardless of whether it had been developed for effectiveness trials, systematic reviews, or routine care. A word of warning was given by those participants at the meeting with previous experience of reporting guideline development: avoid making the first guideline too stringent and risk developers not using it. The need to merge some reporting items and create some sub-items was noted, together with suggestions for an explanatory document to enhance the usefulness of the final COS-STAR checklist. Specifically, the following amendments were made by the COS-STAR group after the meeting:

(1) Participants (methods): The four items under this topic were considered to overlap. The suggestion was made, and accepted by the group, that one item covering the sub-items of who, how, and why would be more meaningful.
(2) Consensus process: There was general agreement that although all five items under this topic could provide useful information, some were too specific to be included, acknowledging that there is no gold standard approach. The suggestion was made, and accepted by the group, that one item covering the description of the consensus process would more likely be followed.
(3) Outcome scoring: a suggestion was made to include both aspects in a single item.
(4) Participants (results): a suggestion was made to combine these issues into a single item.
(5) Outcome results: there was general agreement that these multiple items were too detailed and should be merged together, elaborating on the issues in the E+E document (S1 Explanation and Elaboration).
(6) Limitations: it was agreed that it was good practice to include an item related to limitations and include the examples provided in the E+E document.

Following the meeting, a draft of the COS-STAR checklist was circulated to the COS-STAR group and the remainder of the consensus meeting attendees. All comments and revisions were taken into consideration and the checklist revised accordingly. The process of obtaining feedback and refining the checklist was repeated until no further changes were needed.

The comprehension of the final checklist items was reviewed by two expert guideline developers (DGA, DM). Testing was undertaken by two COS developers who were writing up their COS studies. Two researchers currently developing COS also reviewed the statement. Testers were independent of the COS-STAR development process. Feedback from this exercise informed the final version of the COS-STAR checklist.

## The COS-STAR Statement

The 18-item COS-STAR checklist presented in Table 1 applies to COS development studies for which the aim is to decide which outcomes should be included in the COS and does not extend to cover the reporting of work to determine how those outcomes should be defined or measured. The checklist aims to cover the minimum reporting requirements related to the background, scope, methods, results, conclusion, and limitations of such studies. In the accompanying E+E document (S1 Explanation and Elaboration), explanations are provided for the meaning and rationale for each checklist item. The checklist is designed to be applicable regardless of consensus methodology used to develop the COS (inclusive of mixed methods) and the various participant groups who may have been involved in selecting outcomes (inclusive of patient representatives), as identified in a previous systematic review [4]. The COS-STAR checklist provides guidance for minimal COS study reporting, but, in certain circumstances, additional reporting items may be warranted at the discretion of the study authors. For example, study authors may wish to describe the steps in deciding how to measure the core outcomes if this was considered [9].

**Table 1:** Core Outcome Set-STandards for Reporting: The COS-STAR Statement.

SECTION/TOPIC	ITEM No.	CHECKLIST ITEM
**TITLE/ABSTRACT**		
Title	1a	Identify in the title that the paper reports the development of a COS
Abstract	1b	Provide a structured summary
**INTRODUCTION**		
Background and Objectives	2a	Describe the background and explain the rationale for developing the COS.
	2b	Describe the specific objectives with reference to developing a COS.
Scope	3a	Describe the health condition(s) and population(s) covered by the COS.
	3b	Describe the intervention(s) covered by the COS.
	3c	Describe the setting(s) in which the COS is to be applied.
**METHODS**		
Protocol/Registry Entry	4	Indicate where the COS development protocol can be accessed, if available, and/or the study registration details.
Participants	5	Describe the rationale for stakeholder groups involved in the COS development process, eligibility criteria for participants from each group, and a description of how the individuals involved were identified.
Information Sources	6a	Describe the information sources used to identify an initial list of outcomes.
	6b	Describe how outcomes were dropped/combined, with reasons (if applicable).
Consensus Process	7	Describe how the consensus process was undertaken.
Outcome Scoring	8	Describe how outcomes were scored and how scores were summarised.
Consensus Definition	9a	Describe the consensus definition.
	9b	Describe the procedure for determining how outcomes were included or excluded from consideration during the consensus process.
Ethics and Consent	10	Provide a statement regarding the ethics and consent issues for the study.
**RESULTS**		
Protocol Deviations	11	Describe any changes from the protocol (if applicable), with reasons, and describe what impact these changes have on the results.
Participants	12	Present data on the number and relevant characteristics of the people involved at all stages of COS development.
Outcomes	13a	List all outcomes considered at the start of the consensus process.
	13b	Describe any new outcomes introduced and any outcomes dropped, with reasons, during the consensus process.
COS	14	List the outcomes in the final COS.
**DISCUSSION**		
Limitations	15	Discuss any limitations in the COS development process.
Conclusions	16	Provide an interpretation of the final COS in the context of other evidence, and implications for future research.
**OTHER INFORMATION**		
Funding	17	Describe sources of funding/role of funders.
Conflicts of Interest	18	Describe any conflicts of interest within the study team and how these were managed.

## Discussion

The COS-STAR checklist was developed using an approach that has been recommended for developing medical reporting guidelines [6]. The intention of the COS-STAR checklist is to promote the transparency and completeness of reporting of COS studies such that COS users can judge whether the recommended set is relevant to their work. For example, although the adequacy of the description of the scope of a COS is increasing [1], further improvement is needed in order for uptake to be maximised and, in turn, assessed.

The COS-STAR E+E document (S1 Explanation and Elaboration) was developed to provide an explanation of each of the COS-STAR checklist items and to provide a framework for good reporting practices, with examples, for those interested in conducting and reporting COS development work. It follows a similar format to that used in other explanatory documents [10,11]. The E+E document also describes the endorsement and implementation strategies planned for the COS-STAR Statement.

COS-STAR is not a quality assessment tool and should not be used in this way, for example, to compare the validity of similar related COS. Similarly, the checklist does not make recommendations about which methodology should be used or which stakeholder groups to include to reach consensus in COS development projects; guidance on such issues can be found elsewhere [2,12]. As an example, several studies have looked at developing COS in childhood asthma, each using different methodology and proposing slight variations in the core outcomes [13–16]. The COS-STAR Statement would not distinguish which of these COS should be used, although it may be useful for critical appraisal of published COS.

As with similar reporting guidelines [17] that have undergone several revisions, COS-STAR is an evolving guideline and may well require modification in the future. The consensus meeting participants acknowledged that there is limited empirical evidence and methodological development relating to some of the reporting items that were considered for inclusion and chose to exclude those items until these issues are better understood. As an example, there is some evidence that the method of feedback does influence how people score outcomes [18], which perhaps suggests that this is important information to report. However, until there is better guidance on how COS developers should present feedback, the item was excluded from the current reporting checklist.

The guideline may also require modification as new stakeholder groups with relevant interests and experience emerge. For example, regulators have recently recommended the use of COS in trials of medicinal products in patients with asthma [19] and will be encouraged to provide feedback on the statement. The geographical spread of participants in the Delphi survey, and consequently the consensus meeting, is representative of COS study developers, being predominantly North American and European [4]. This is recognised as a limitation, however, of both COS study development and this reporting guideline, given the equal relevance to low- and middle-income countries. Patient representatives, rather than patients, were invited to participate. Due to this being a relatively complex area of methodology, individuals known to have had some level of involvement with COS development were selected, thereby increasing their understanding. As patient involvement and participation in COS development increases, their contribution to a future revision of this reporting guideline will be sought. An important objective of the COMET Initiative is to promote wider involvement.

Although the acceptance rate to the Delphi invitation may appear low, participation in round two was above 85% in each stakeholder group, with no evidence of attrition bias (S1 Consensus Meeting Presentation). Readers are invited to submit comments, criticisms, experiences, and recommendations via the COMET website (http://www.comet-initiative.org/contactus), which will be considered for future refinement of the COS-STAR Statement.

## Supporting Information

S1 Consensus Matrix Delphi Consensus matrix for round one and round two of the COS-STAR Delphi survey. (DOCX)

S1 Consensus Meeting Critical Scores Percentages of participants scoring each item as critical for inclusion (7–9) at the COS-STAR meeting. (DOCX)

S1 Consensus Meeting Presentation Consensus meeting slides presented at the COS-STAR consensus meeting. (PPT)

S1 Delphi Data Delphi Data for round one and round two of the COS-STAR Delphi survey. (XLSX)

S1 Explanation and Elaboration Explanation and Elaboration document for COS-STAR. (DOCX)

## Abbreviations

COMET: Core Outcome Measures in Effectiveness Trials
COS: core outcome sets
COS-STAR: Core Outcome Set-STAndards for Reporting
CROWN: CoRe Outcomes in WomeN’s health
E+E: Explanation and Elaboration
EQUATOR: Enhancing the QUAlity and Transparency Of health Research
PoPPIE: People and Public Participation, Involvement and Engagement

## References

[1] Gorst SL, Gargon E, Clarke M, Blazeby JM, Altman DG, Williamson PR. Choosing Important Health Outcomes for Comparative Effectiveness Research: An Updated Review and User Survey. *PLoS ONE*. 2016; 11(1): e0146444 10.1371/journal.pone.0146444 26785121 PMC4718543

[2] Williamson PR, Altman DG, Blazeby JM, Clarke M, Devane D, Gargon E et al Developing core outcome sets for clinical trials: issues to consider. *Trials*. 2012; 13:132 10.1186/1745-6215-13-132 22867278 PMC3472231

[3] COMET Initiative. 2016. http://www.comet-initiative.org/. [Accessed 5 May 2016].

[4] Gargon E, Gurung B, Medley N, Altman DG, Blazeby JM, Clarke M et al Choosing Important Health Outcomes for Comparative Effectiveness Research: A Systematic Review. *PLoS ONE*. 2014; 9(6): e99111 10.1371/journal.pone.0099111 24932522 PMC4059640

[5] Sinha I, Smyth RL, Williamson PR. Using the Delphi technique to determine which outcomes to measure in clinical trials: recommendations for the future based on a systematic review of existing studies. *PLoS Med*. 2011; 8(1): e1000393 10.1371/journal.pmed.1000393 21283604 PMC3026691

[6] Moher D, Schulz KF, Simera I, Altman DG. Guidance for developers of health research reporting guidelines. *PloS Med*. 2010; 7(2): e1000217 10.1371/journal.pmed.1000217 20169112 PMC2821895

[7] Clarke M and Williamson PR. Core outcome sets and systematic reviews. *Systematic Reviews*. 2016; 5:11 10.1186/s13643-016-0188-6 26792080 PMC4719739

[8] Kirkham JJ, Gorst S, Altman DG, Blazeby J, Clarke M, Devane D et al COS-STAR: a reporting guideline for studies developing core outcome sets (protocol). *Trials*. 2015; 16:373 10.1186/s13063-015-0913-9 26297658 PMC4546355

[9] Schmitt J, Spuls PI, Thomas KS, Simpson E, Furue M, Deckert S et al The Harmonising Outcome Measures for Eczema (HOME) statement to assess clinical signs of atopic eczema in trials. *J Allergy Clin Immunol*. 2014; 134:800–807. 10.1016/j.jaci.2014.07.043 25282560

[10] Moher D, Hopewell S, Schulz KF, Montori V, Gøtzsche PC, Devereaux PJ et al CONSORT 2010 Explanation and Elaboration: updated guidelines for reporting parallel group randomised trial. *BMJ*. 2010; 340:c869 10.1136/bmj.c869 20332511 PMC2844943

[11] Liberati A, Altman DG, Tetzlaff J, Mulrow C, Gøtzsche PC, Ioannidis J et al The PRISMA Statement for reporting systematic reviews and meta-analyses of studies that evaluate health care interventions: Explanation and Elaboration. *PLoS Med*. 2009;6(7):e1000100 10.1371/journal.pmed.1000100 19621070 PMC2707010

[12] Schmitt J, Apfelbacher C, Spuls PI, Thomas KS, Simpson E, Furue M et al The Harmonizing Outcome Measures for Eczema (HOME) roadmap: A methodological framework to develop core sets of outcome measurements in dermatology. *Journal of Investigative Dermatology*. 2015; 135 (1):24–30. 10.1038/jid.2014.320 25186228

[13] Smith MA, Leeder SR, Jalaludin B, Smith WT. The asthma health outcome indicators study. *Australian and New Zealand Journal of Public Health*. 1996; 20 (1): 69–75. 10.1111/j.1467-842X.1996.tb01340.x 8799071

[14] Sinha I, Gallagher R, Williamson PR, Smyth RL. Development of a core outcome set for clinical trials in childhood asthma: a survey of clinicians, parents, and young people. *Trials*. 2012; 13:103 10.1186/1745-6215-13-103 22747787 PMC3433381

[15] Reddel HK, Taylor DR, Bateman ED, Boulet LP, Boushey HA, Busse WW et al An official American Thoracic Society/European Respiratory Society statement: asthma control and exacerbations: standardizing endpoints for clinical asthma trials and clinical practice. *Am J Respir Crit Care Med*. 2009; 180(1):59–99. 10.1164/rccm.200801-060ST 19535666

[16] Busse WW, Morgan WJ, Taggart V, Togias A. Asthma outcomes workshop: overview. *Journal of Allergy & Clinical Immunology*. 2012; 129 (3) Suppl: S1–8. 10.1016/j.jaci.2011.12.985 22386504 PMC4259286

[17] Schulz KF, Altman DG, Moher D, for the CONSORT Group. CONSORT 2010 Statement: updated guidelines for reporting parallel group randomised trials. *PLoS Med*. 2010;7(3): e1000251 10.1371/journal.pmed.1000251 20352064 PMC2844794

[18] Brookes S, Macefield R, Williamson P, McNair A, Potter S, Blencowe N et al Three nested RCTs of dual or single stakeholder feedback within Delphi surveys during core outcome and information set development. *Trials*. 2015 11 16;16(Suppl 2):P51 10.1186/s13063-016-1479-x PMC4989325 27534622

[19] Committee for Medicinal Products for Human Use (CHMP) (2015). Guideline on the clinical investigation of medicinal products for the treatment of asthma. CHMP/EWP/2922/01 Rev.1. http://www.ema.europa.eu/docs/en_GB/document_library/Scientific_guideline/2015/12/WC500198877.pdf

