# Supplementary material for: Core Outcome Set–STAndards for Reporting: The COS-STAR Statement
Source: PLoS Med. 2016 Oct 18;13(10):e1002148. doi: 10.1371/journal.pmed.1002148 (PMC5068732; doi:10.1371/journal.pmed.1002148)
Supplement: S1 Consensus Meeting Critical Scores — (DOCX) [file pmed.1002148.s002.docx]

**COS-STAR Meeting: Number and percentages of participants scoring each item as critical for inclusion (7-9)**

| **TITLE:** Identification that paper reports development of a core outcome set | (17/17) 100% |
| --- | --- |
| **STRUCTURED SUMMARY:** Provide a structured summary | (17/17) 100% |
| **RATIONALE:** Rationale for the development of a core outcome set (examples: there is no current core outcome set in area; or a review of outcomes in previous trials revealed diversity in outcomes measured to date) | (17/17) 100% |
| **OBJECTIVES:** Purpose of core outcome set to be developed, including explanations and terminology used (examples: the development of a core set of domains; or the development of a core set of measurement instruments) | (17/17) 100% |
| **HEALTH CONDITION AND CONTEXT:** (part of the scope of the core outcome set): Health condition(s)/context covered by core outcome set (examples: treatment of rheumatoid arthritis, prevention of pre-eclampsia; screening for cancer) | (17/17) 100% |
| **POPULATIONS:** (part of the scope of the core outcome set): Population(s) covered by core outcome set (examples: patients with advanced disease; or children) | (17/17) 100% |
| **INTERVENTIONS:** (part of the scope of the core outcome set): Types of intervention(s) covered by core outcome set (examples: all interventions, drug therapy, surgical interventions; rehabilitation) | (16/17) 94% |
| **SETTING:** (part of the scope of the core outcome set): Setting for the application of the core outcome set (examples: for application in research studies; or for use in routine care) | (12/17) 71% |
| **RESEARCH DESIGN:** Research type(s) the core outcome set was developed for (examples: effectiveness trial; efficacy trial; systematic review) | (7/17) 41% |
| **PROTOCOL:** Details of any protocol and where it can be accessed | (16/17) 94% |
| **PARTICIPANTS:** Eligibility criteria for participants including stakeholder groups and the rationale for involving them (examples: health care professionals for the care of patients with the condition; or patients who are members of a particular association) | (16/17) 94% |
| **PARTICIPANTS:** Description of how stakeholders were identified (examples: UK centres involved in clinical care of patients with the condition; patients attending specific clinics) | (11/17) 65% |
| **PARTICIPANTS:** Description of how members of each stakeholder group were chosen from within the stakeholder group and how many invited (examples: corresponding authors of all trials published in a specific area; or steering group nominations) | (9/17) 53% |
| **PARTICIPANTS:** Description of how individuals were invited to take part in the consensus process (examples: emails or letters sent directly to the participants; emails sent via a third party, e.g. via the managing editor of a Cochrane group) | (5/17) 29% |
| **INFORMATION SOURCES:** Description of all information sources used to generate an initial list of outcomes (if used) to participants (examples: all outcomes listed in included studies from a relevant systematic review; direct contact with trialists; focus groups; interviews with patients/carers/parents) | (16/17) 94% |
| **INFORMATION SOURCES:** Description of how outcomes were classified/dropped/combined to make the list manageable | (17/17) 100% |
| **CONSENSUS PROCESS:** Description of how the consensus process was undertaken (examples: Delphi survey of health care professionals (electronic or postal); interviews with patients; face to face discussions with stakeholder representatives) | (17/17) 100% |
| **CONSENSUS PROCESS:** Description of what information was presented to participants about the consensus process at its start (examples: background to the project; importance of developing a core outcome set in this area; process (e.g. what are participants expected to do); number of Delphi rounds (if Delphi used); timescales) | (3/17) 18% |
| **CONSENSUS PROCESS:** Description of what participants were asked to do in each component or round (if multiple rounds used) (examples: review scores from a previous round; re-score outcomes after reviewing the opinions of other stakeholders; introduce new outcomes that are not currently listed in the process) | (8/17) 47% |
| **CONSENSUS PROCESS:** Description of how the overall group responses were fed back to participants(examples: presentation of own stakeholder group results only; presentation of data from all stakeholder groups (averaged or not averaged)) | (9/17) 53% |
| **CONSENSUS PROCESS:** Description of whether non-responders (or partial responders) were invited into subsequent rounds of the consensus process | (5/17) 29% |
| **OUTCOME SCORING:** Description of how outcomes were scored during the consensus exercise (examples: 1 to 9 VAS scale with option for ‘unable to score’; traffic light system for young children) | (15/17) 88% |
| **OUTCOME SCORING:** Description of how scores were summarised across participants during each stage of the consensus process (examples: % scoring (each score); median score; % above a specific score) | (15/17) 88% |
| **DEFINITION OF CONSENSUS:** Description of the consensus definition (examples: % scoring a specific score (e.g. % scoring 7 or above); median score above a specific value; absence of specific scores) | (17/17) 100% |
| **DEFINITION OF CONSENSUS:** Description of the procedure for determining how outcomes were included or excluded from consideration at each stage (examples: all outcomes carried forward to each round irrespective of results between rounds; outcomes excluded after each round if the consensus criteria is not reached for all stakeholder groups) | (16/17) 94% |
| **SOFTWARE:** Description of any software used to administer any survey or analyse any results (examples: Survey Monkey; bespoke survey) | (1/17) 6% |
| **PARTICIPANTS:** Total number of participants invited from each stakeholder group (as appropriate) | (11/17) 65% |
| **PARTICIPANTS:** Description of the participant characteristics | (15/17) 88% |
| **PARTICIPANTS:** Total number of participants who completed each component or round from each stakeholder group (as appropriate) | (14/17) 82% |
| **OUTCOMES:** List all the outcomes considered at the outset of the process | (15/17) 88% |
| **OUTCOMES:** Description of any new outcomes introduced into the consensus process after it began and how these were introduced (examples: healthcare professionals suggested the following additional outcomes (list them…) in round 1 of the Delphi survey; patients suggested the following additional outcomes (list them…) when interviewed | (16/17) 94% |
| **OUTCOMES:** Description of any outcomes dropped between rounds, and any reasons for this (examples: the following outcomes (list them…) were dropped after the final round of the Delphi survey as the consensus criteria was not met for any of the stakeholder groups) | (15/17) 88% |
| **OUTCOMES:** Results for each outcome in each round | (0/17) 0% |
| **OUTCOMES:** Description of the group responses for each outcome in the final round | (2/17) 12% |
| **CORE OUTCOME SET:** List of outcomes included in the final core outcome set | (17/17) 100% |
| **RELEVANCE:** Discussion of the relevance of the core outcome set to key groups (examples: patients and the public; healthcare providers; policy makers) | (3/17) 18% |
| **BARRIERS:** Discussion of the barriers to implementation of the core outcome set (examples: resource issues; long term outcomes; specialised equipment to measure outcomes) | (2/17) 12% |
| **LIMITATIONS:** Discussion of the limitations in representativeness from stakeholder groups (examples: missing stakeholder groups in the development process; low numbers of participants in particular groups; over-estimation of certain opinions) | (7/17) 41% |
| **LIMITATIONS:** Discussion of the limitations in representativeness in terms of geographic coverage (examples: the core outcome set may only be relevant for use in the UK as only UK participants were involved in the consensus process) | (5/17) 29% |
| **LIMITATIONS:** Discussion of any other limitations in the core outcome set development process | (17/17) 100% |
| **ATTRITION:** Discussion of how attrition between rounds of the consensus process may have impacted on the results (examples: Delphi scores were higher for the participants that responded in all rounds of the Delphi compared to those that only responded in one round, suggesting that the level of consensus achieved in the final round maybe overestimated) | (2/17) 12% |
| **RISK OF BIAS:** Discussion of any changes from the protocol, the reasons for these changes, and how these changes may have impacted on the results | (13/17) 76% |
| **CONCLUSIONS:** Interpretation of the results in the context of other evidence, and implications for practice and future research | (17/17) 100% |
| **UPDATE:** Describe any plans to review, re-evaluate and update the core outcome set (examples: the proposed core outcome set will be re-evaluated at bi-annual meetings to ensure that the outcomes remain relevant in accordance to current practice) | (0/17) 0% |
| **IMPLEMENTATION/UPTAKE:** Describe any plans to facilitate the uptake of the core outcome set (examples: inclusion in guidance for the conduct of research in the health area; use by a Cochrane review group for its reviews in the specific health area) | (2/17) 12% |
| **MEASUREMENT INSTRUMENTS:** Description of the next steps to select measurement instruments for the core outcomes proposed | (1/17) 6% |
| **FUNDING:** Description of the sources of funding and the role of the funder in the study | (17/17) 100% |
| **CONFLICTS OF INTEREST:** Description of any conflicts of interest within the study team | (17/17) 100% |
| **PARTICIPANTS:** Description of the efforts made to include underrepresented/hard to reach patients/stakeholder groups [suggested by patient representative] | (0/17) 0% |
| **CONSENSUS PROCESS:** Description of the efforts to make the study material patient friendly and understandable [suggested by a COS developer] | (0/17) 0% |
| **CONSENSUS PROCESS:** Description of how patients were enabled to participate in the consensus process [suggested by patient representative] | (4/17) 24% |
| **CONSENSUS PROCESS:** Description of the aspects of the consensus process that differed according to stakeholder group [suggested by patient representative] | (1/17) 6% |
| **CONSENSUS PROCESS:** Reason/explanation for the choice of consensus definition [suggested by COS user] | (5/17) 29% |
| **METHODS:** Description of any lessons learnt when developing the core outcome set [suggested by patient representative] | (1/17) 6% |
| **METHODS:** Descriptions of any challenges of involving various stakeholder groups and how these were overcome [suggested by patient representative] | (1/17) 6% |
| **DISSEMINATION:** Description of plans for disseminating study results to all participants and wider patient community [suggested by patient representative] | (1/17) 6% |
| **APPENDIX:** Inclusion of the participant information sheet (PIS) [suggested by patient representative] | (0/17) 0% |
| **ETHICS AND CONSENT:** Statement regarding the ethics and consent issues for the study | (12/17) 71% |
