# Supplementary material for: Core Outcome Set–STAndards for Reporting: The COS-STAR Statement
Source: PLoS Med. 2016 Oct 18;13(10):e1002148. doi: 10.1371/journal.pmed.1002148 (PMC5068732; doi:10.1371/journal.pmed.1002148)
Supplement: S1 Consensus Meeting Presentation — (PPT) [file pmed.1002148.s003.ppt]

## Slide 1
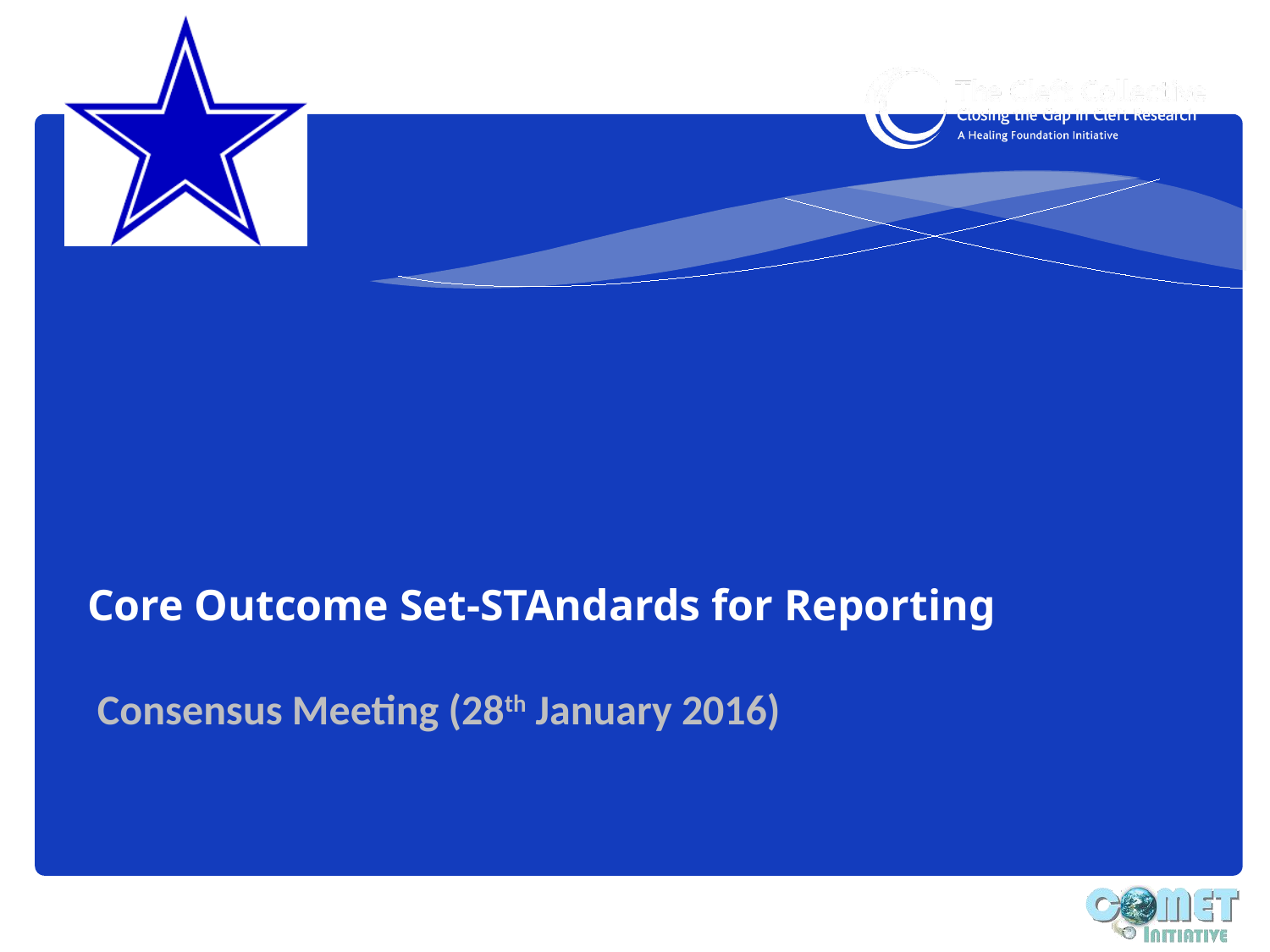

COS-STAR
# Core Outcome Set-STAndards for Reporting
Consensus Meeting (28th January 2016)

## Slide 2
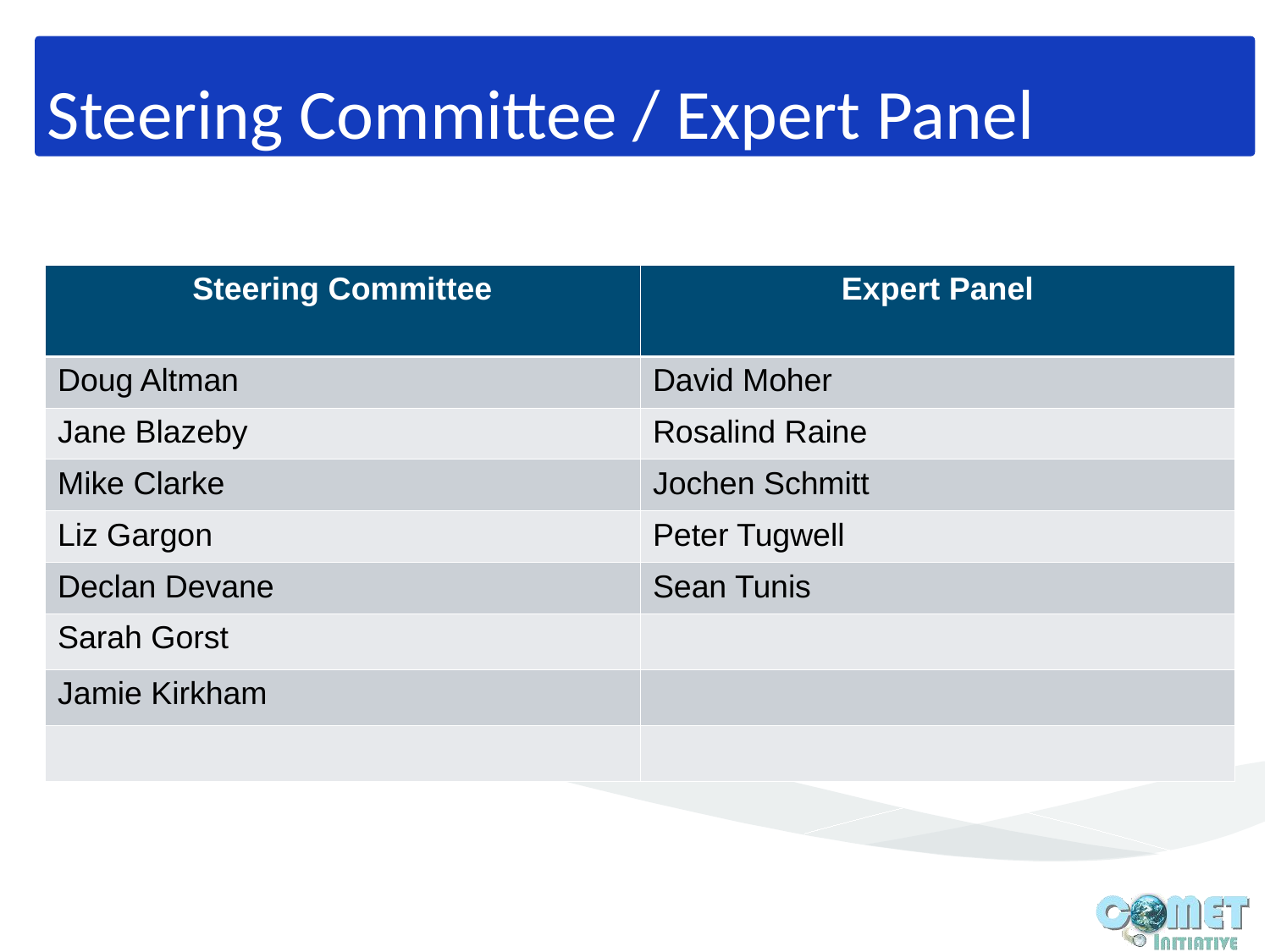

# Steering Committee / Expert Panel
| Steering Committee | Expert Panel |
| --- | --- |
| Doug Altman | David Moher |
| Jane Blazeby | Rosalind Raine |
| Mike Clarke | Jochen Schmitt |
| Liz Gargon | Peter Tugwell |
| Declan Devane | Sean Tunis |
| Sarah Gorst | |
| Jamie Kirkham | |
| | |

## Slide 3
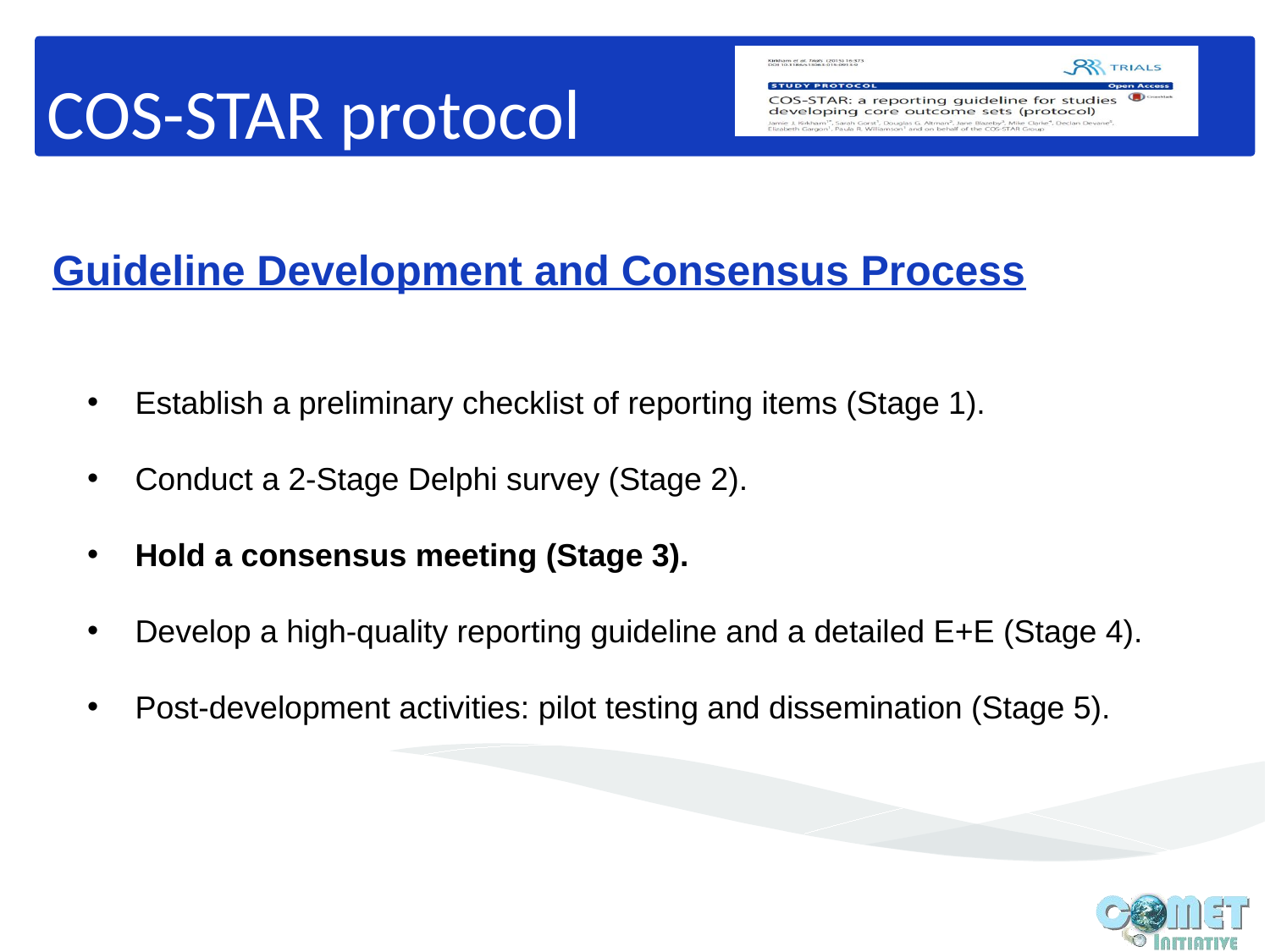

# COS-STAR protocol
Guideline Development and Consensus Process
Establish a preliminary checklist of reporting items (Stage 1).
Conduct a 2-Stage Delphi survey (Stage 2).
Hold a consensus meeting (Stage 3).
Develop a high-quality reporting guideline and a detailed E+E (Stage 4).
Post-development activities: pilot testing and dissemination (Stage 5).

## Slide 4
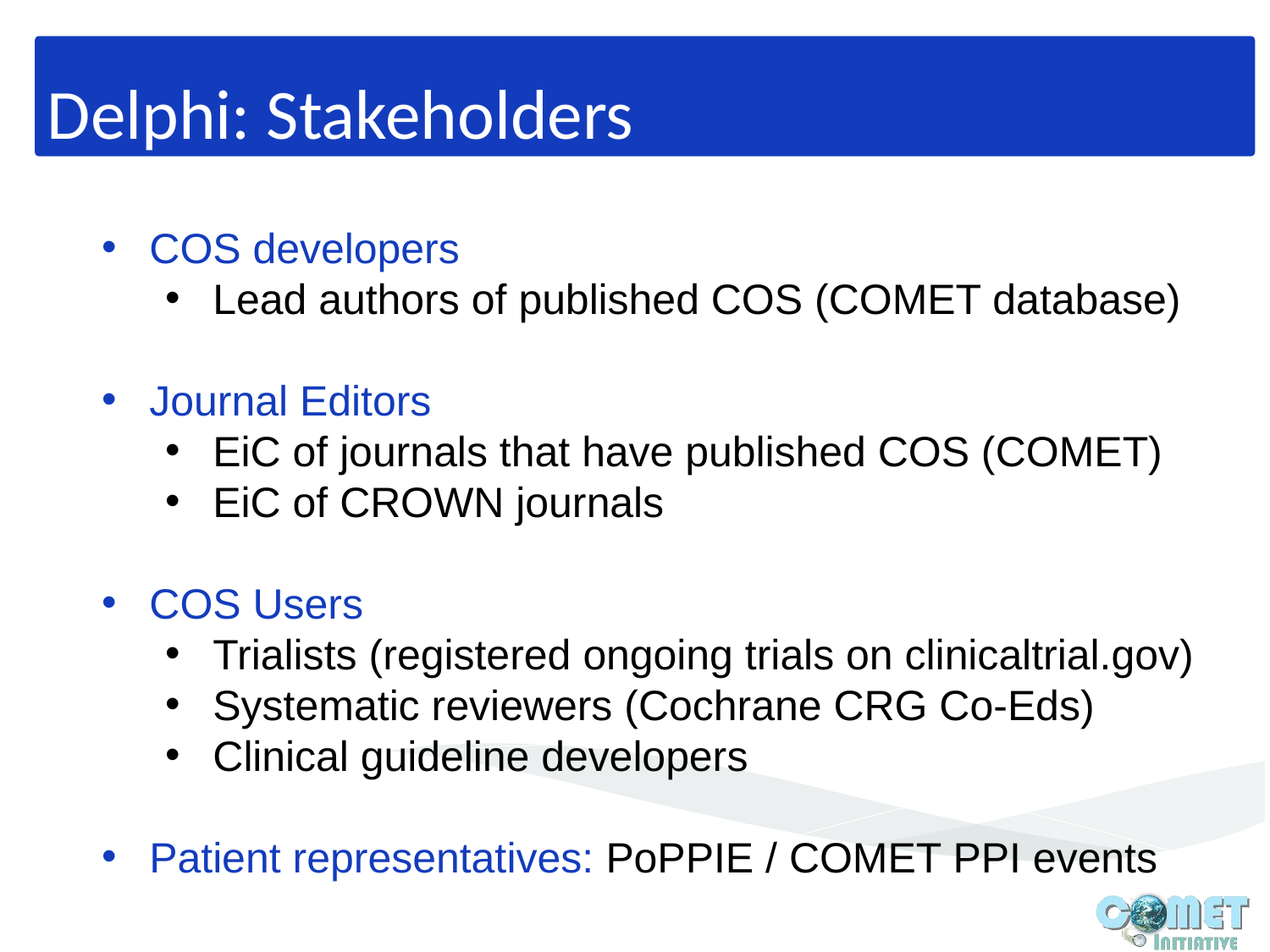

# Delphi: Stakeholders
COS developers
Lead authors of published COS (COMET database)
Journal Editors
EiC of journals that have published COS (COMET)
EiC of CROWN journals
COS Users
Trialists (registered ongoing trials on clinicaltrial.gov)
Systematic reviewers (Cochrane CRG Co-Eds)
Clinical guideline developers
Patient representatives: PoPPIE / COMET PPI events

## Slide 5
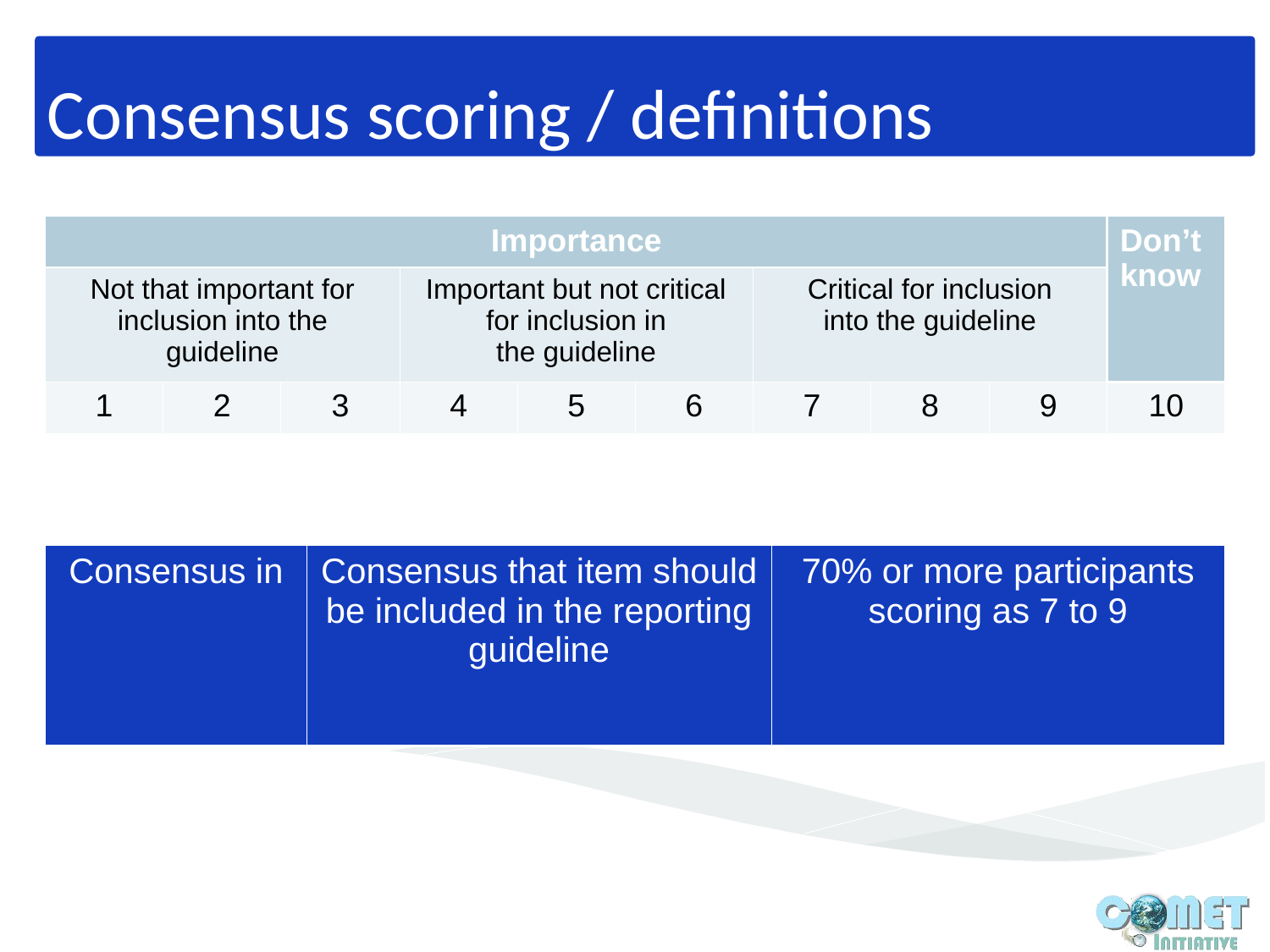

# Consensus scoring / definitions
| Importance | | | | | | | | | Don’t know |
| --- | --- | --- | --- | --- | --- | --- | --- | --- | --- |
| Not that important for inclusion into the guideline | | | Important but not critical for inclusion in the guideline | | | Critical for inclusion into the guideline | | | |
| 1 | 2 | 3 | 4 | 5 | 6 | 7 | 8 | 9 | 10 |
| Consensus in | Consensus that item should be included in the reporting guideline | 70% or more participants scoring as 7 to 9 |
| --- | --- | --- |

## Slide 6
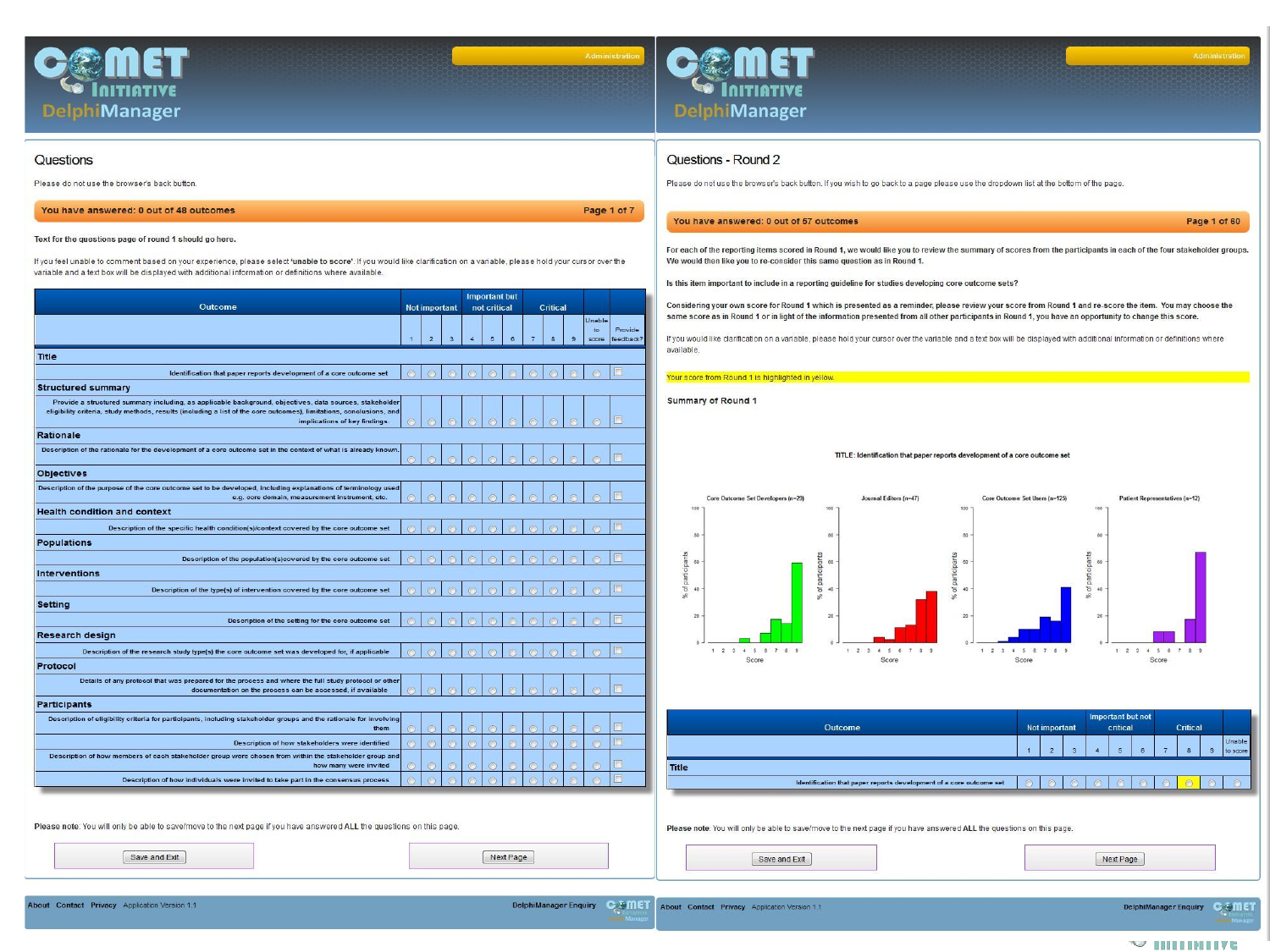

## Slide 7
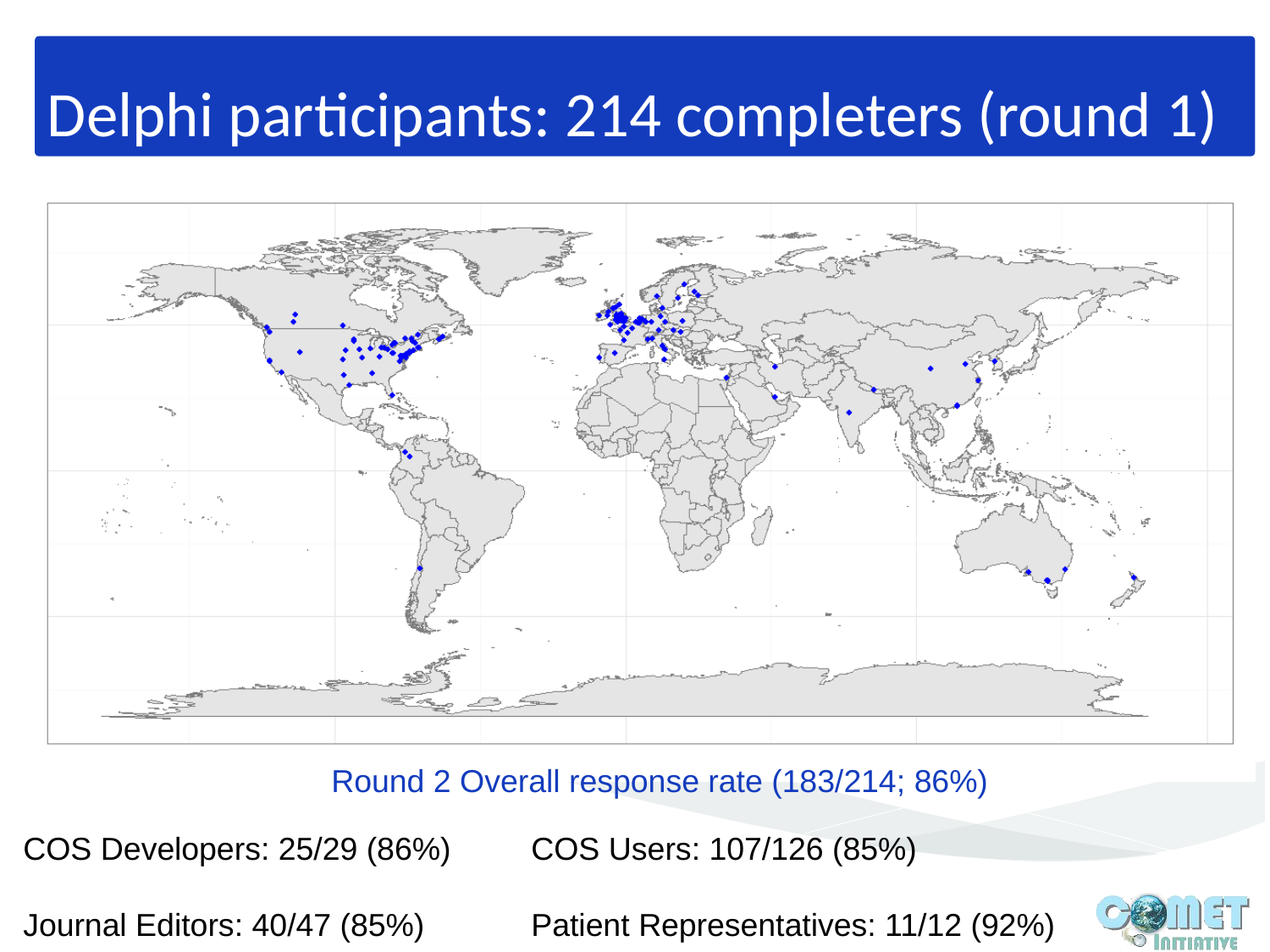

# Delphi participants: 214 completers (round 1)
Round 2 Overall response rate (183/214; 86%)
COS Developers: 25/29 (86%)	COS Users: 107/126 (85%)
Journal Editors: 40/47 (85%)	Patient Representatives: 11/12 (92%)

## Slide 8
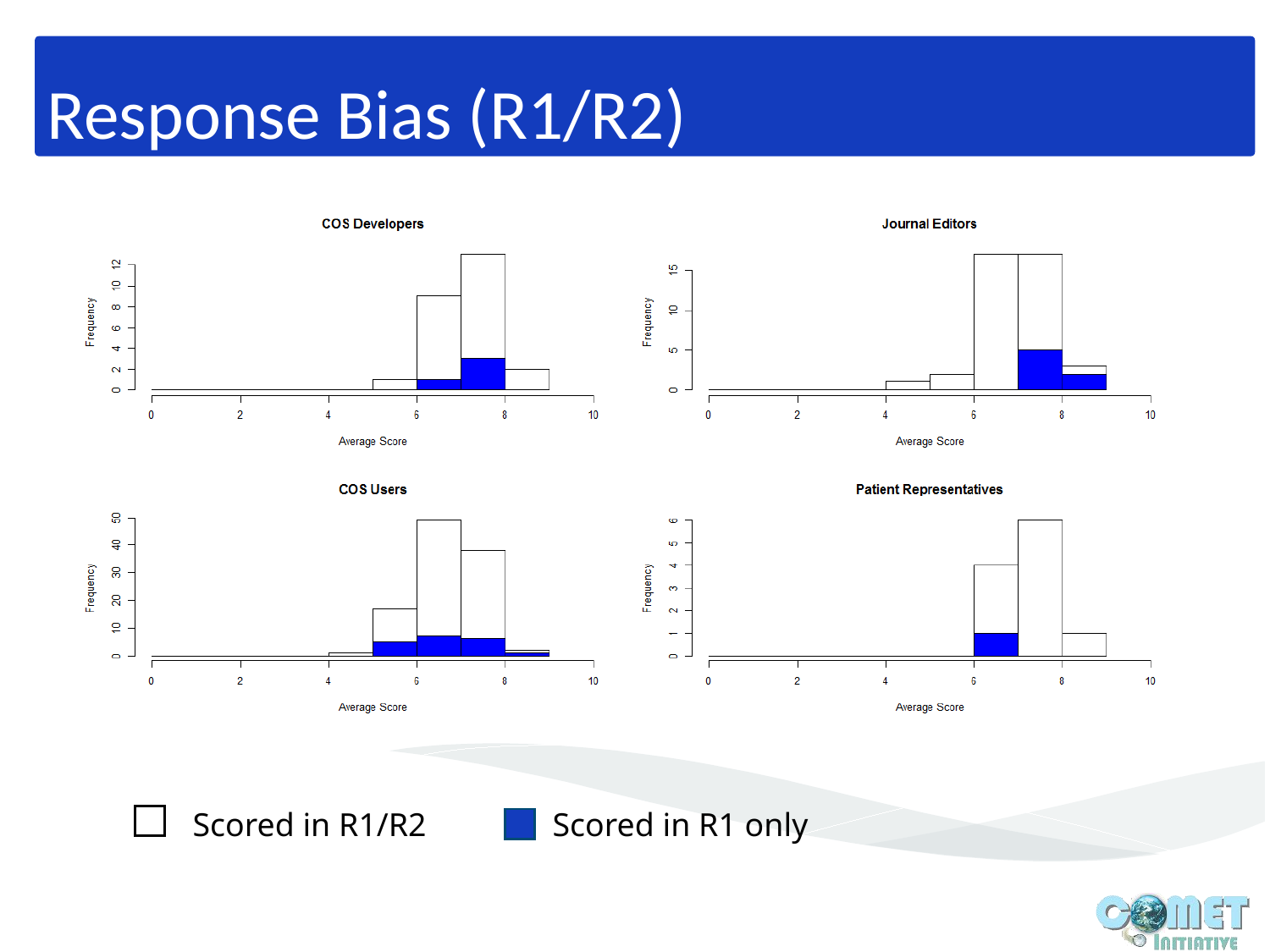

# Response Bias (R1/R2)
Scored in R1/R2
Scored in R1 only

## Slide 9
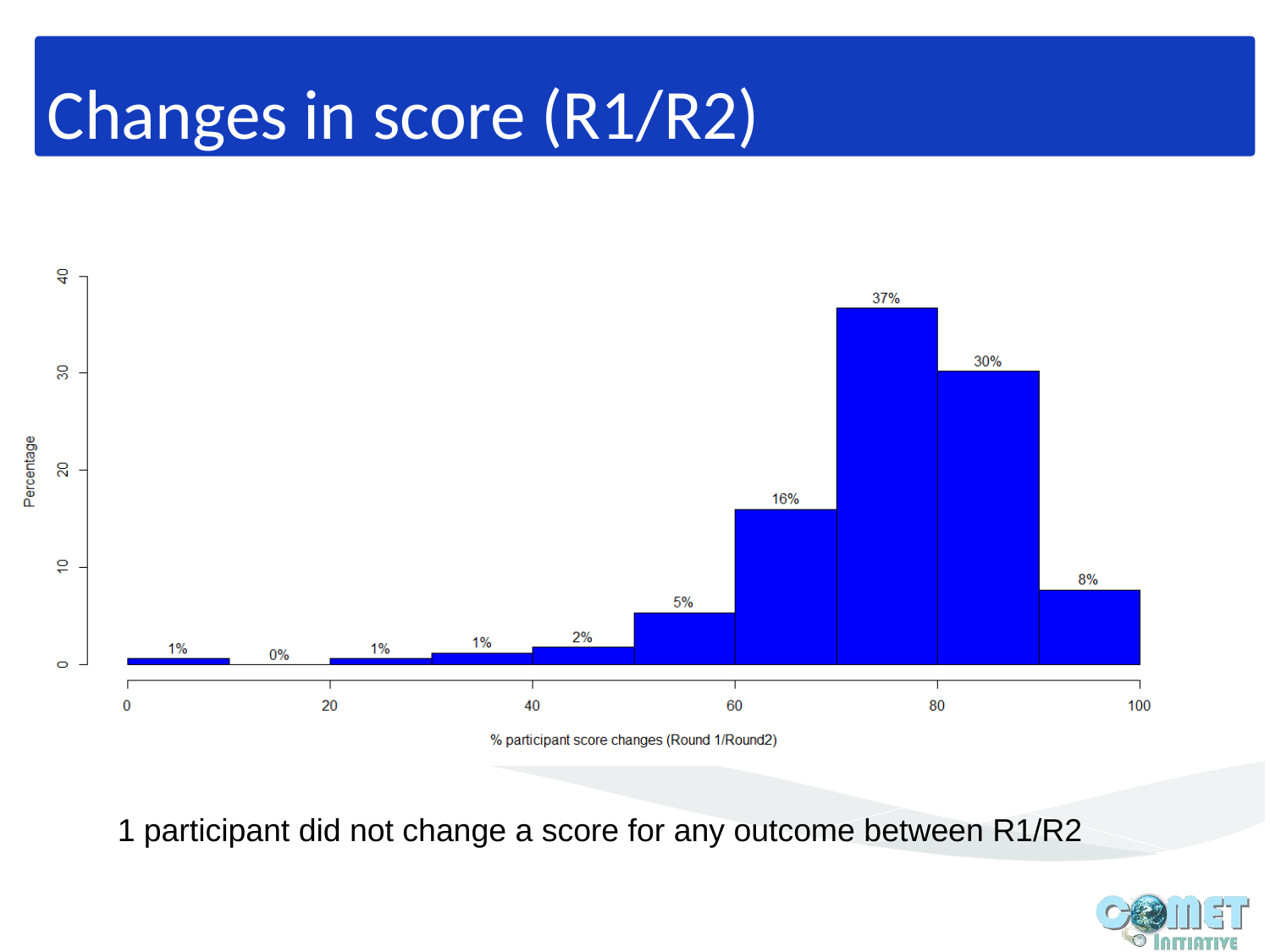

# Changes in score (R1/R2)
1 participant did not change a score for any outcome between R1/R2

## Slide 10
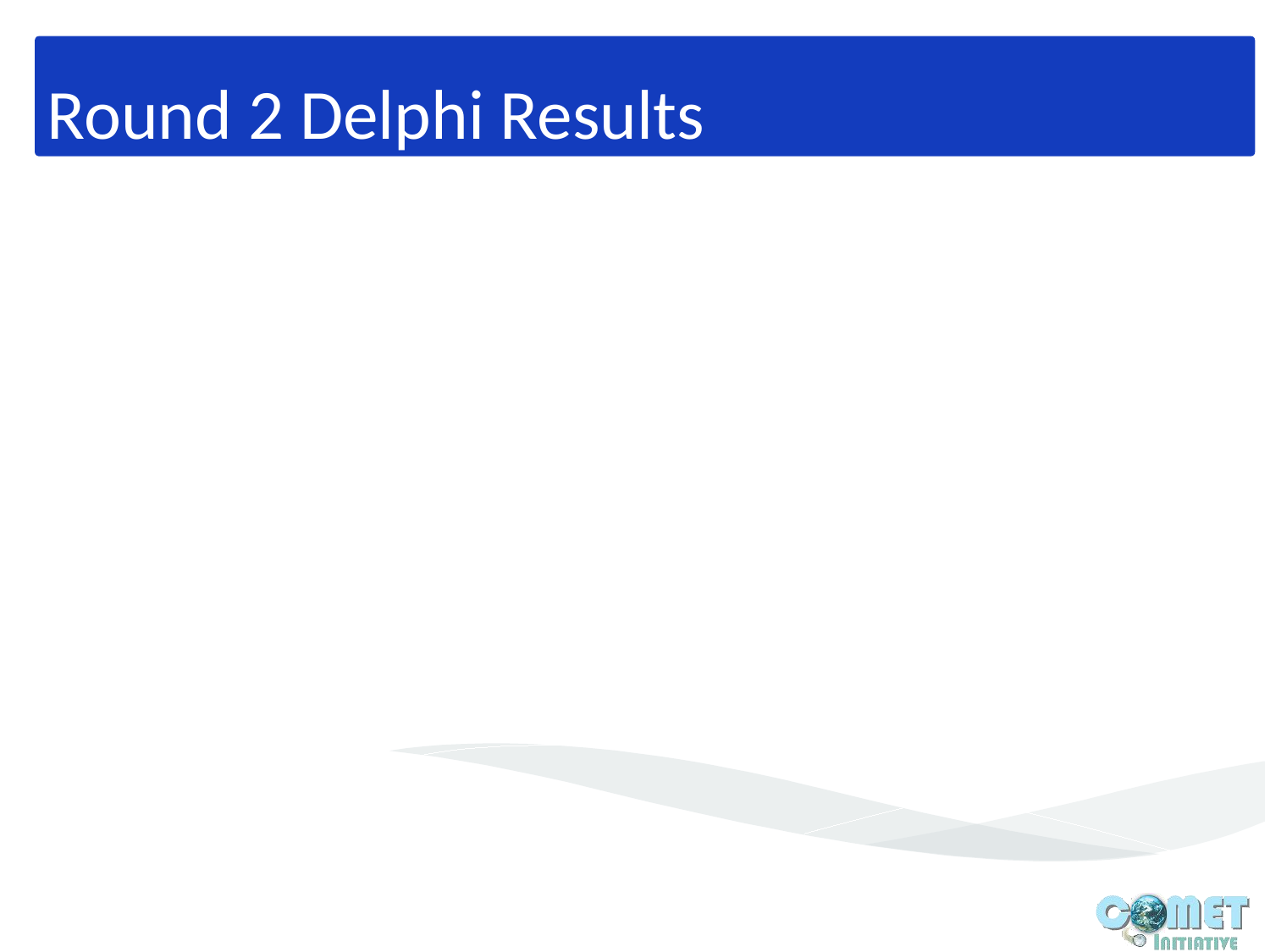

# Round 2 Delphi Results

## Slide 11
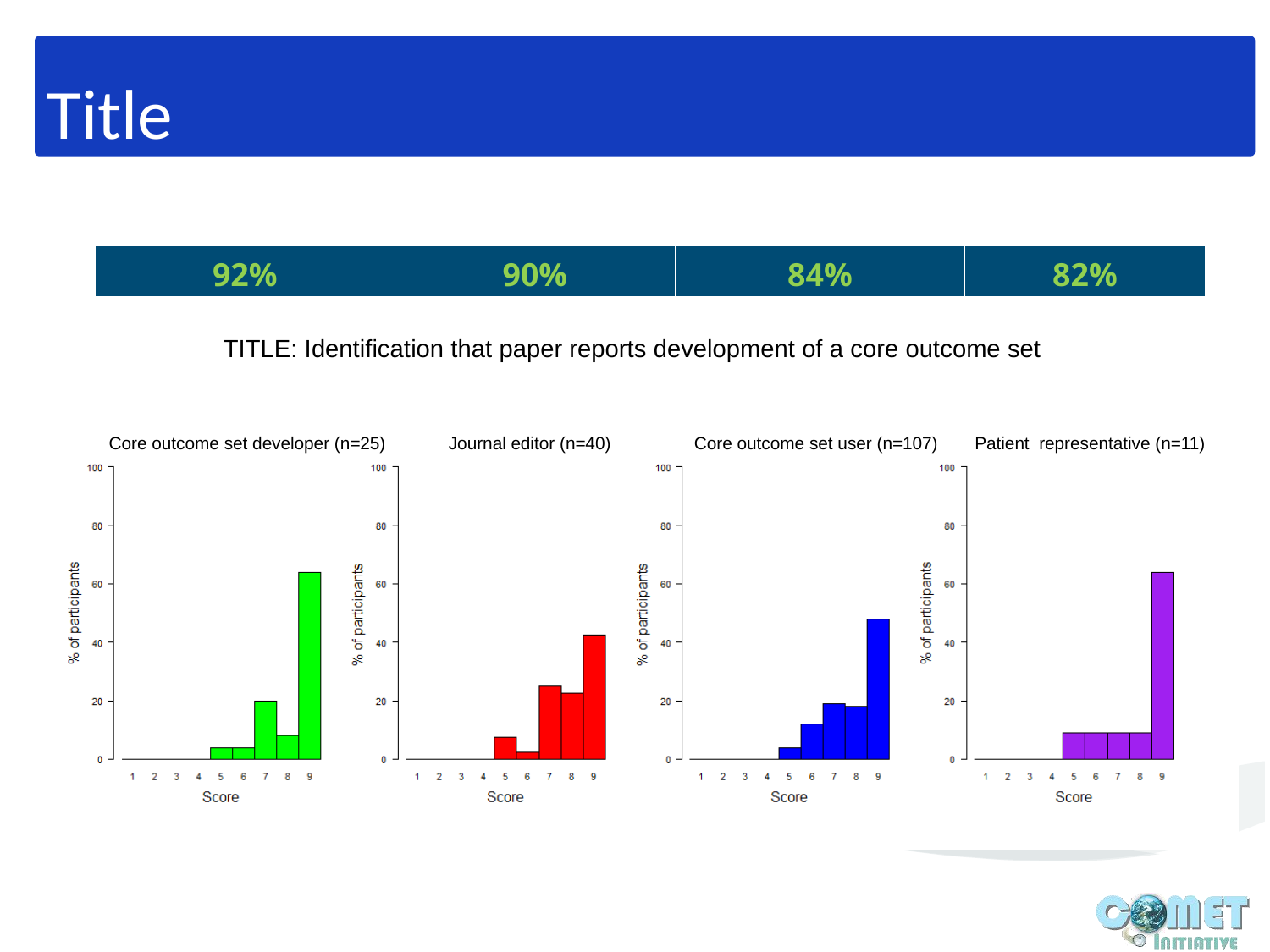

# Title
| 92% | 90% | 84% | 82% |
| --- | --- | --- | --- |
TITLE: Identification that paper reports development of a core outcome set
Core outcome set developer (n=25)
Journal editor (n=40)
Core outcome set user (n=107)
Patient representative (n=11)

## Slide 12
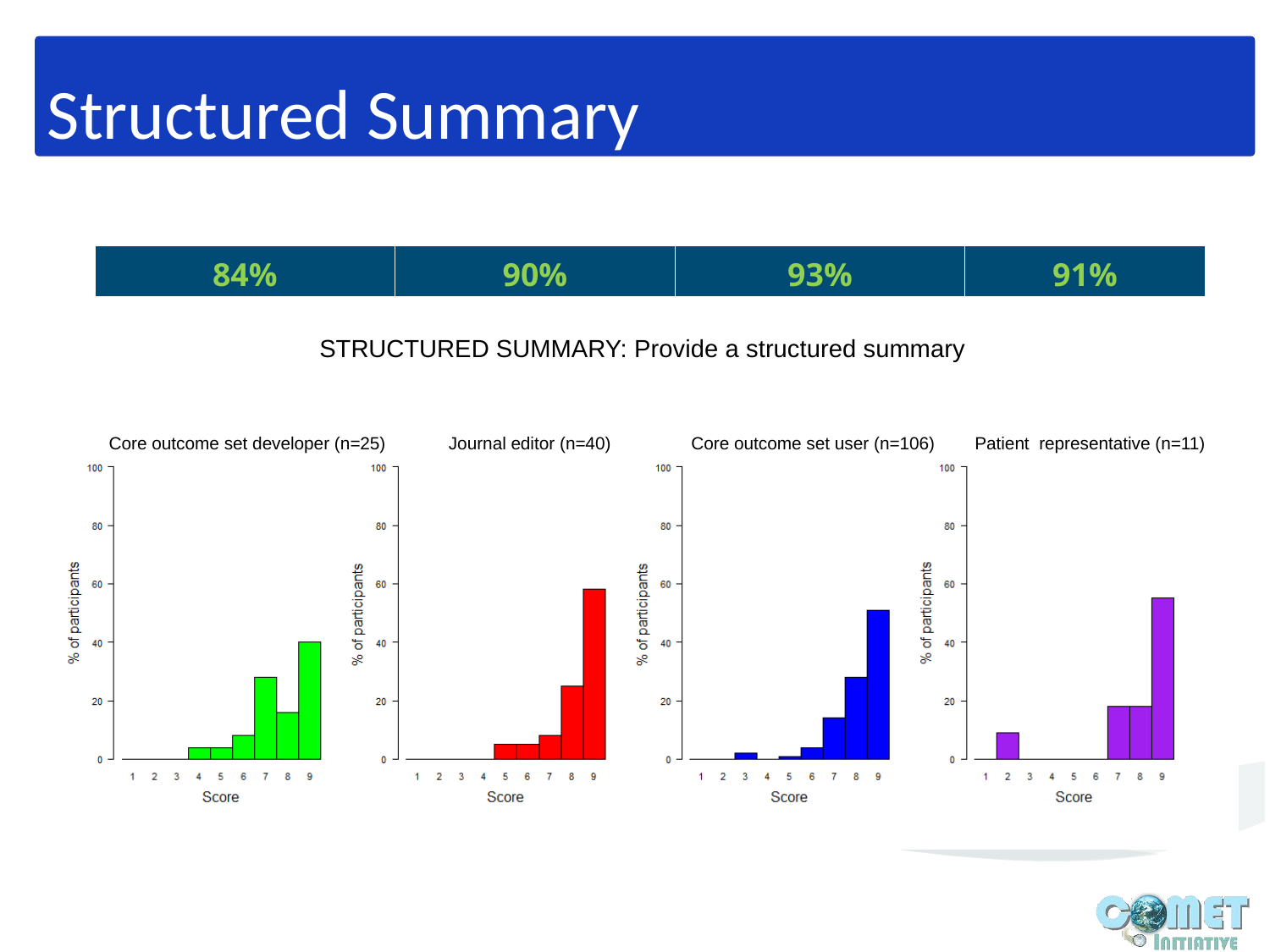

# Structured Summary
| 84% | 90% | 93% | 91% |
| --- | --- | --- | --- |
STRUCTURED SUMMARY: Provide a structured summary
Core outcome set developer (n=25)
Journal editor (n=40)
Core outcome set user (n=106)
Patient representative (n=11)

## Slide 13
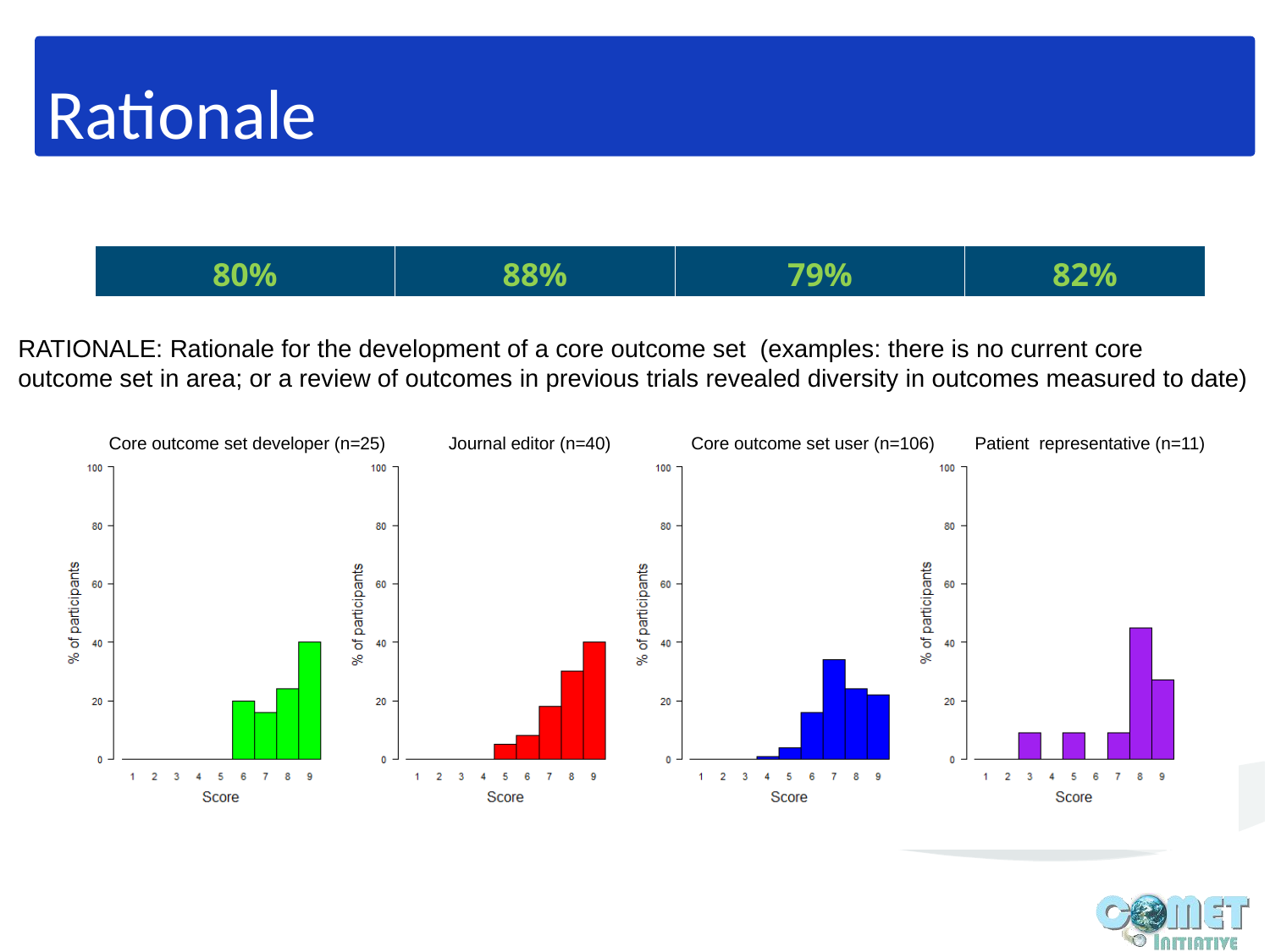

# Rationale
| 80% | 88% | 79% | 82% |
| --- | --- | --- | --- |
RATIONALE: Rationale for the development of a core outcome set (examples: there is no current core
outcome set in area; or a review of outcomes in previous trials revealed diversity in outcomes measured to date)
Core outcome set developer (n=25)
Journal editor (n=40)
Core outcome set user (n=106)
Patient representative (n=11)

## Slide 14
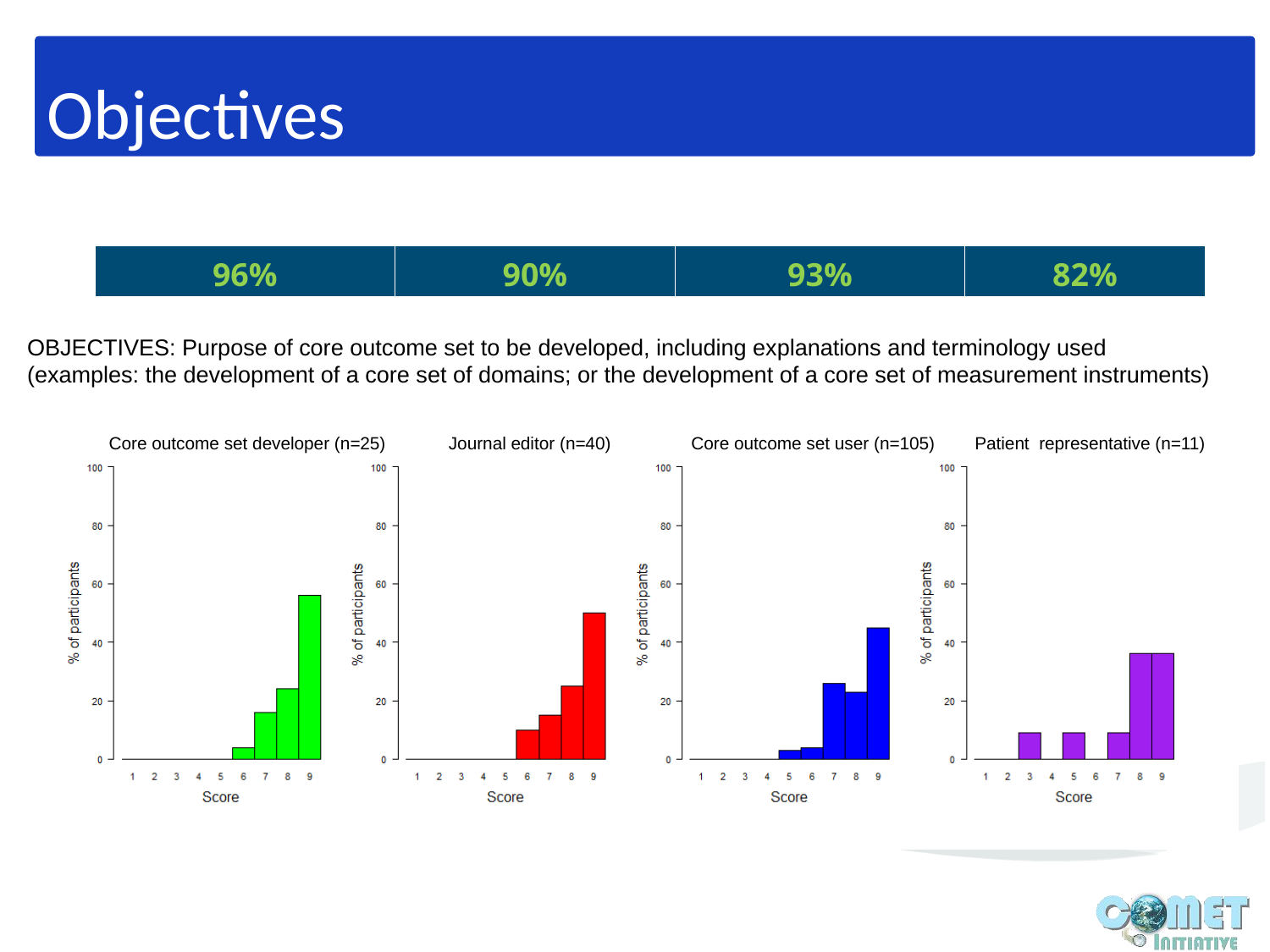

# Objectives
| 96% | 90% | 93% | 82% |
| --- | --- | --- | --- |
OBJECTIVES: Purpose of core outcome set to be developed, including explanations and terminology used
(examples: the development of a core set of domains; or the development of a core set of measurement instruments)
Core outcome set developer (n=25)
Journal editor (n=40)
Core outcome set user (n=105)
Patient representative (n=11)

## Slide 15
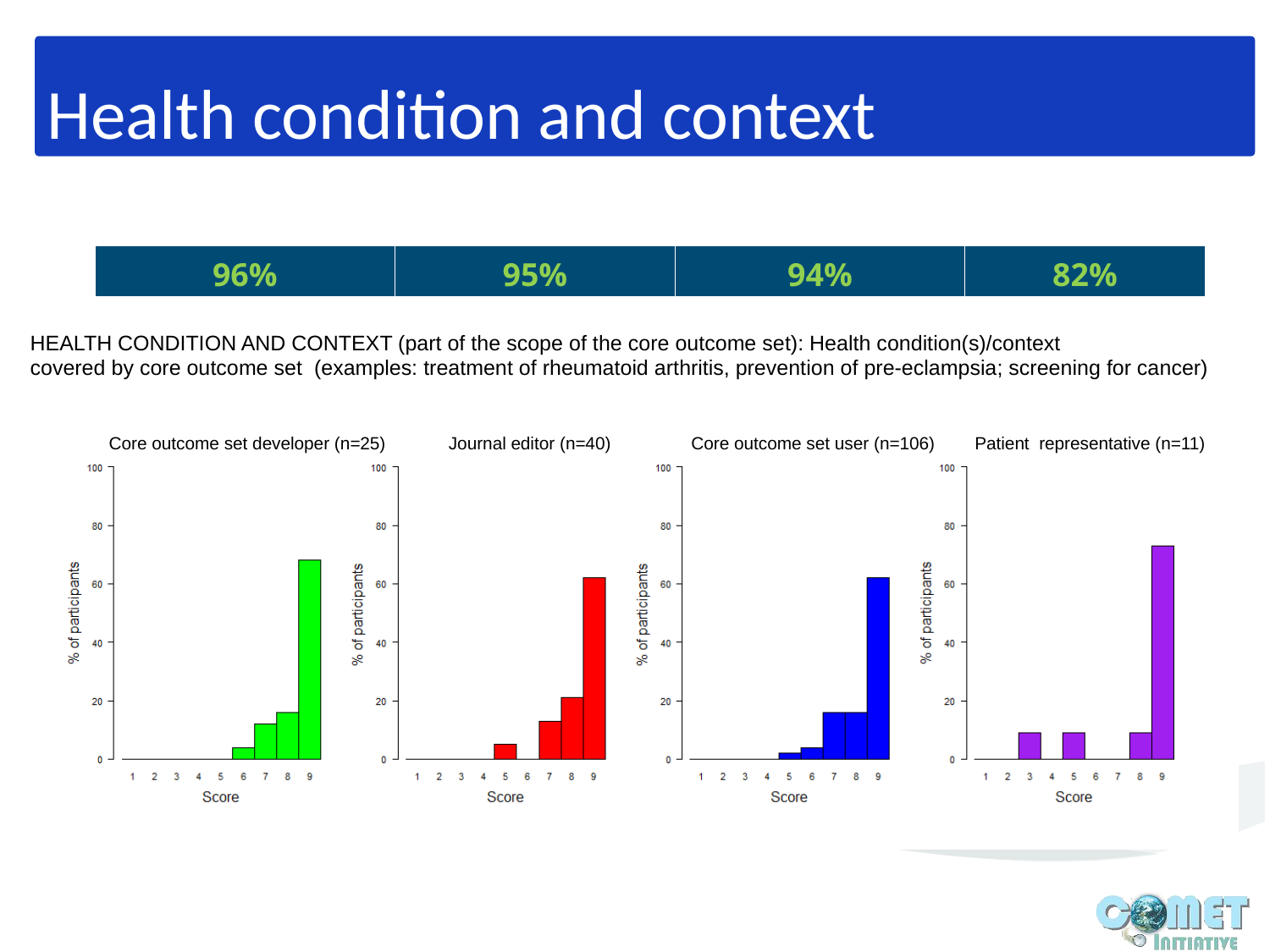

# Health condition and context
| 96% | 95% | 94% | 82% |
| --- | --- | --- | --- |
HEALTH CONDITION AND CONTEXT (part of the scope of the core outcome set): Health condition(s)/context
covered by core outcome set (examples: treatment of rheumatoid arthritis, prevention of pre-eclampsia; screening for cancer)
Core outcome set developer (n=25)
Journal editor (n=40)
Core outcome set user (n=106)
Patient representative (n=11)

## Slide 16
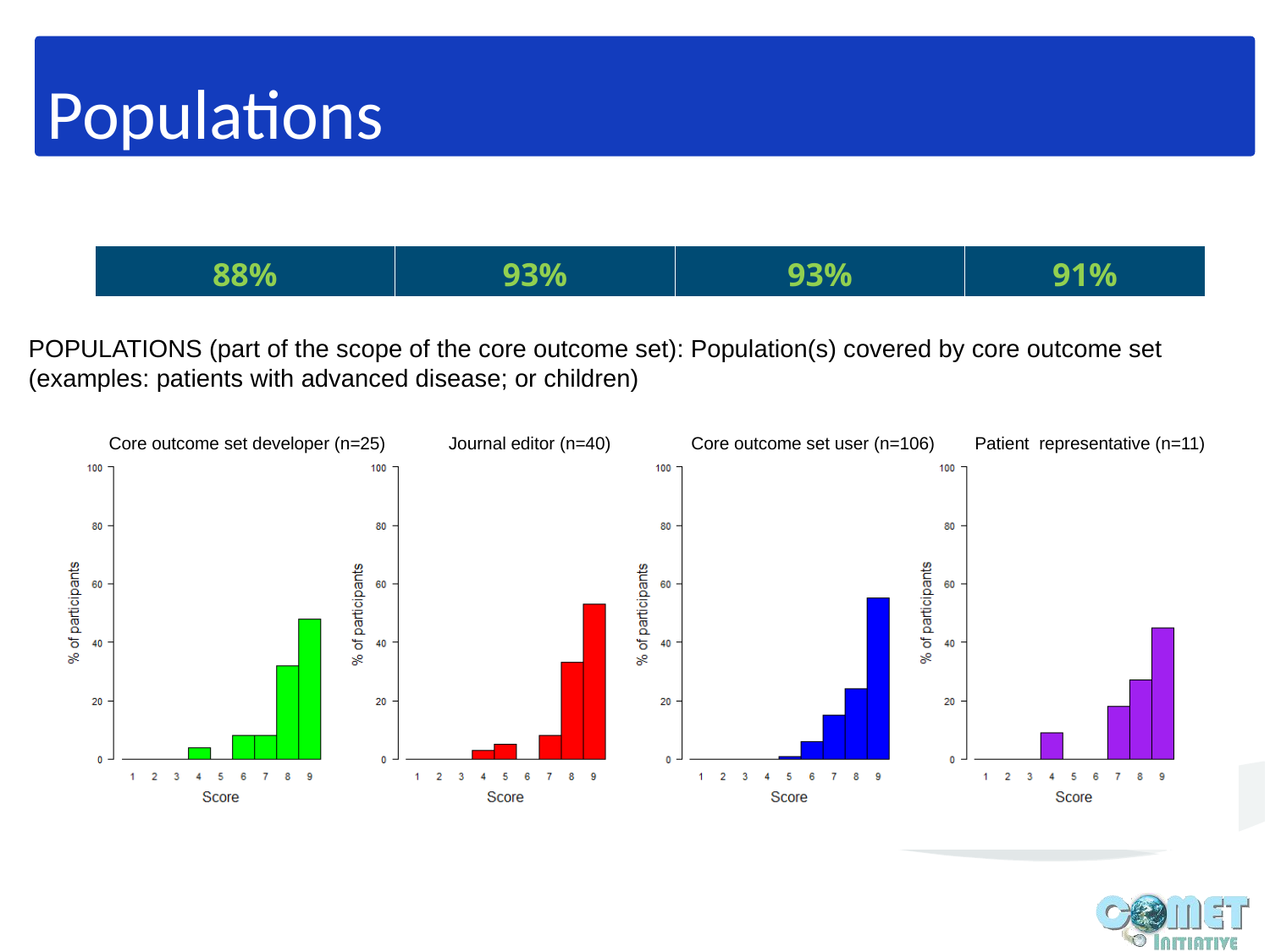

# Populations
| 88% | 93% | 93% | 91% |
| --- | --- | --- | --- |
POPULATIONS (part of the scope of the core outcome set): Population(s) covered by core outcome set
(examples: patients with advanced disease; or children)
Core outcome set developer (n=25)
Journal editor (n=40)
Core outcome set user (n=106)
Patient representative (n=11)

## Slide 17
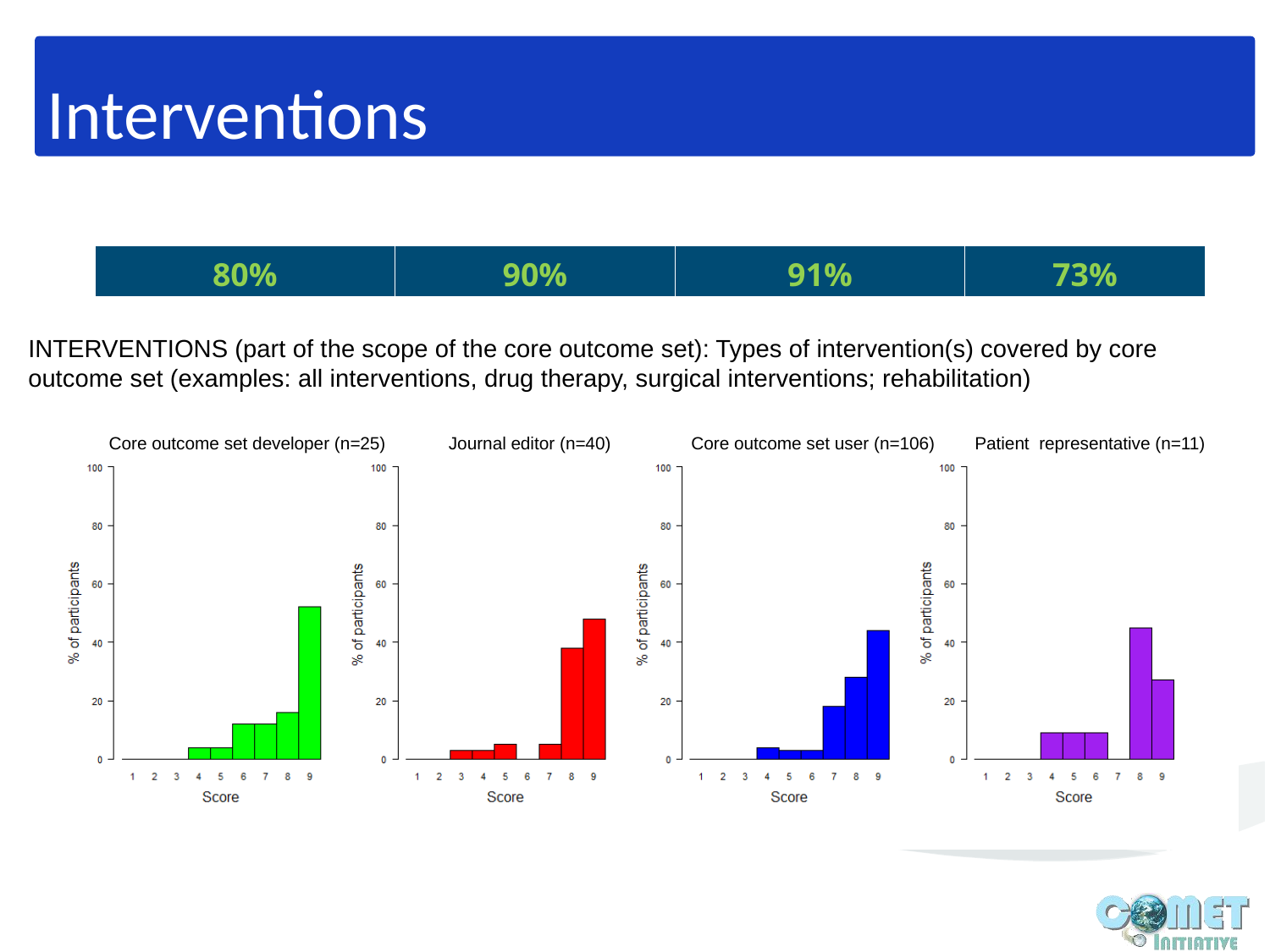

# Interventions
| 80% | 90% | 91% | 73% |
| --- | --- | --- | --- |
INTERVENTIONS (part of the scope of the core outcome set): Types of intervention(s) covered by core
outcome set (examples: all interventions, drug therapy, surgical interventions; rehabilitation)
Core outcome set developer (n=25)
Journal editor (n=40)
Core outcome set user (n=106)
Patient representative (n=11)

## Slide 18
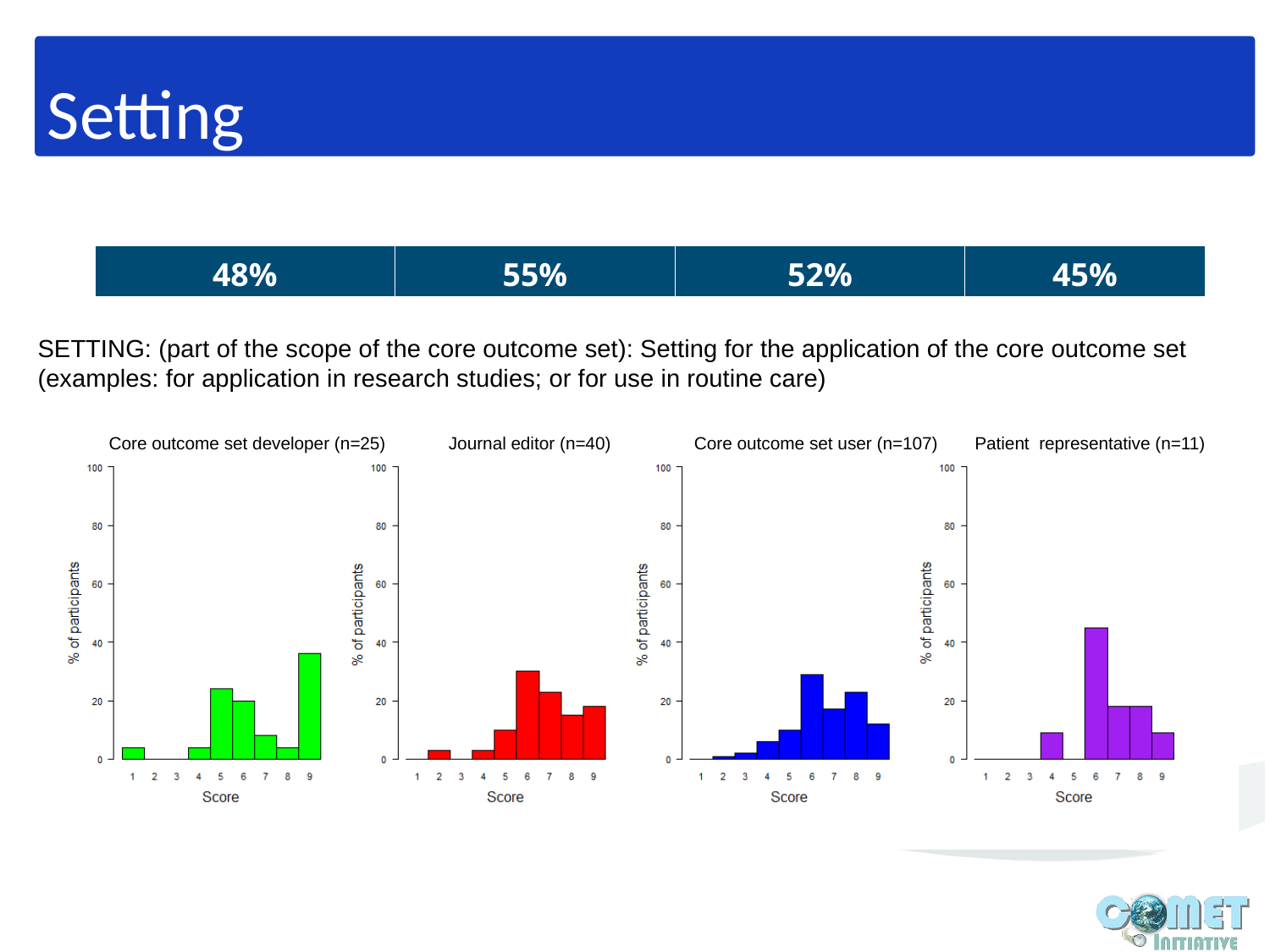

# Setting
| 48% | 55% | 52% | 45% |
| --- | --- | --- | --- |
SETTING: (part of the scope of the core outcome set): Setting for the application of the core outcome set
(examples: for application in research studies; or for use in routine care)
Core outcome set developer (n=25)
Journal editor (n=40)
Core outcome set user (n=107)
Patient representative (n=11)

## Slide 19
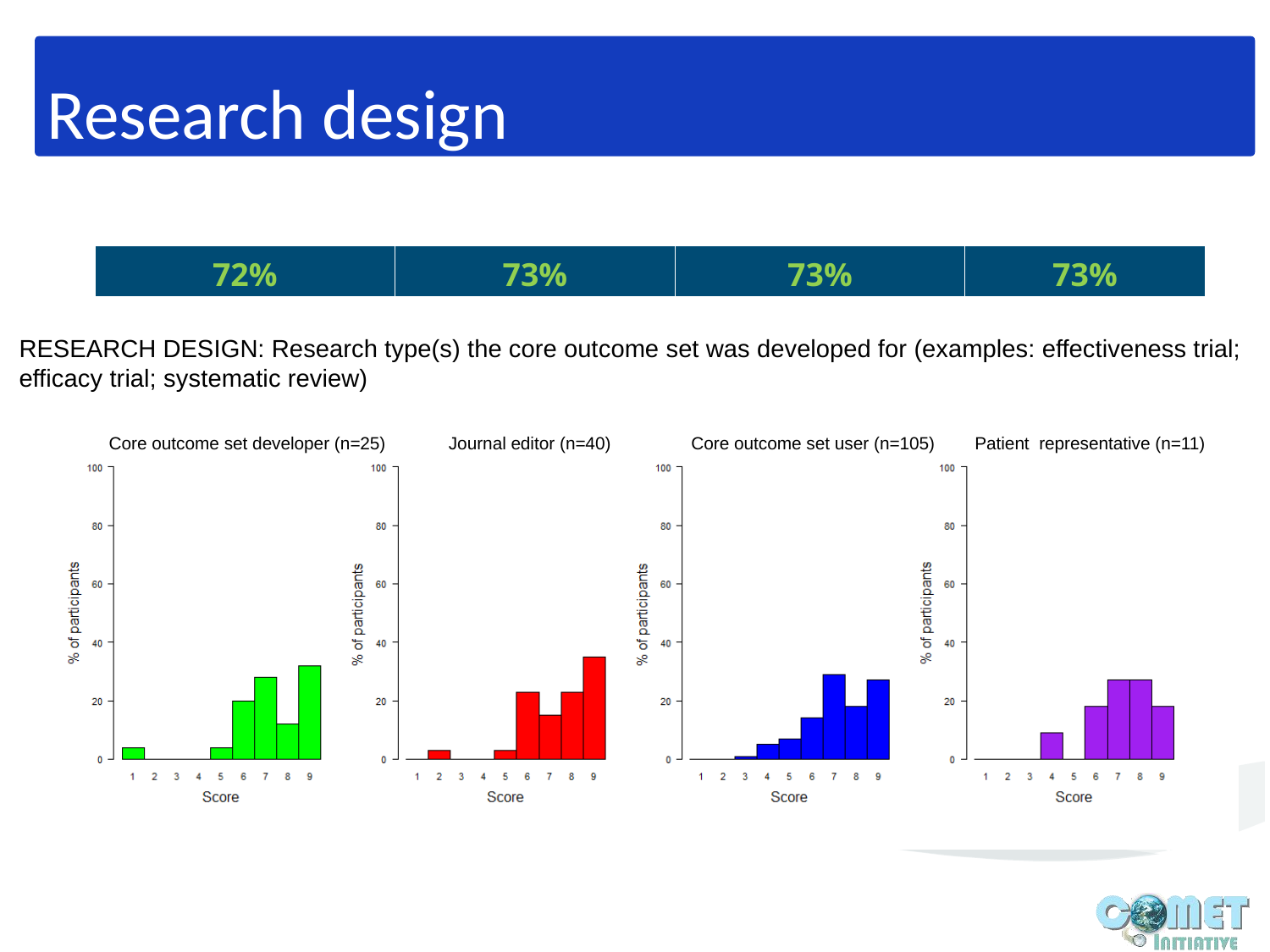

# Research design
| 72% | 73% | 73% | 73% |
| --- | --- | --- | --- |
RESEARCH DESIGN: Research type(s) the core outcome set was developed for (examples: effectiveness trial;
efficacy trial; systematic review)
Core outcome set developer (n=25)
Journal editor (n=40)
Core outcome set user (n=105)
Patient representative (n=11)

## Slide 20
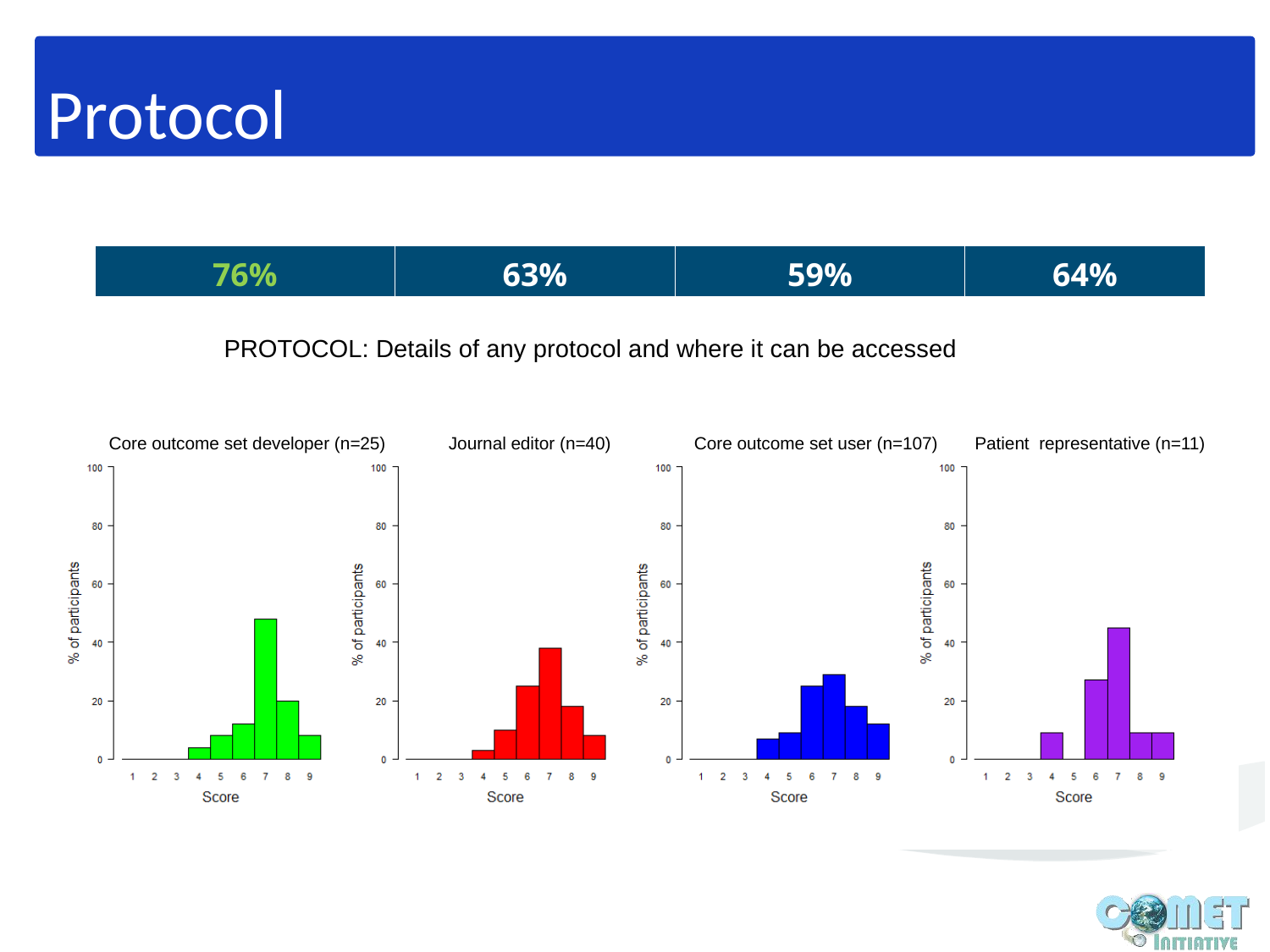

# Protocol
| 76% | 63% | 59% | 64% |
| --- | --- | --- | --- |
PROTOCOL: Details of any protocol and where it can be accessed
Core outcome set developer (n=25)
Journal editor (n=40)
Core outcome set user (n=107)
Patient representative (n=11)

## Slide 21
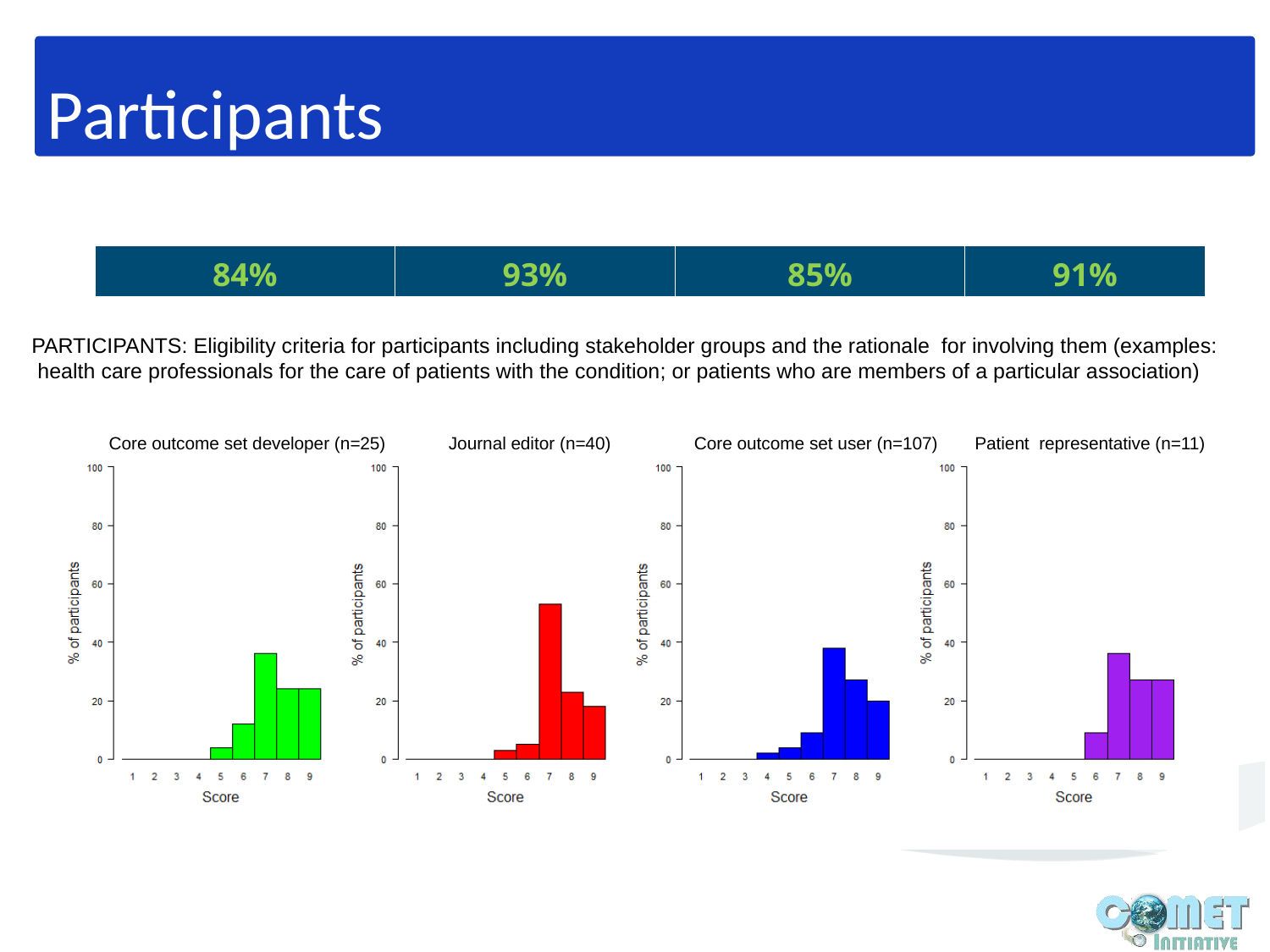

# Participants
| 84% | 93% | 85% | 91% |
| --- | --- | --- | --- |
PARTICIPANTS: Eligibility criteria for participants including stakeholder groups and the rationale for involving them (examples:
 health care professionals for the care of patients with the condition; or patients who are members of a particular association)
Core outcome set developer (n=25)
Journal editor (n=40)
Core outcome set user (n=107)
Patient representative (n=11)

## Slide 22
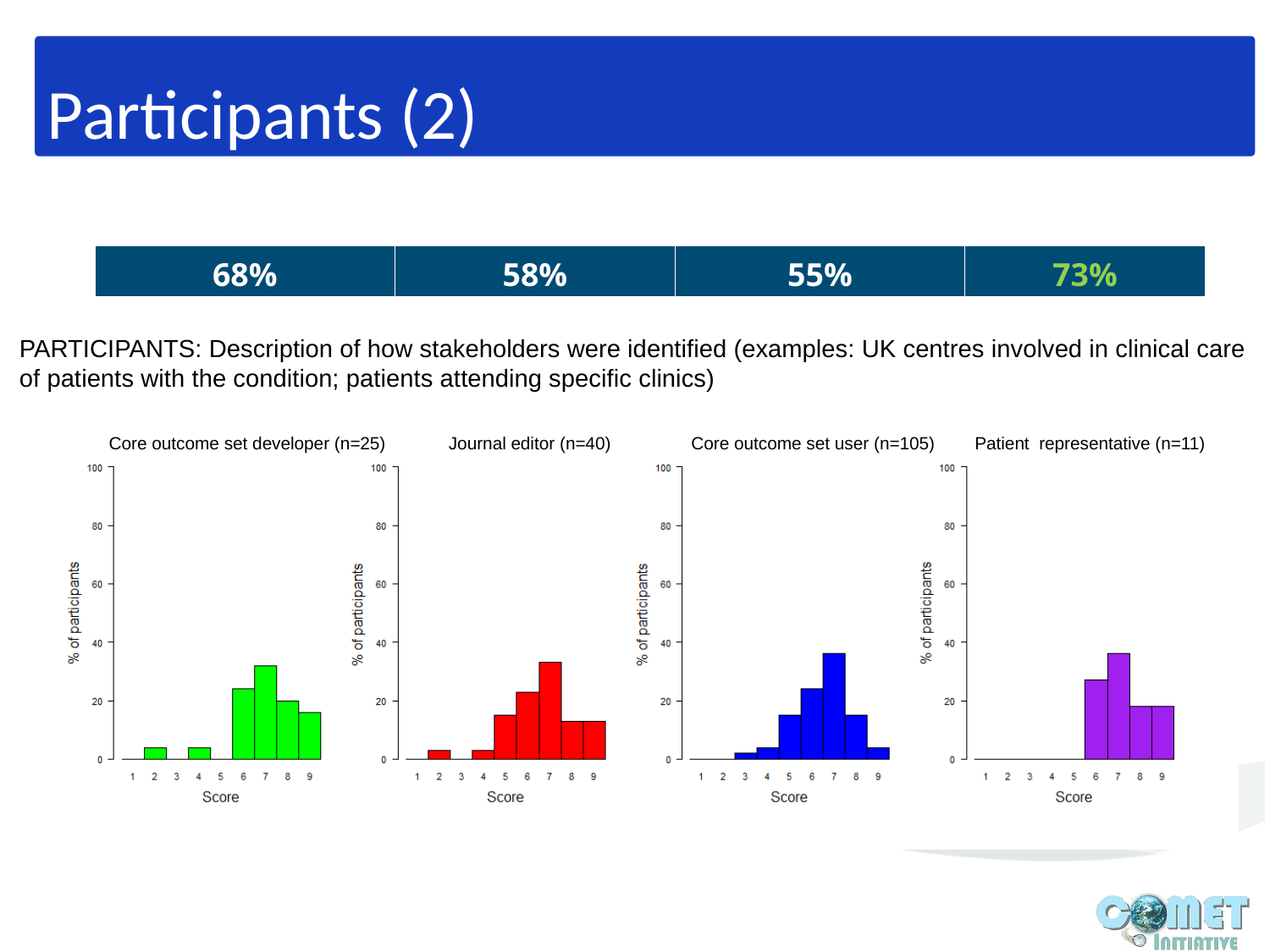

# Participants (2)
| 68% | 58% | 55% | 73% |
| --- | --- | --- | --- |
PARTICIPANTS: Description of how stakeholders were identified (examples: UK centres involved in clinical care
of patients with the condition; patients attending specific clinics)
Core outcome set developer (n=25)
Journal editor (n=40)
Core outcome set user (n=105)
Patient representative (n=11)

## Slide 23
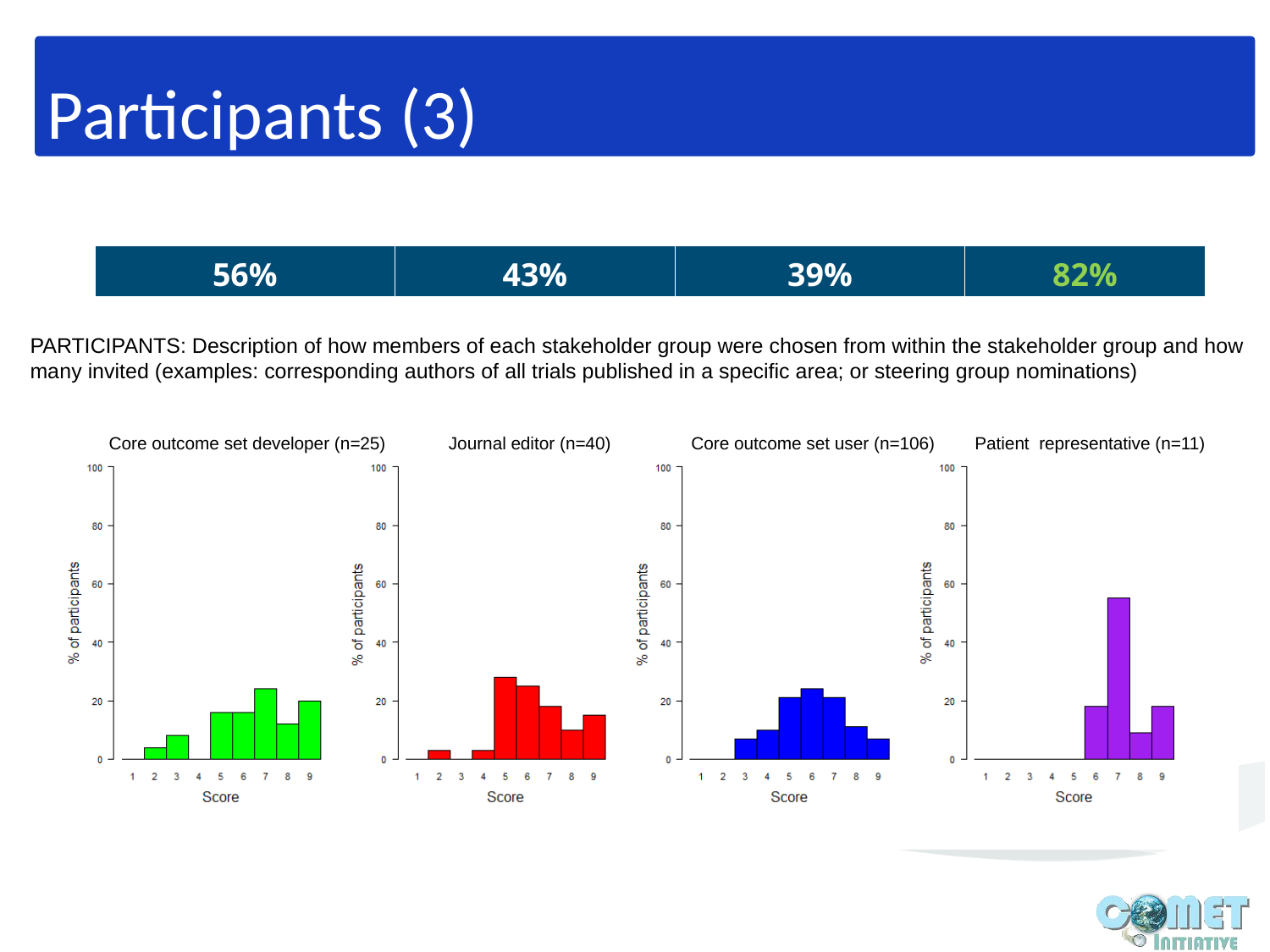

# Participants (3)
| 56% | 43% | 39% | 82% |
| --- | --- | --- | --- |
PARTICIPANTS: Description of how members of each stakeholder group were chosen from within the stakeholder group and how
many invited (examples: corresponding authors of all trials published in a specific area; or steering group nominations)
Core outcome set developer (n=25)
Journal editor (n=40)
Core outcome set user (n=106)
Patient representative (n=11)

## Slide 24
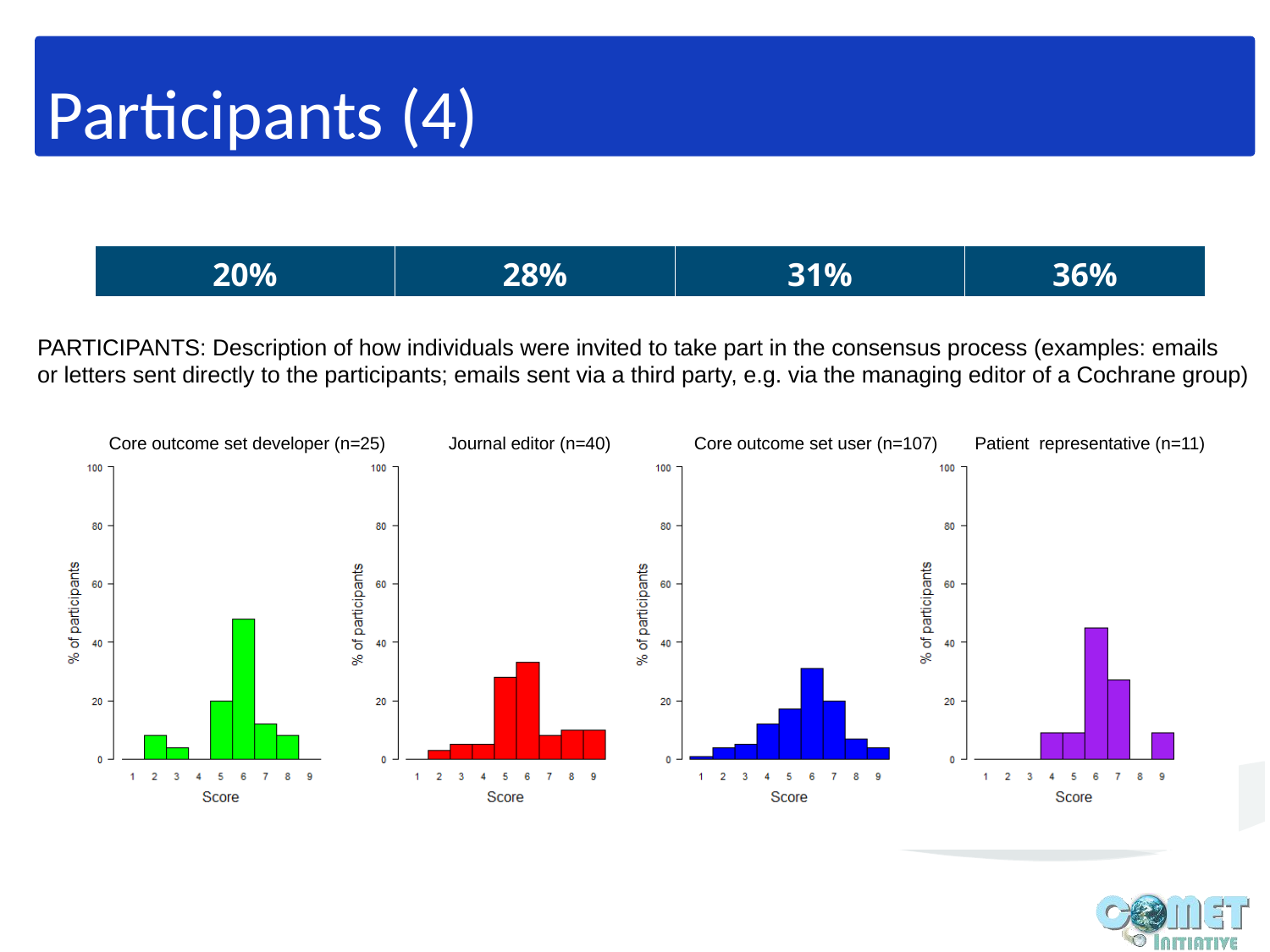

# Participants (4)
| 20% | 28% | 31% | 36% |
| --- | --- | --- | --- |
PARTICIPANTS: Description of how individuals were invited to take part in the consensus process (examples: emails
or letters sent directly to the participants; emails sent via a third party, e.g. via the managing editor of a Cochrane group)
Core outcome set developer (n=25)
Journal editor (n=40)
Core outcome set user (n=107)
Patient representative (n=11)

## Slide 25
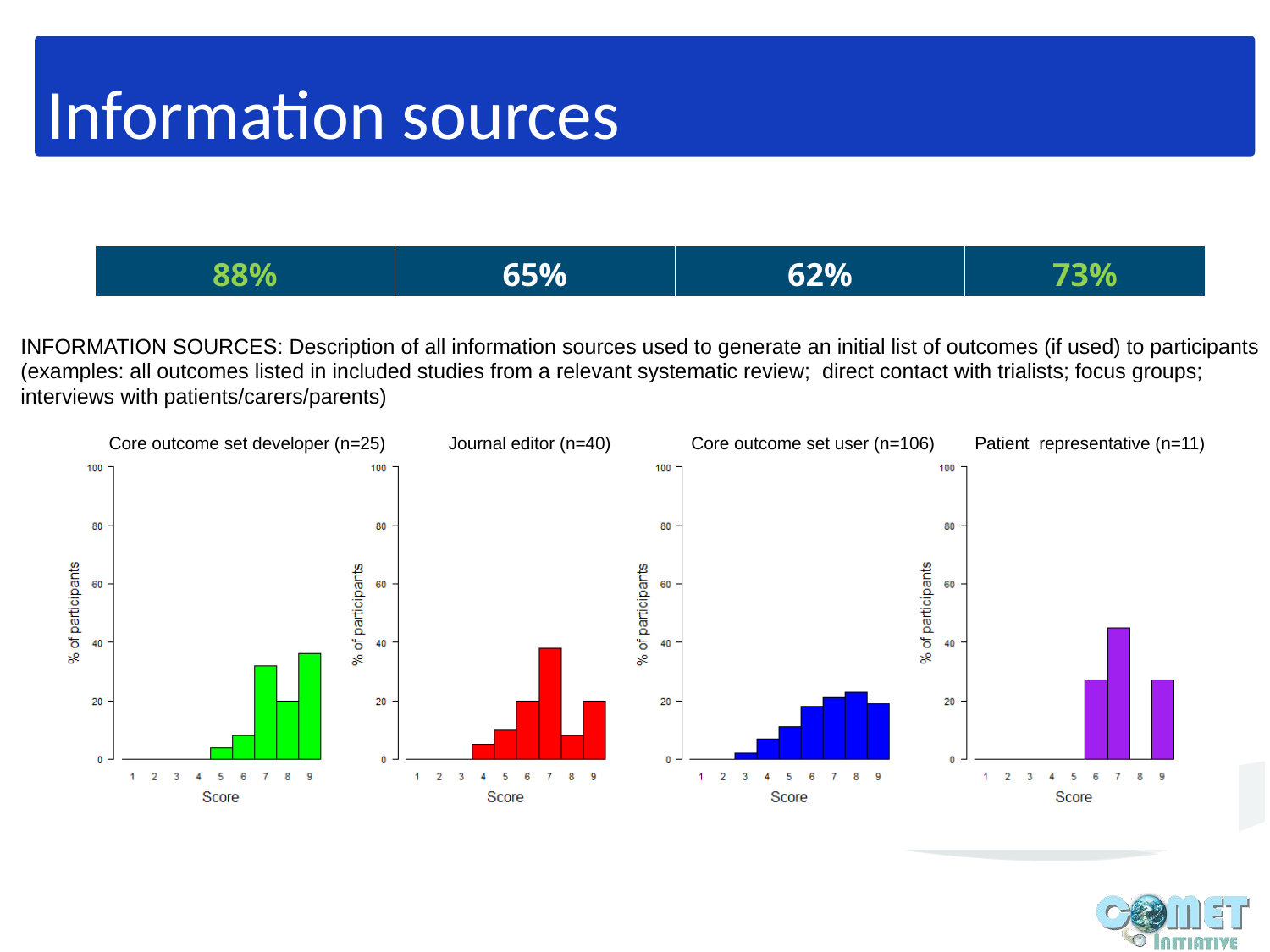

# Information sources
| 88% | 65% | 62% | 73% |
| --- | --- | --- | --- |
INFORMATION SOURCES: Description of all information sources used to generate an initial list of outcomes (if used) to participants
(examples: all outcomes listed in included studies from a relevant systematic review; direct contact with trialists; focus groups;
interviews with patients/carers/parents)
Core outcome set developer (n=25)
Journal editor (n=40)
Core outcome set user (n=106)
Patient representative (n=11)

## Slide 26
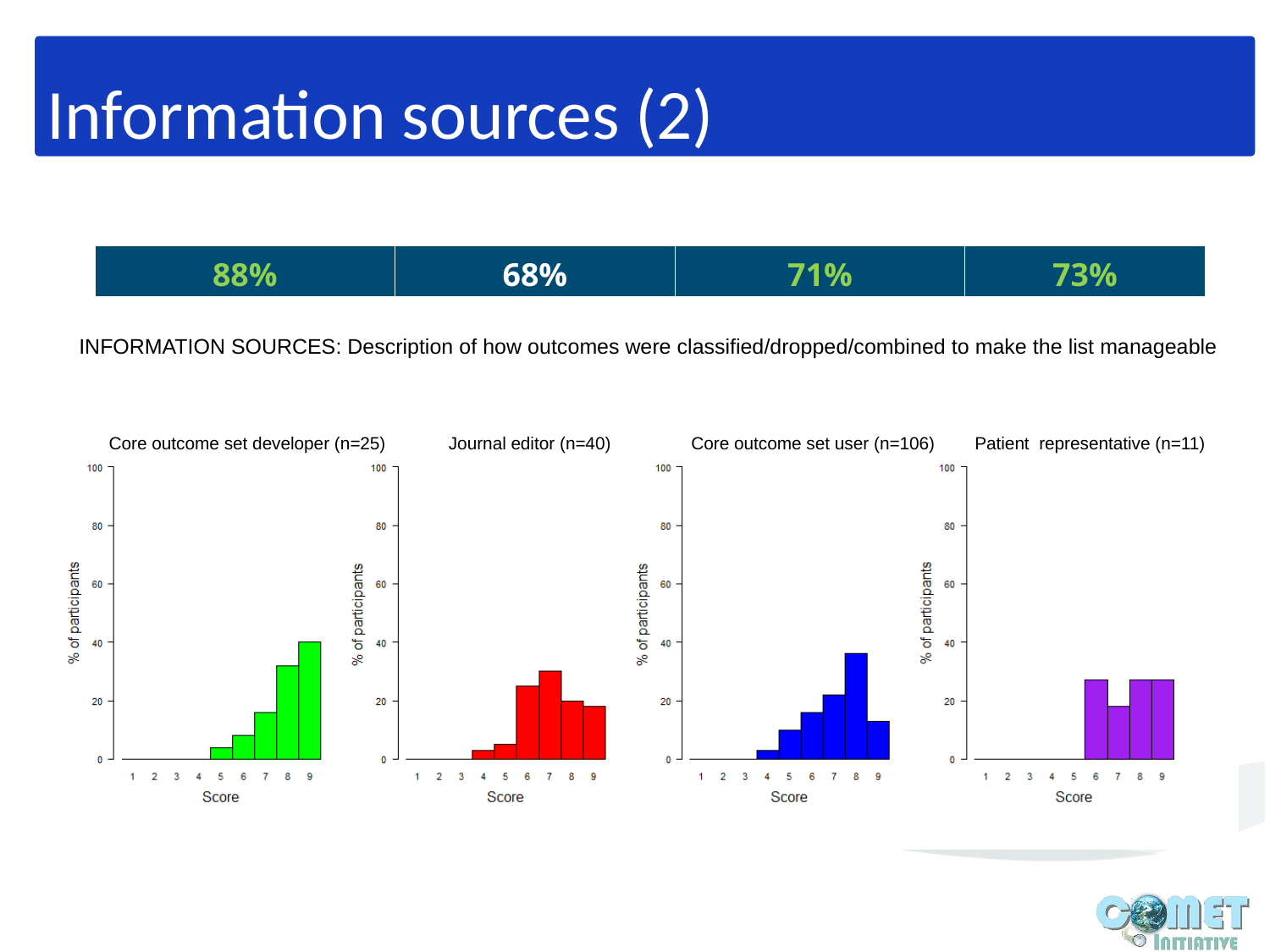

# Information sources (2)
| 88% | 68% | 71% | 73% |
| --- | --- | --- | --- |
INFORMATION SOURCES: Description of how outcomes were classified/dropped/combined to make the list manageable
Core outcome set developer (n=25)
Journal editor (n=40)
Core outcome set user (n=106)
Patient representative (n=11)

## Slide 27
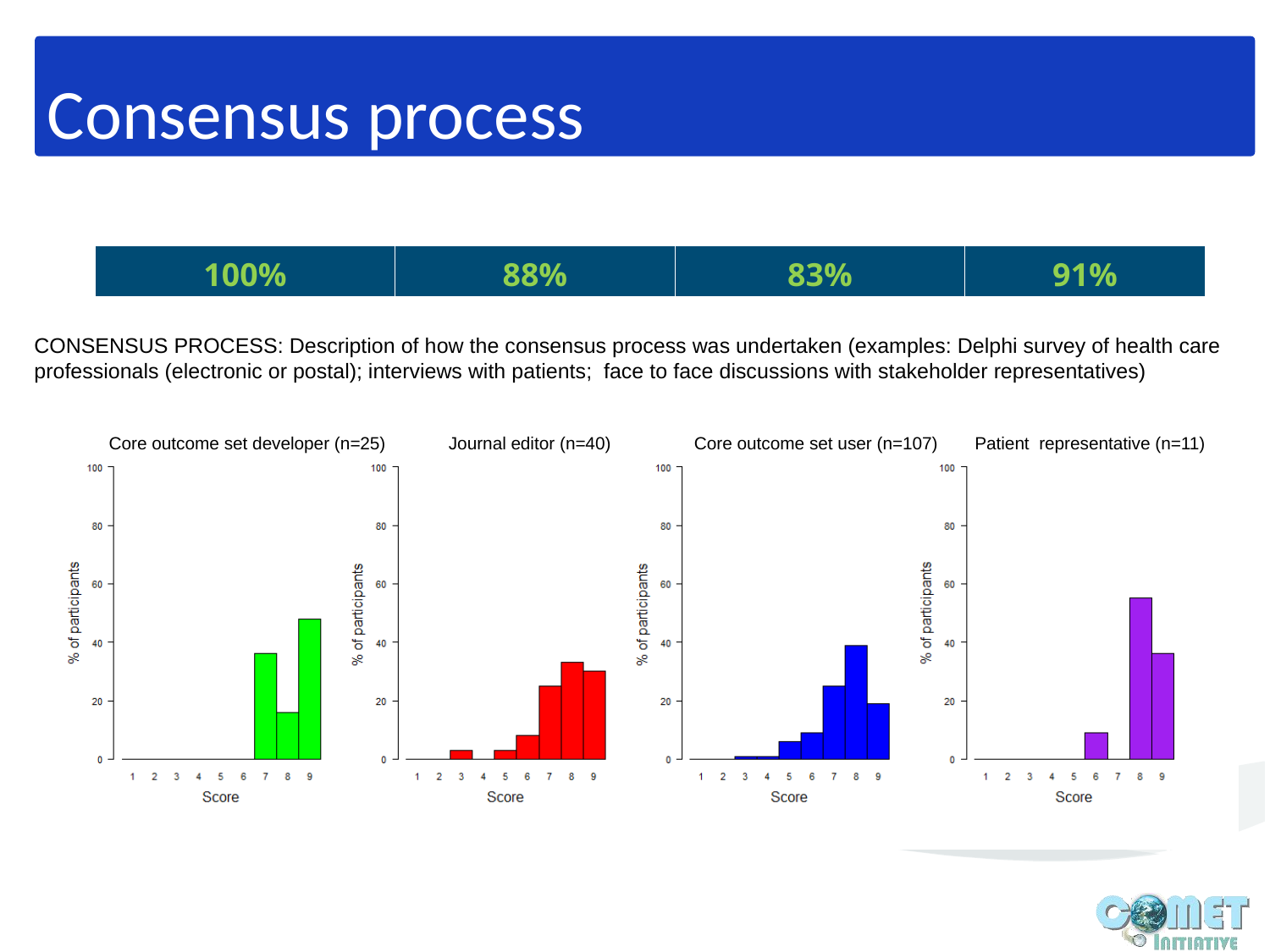

# Consensus process
| 100% | 88% | 83% | 91% |
| --- | --- | --- | --- |
CONSENSUS PROCESS: Description of how the consensus process was undertaken (examples: Delphi survey of health care
professionals (electronic or postal); interviews with patients; face to face discussions with stakeholder representatives)
Core outcome set developer (n=25)
Journal editor (n=40)
Core outcome set user (n=107)
Patient representative (n=11)

## Slide 28
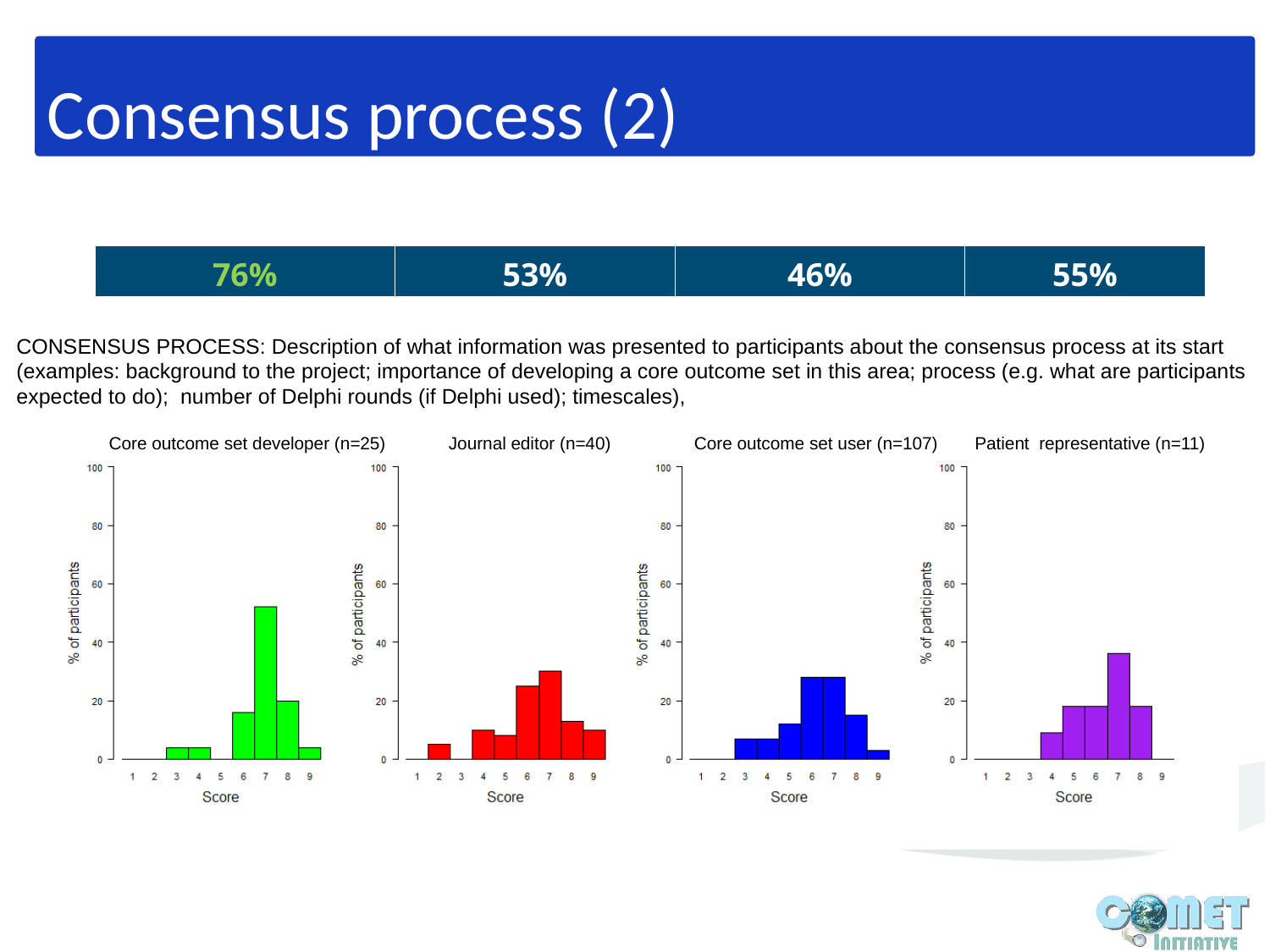

# Consensus process (2)
| 76% | 53% | 46% | 55% |
| --- | --- | --- | --- |
CONSENSUS PROCESS: Description of what information was presented to participants about the consensus process at its start
(examples: background to the project; importance of developing a core outcome set in this area; process (e.g. what are participants
expected to do); number of Delphi rounds (if Delphi used); timescales),
Core outcome set developer (n=25)
Journal editor (n=40)
Core outcome set user (n=107)
Patient representative (n=11)

## Slide 29
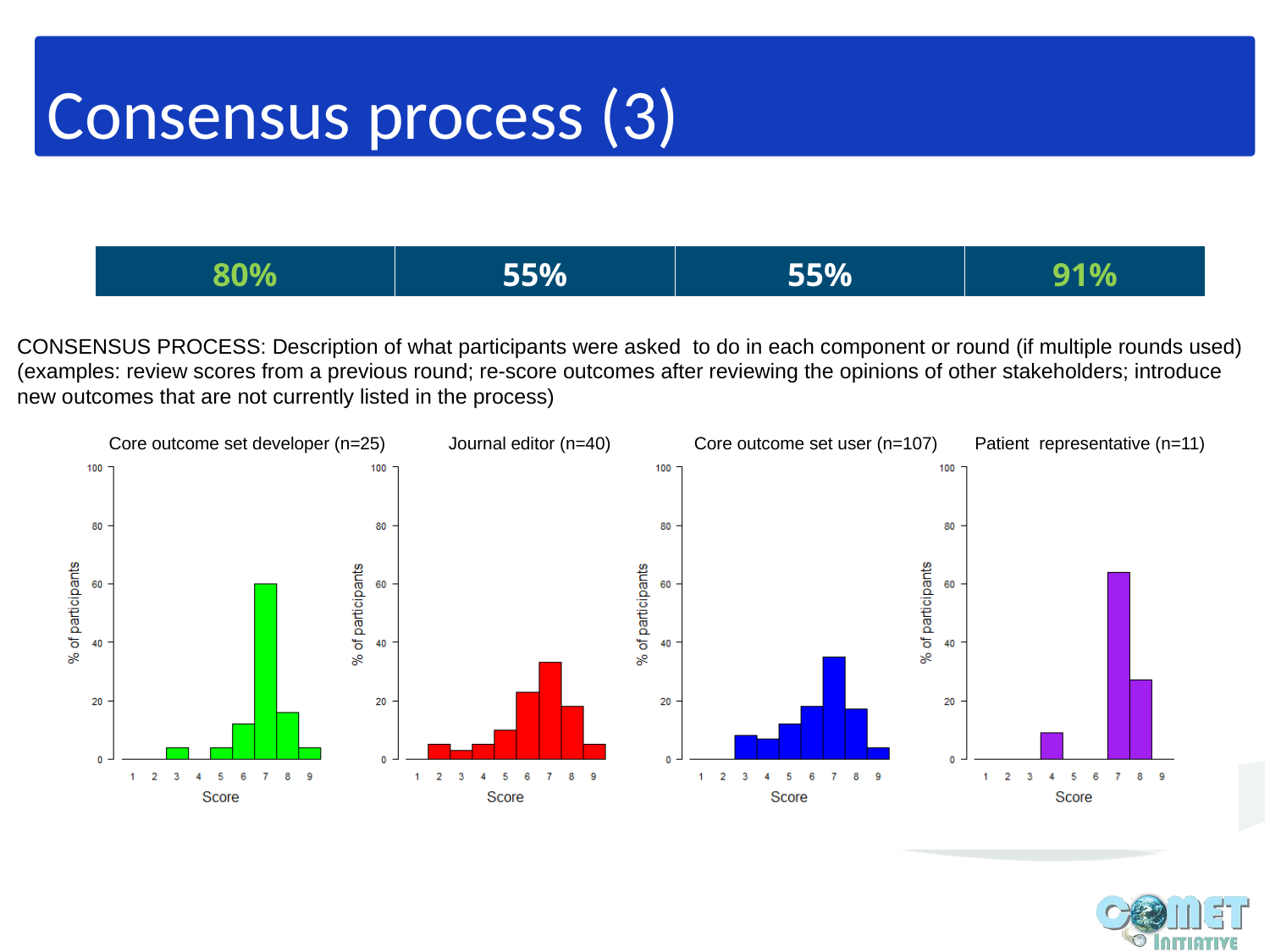

# Consensus process (3)
| 80% | 55% | 55% | 91% |
| --- | --- | --- | --- |
CONSENSUS PROCESS: Description of what participants were asked to do in each component or round (if multiple rounds used)
(examples: review scores from a previous round; re-score outcomes after reviewing the opinions of other stakeholders; introduce
new outcomes that are not currently listed in the process)
Core outcome set developer (n=25)
Journal editor (n=40)
Core outcome set user (n=107)
Patient representative (n=11)

## Slide 30
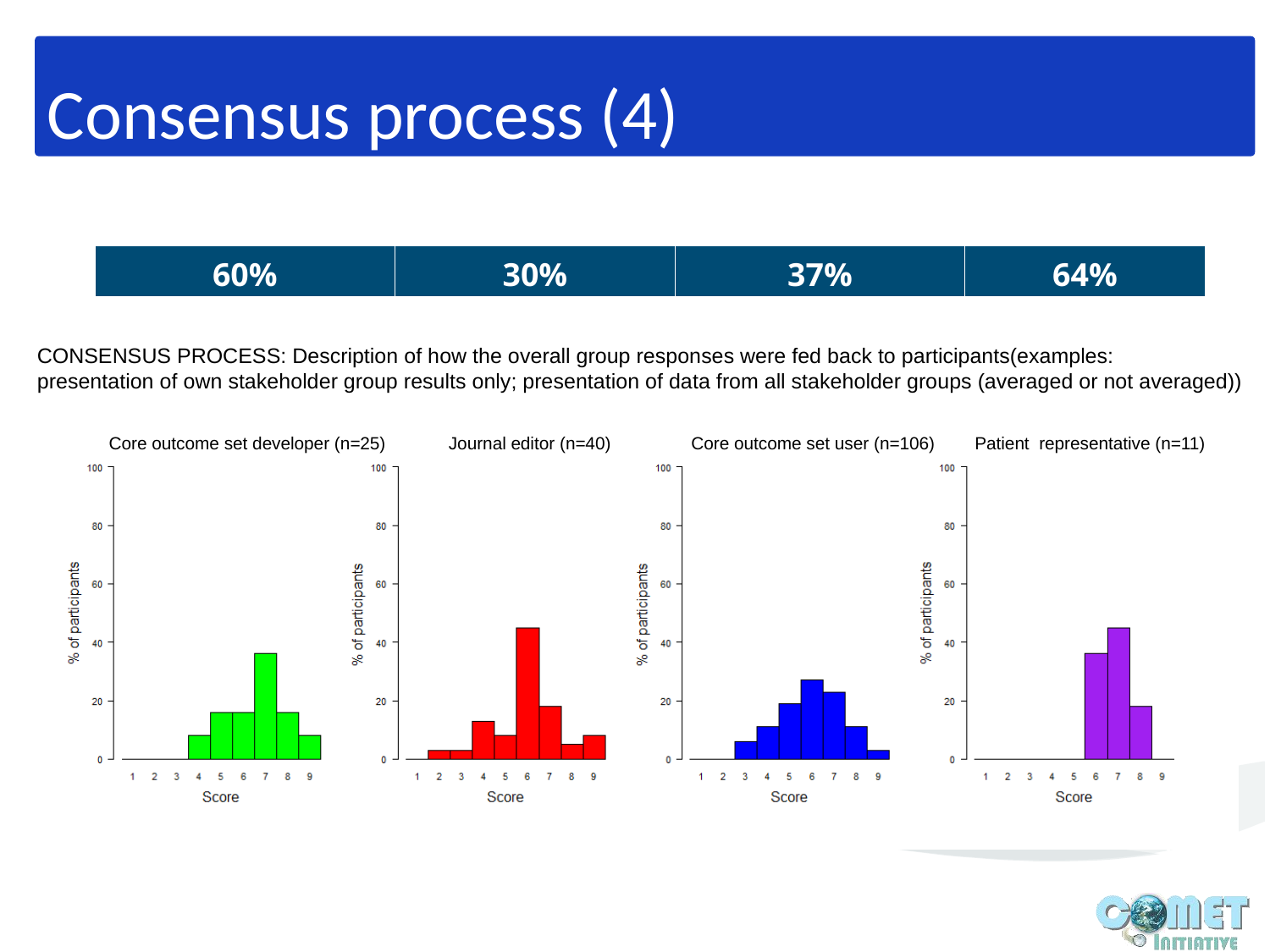

# Consensus process (4)
| 60% | 30% | 37% | 64% |
| --- | --- | --- | --- |
CONSENSUS PROCESS: Description of how the overall group responses were fed back to participants(examples:
presentation of own stakeholder group results only; presentation of data from all stakeholder groups (averaged or not averaged))
Core outcome set developer (n=25)
Journal editor (n=40)
Core outcome set user (n=106)
Patient representative (n=11)

## Slide 31
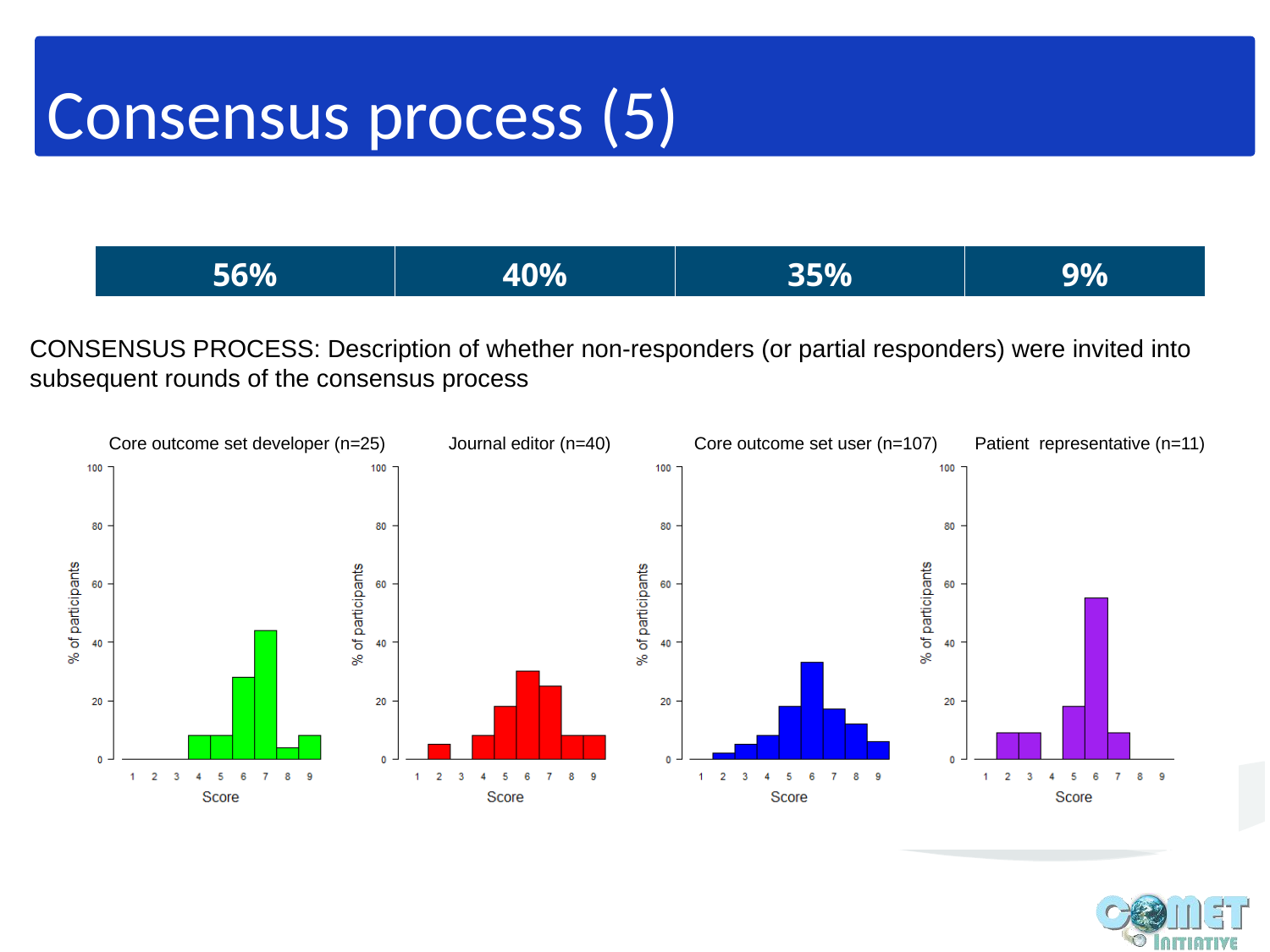

# Consensus process (5)
| 56% | 40% | 35% | 9% |
| --- | --- | --- | --- |
CONSENSUS PROCESS: Description of whether non-responders (or partial responders) were invited into
subsequent rounds of the consensus process
Core outcome set developer (n=25)
Journal editor (n=40)
Core outcome set user (n=107)
Patient representative (n=11)

## Slide 32
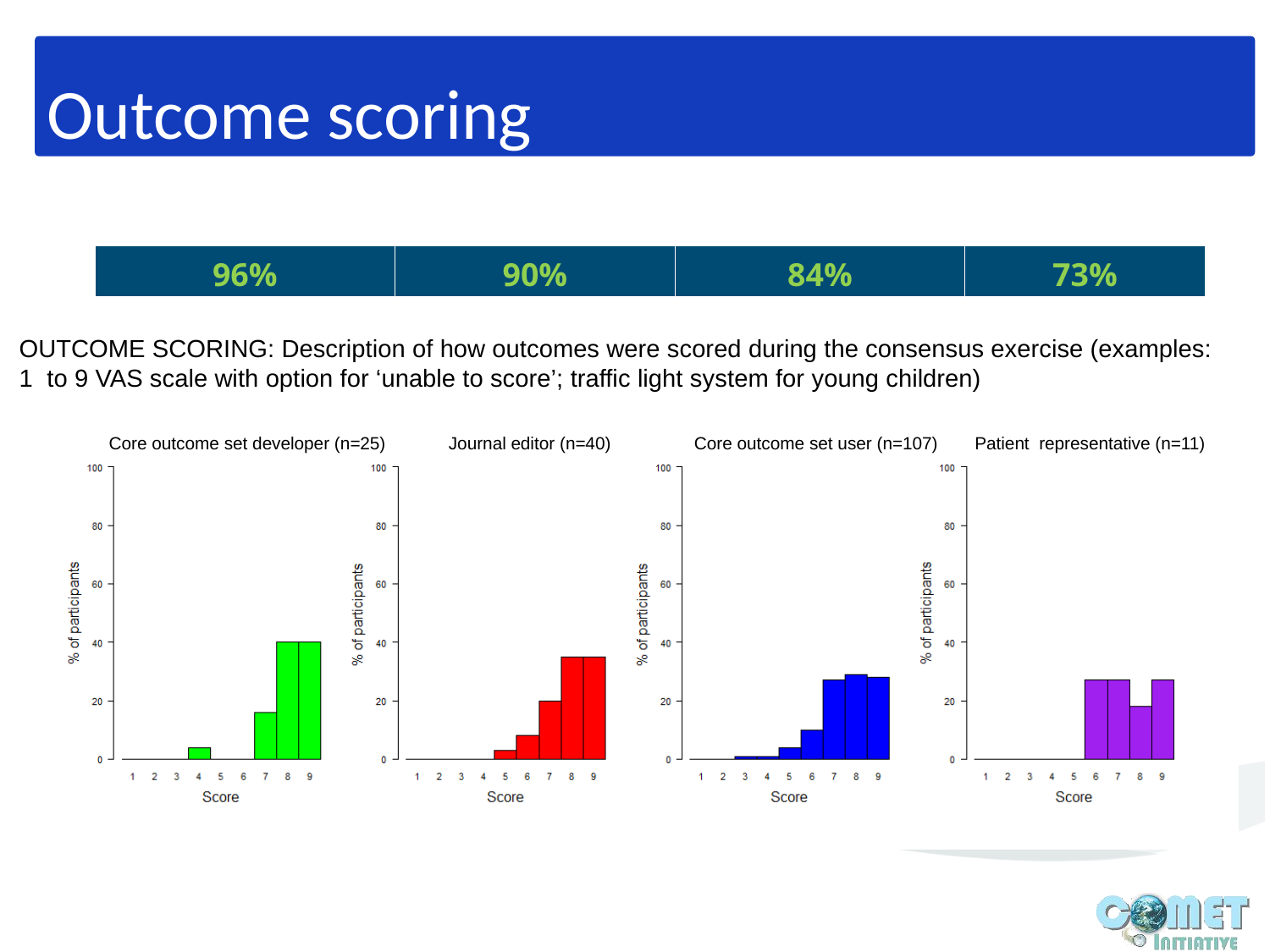

# Outcome scoring
| 96% | 90% | 84% | 73% |
| --- | --- | --- | --- |
OUTCOME SCORING: Description of how outcomes were scored during the consensus exercise (examples:
1 to 9 VAS scale with option for ‘unable to score’; traffic light system for young children)
Core outcome set developer (n=25)
Journal editor (n=40)
Core outcome set user (n=107)
Patient representative (n=11)

## Slide 33
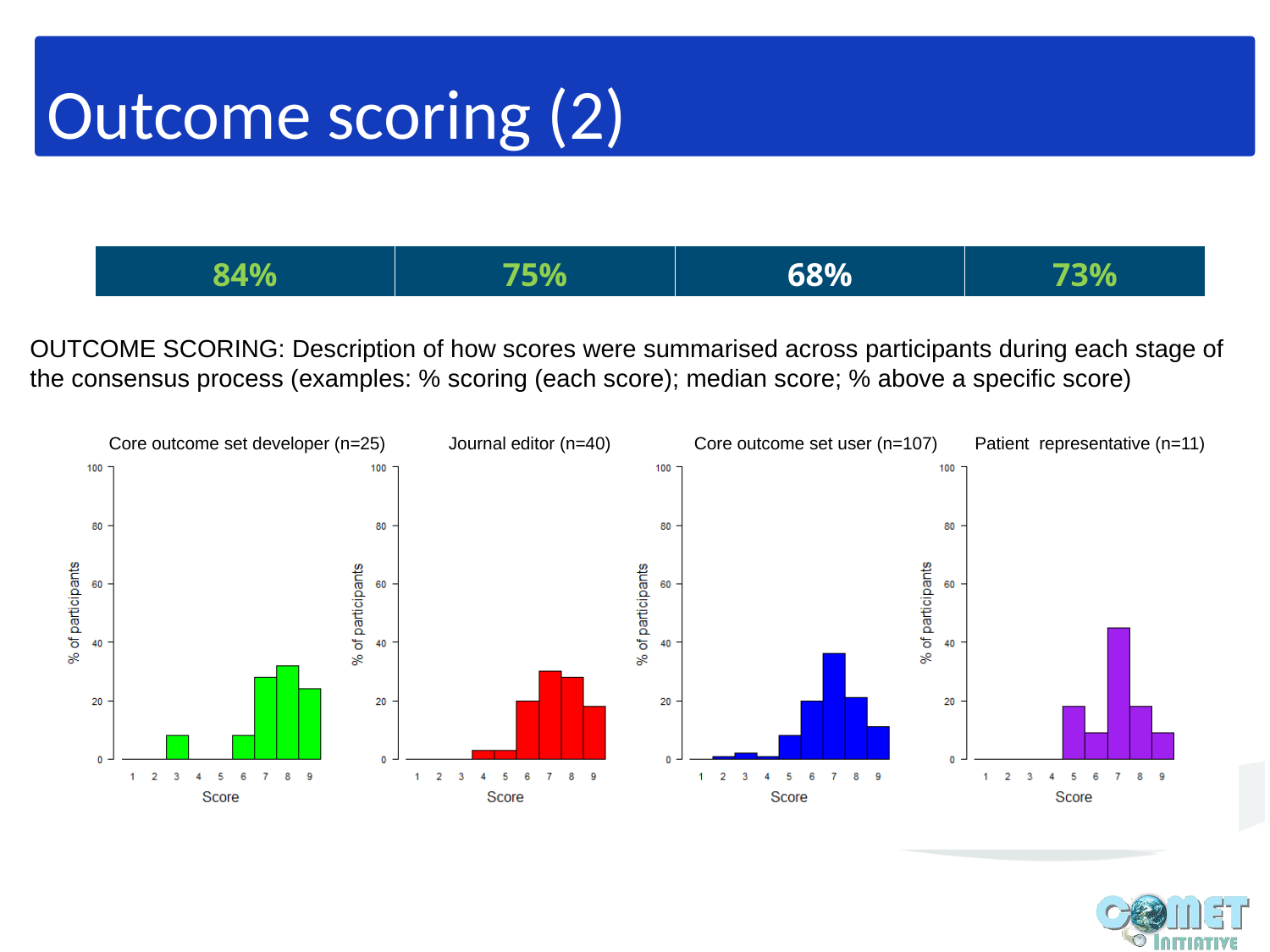

# Outcome scoring (2)
| 84% | 75% | 68% | 73% |
| --- | --- | --- | --- |
OUTCOME SCORING: Description of how scores were summarised across participants during each stage of
the consensus process (examples: % scoring (each score); median score; % above a specific score)
Core outcome set developer (n=25)
Journal editor (n=40)
Core outcome set user (n=107)
Patient representative (n=11)

## Slide 34
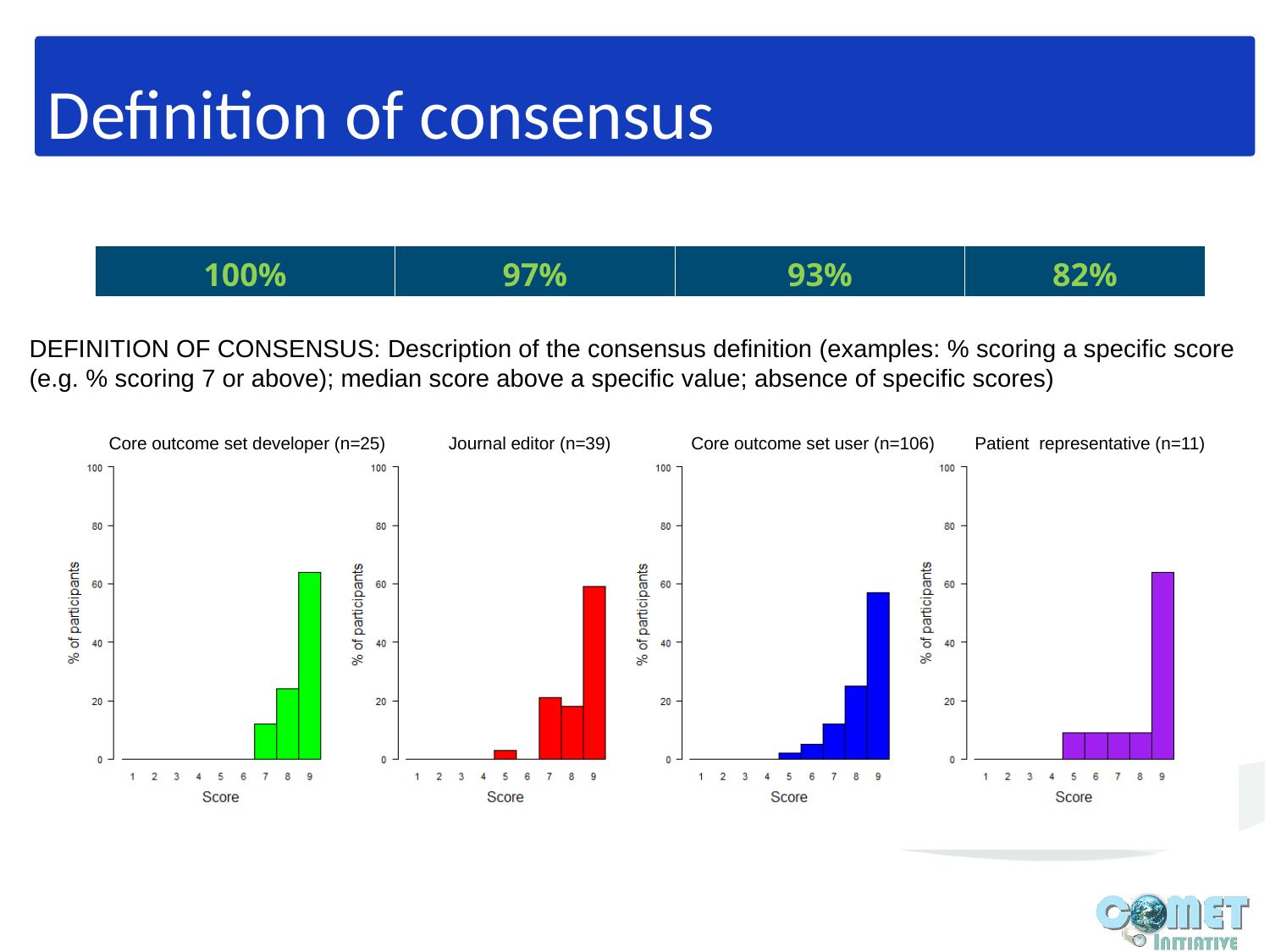

# Definition of consensus
| 100% | 97% | 93% | 82% |
| --- | --- | --- | --- |
DEFINITION OF CONSENSUS: Description of the consensus definition (examples: % scoring a specific score
(e.g. % scoring 7 or above); median score above a specific value; absence of specific scores)
Core outcome set developer (n=25)
Journal editor (n=39)
Core outcome set user (n=106)
Patient representative (n=11)

## Slide 35
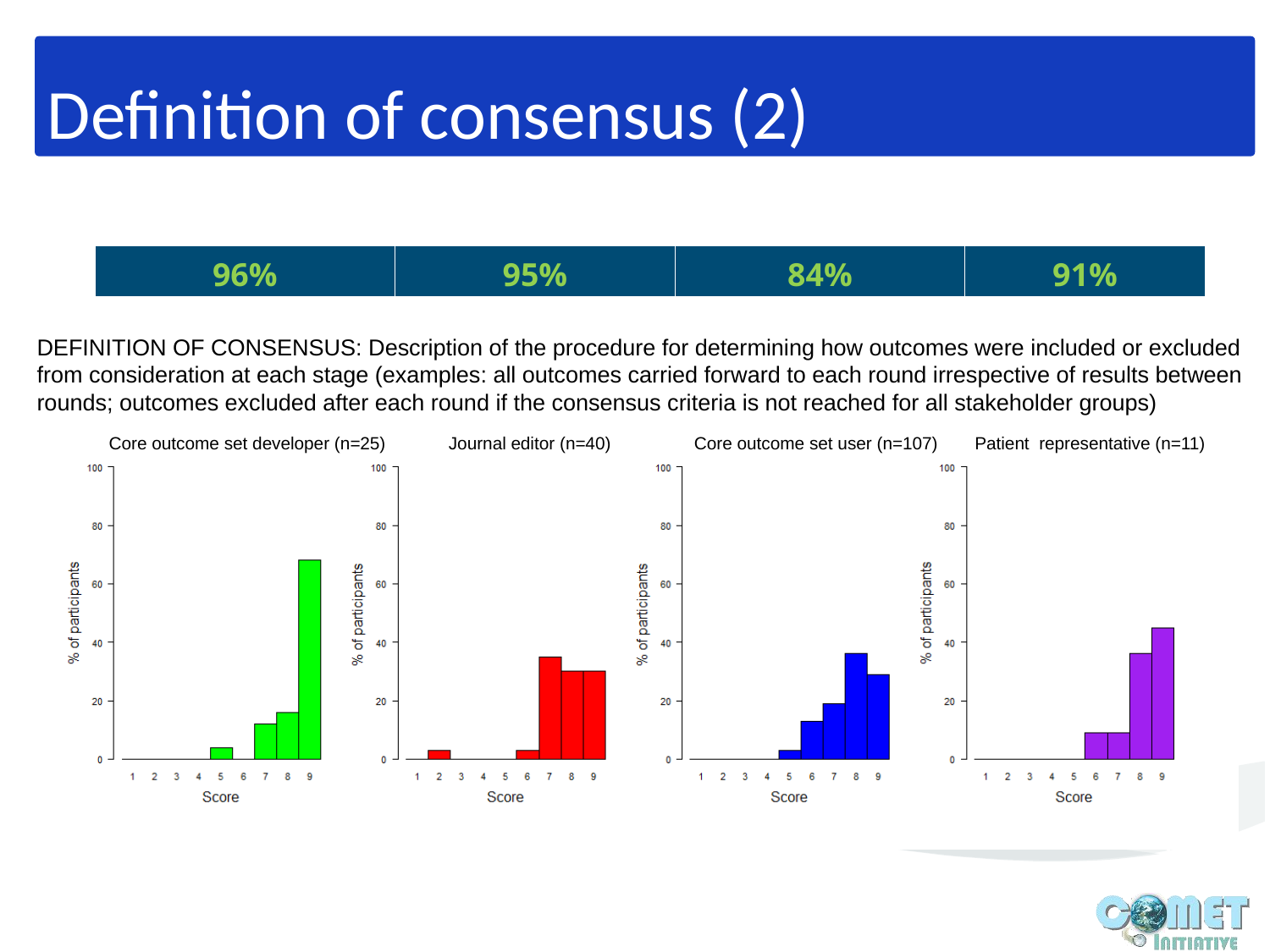

# Definition of consensus (2)
| 96% | 95% | 84% | 91% |
| --- | --- | --- | --- |
DEFINITION OF CONSENSUS: Description of the procedure for determining how outcomes were included or excluded
from consideration at each stage (examples: all outcomes carried forward to each round irrespective of results between
rounds; outcomes excluded after each round if the consensus criteria is not reached for all stakeholder groups)
Core outcome set developer (n=25)
Journal editor (n=40)
Core outcome set user (n=107)
Patient representative (n=11)

## Slide 36
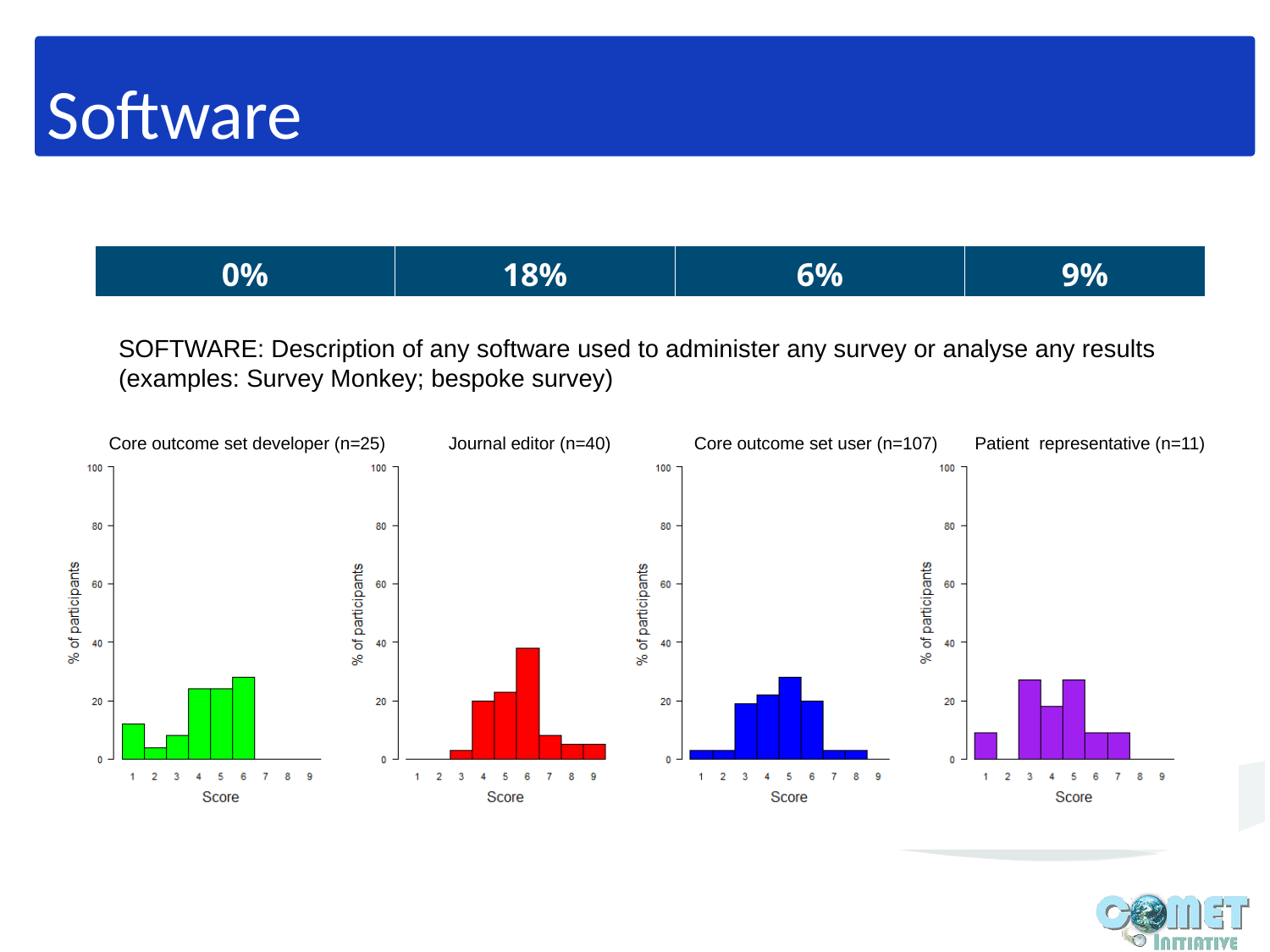

# Software
| 0% | 18% | 6% | 9% |
| --- | --- | --- | --- |
SOFTWARE: Description of any software used to administer any survey or analyse any results
(examples: Survey Monkey; bespoke survey)
Core outcome set developer (n=25)
Journal editor (n=40)
Core outcome set user (n=107)
Patient representative (n=11)

## Slide 37
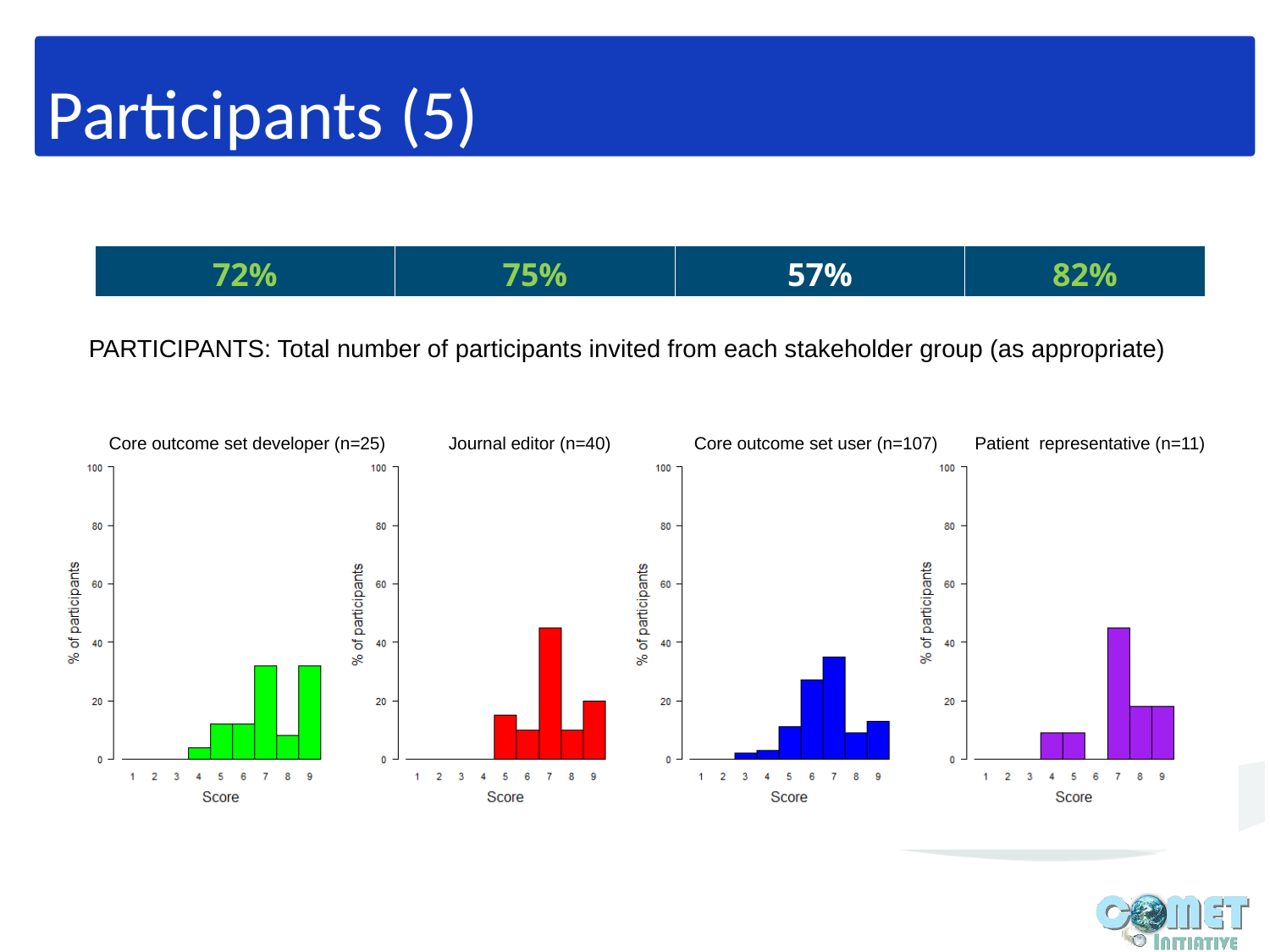

# Participants (5)
| 72% | 75% | 57% | 82% |
| --- | --- | --- | --- |
PARTICIPANTS: Total number of participants invited from each stakeholder group (as appropriate)
Core outcome set developer (n=25)
Journal editor (n=40)
Core outcome set user (n=107)
Patient representative (n=11)

## Slide 38
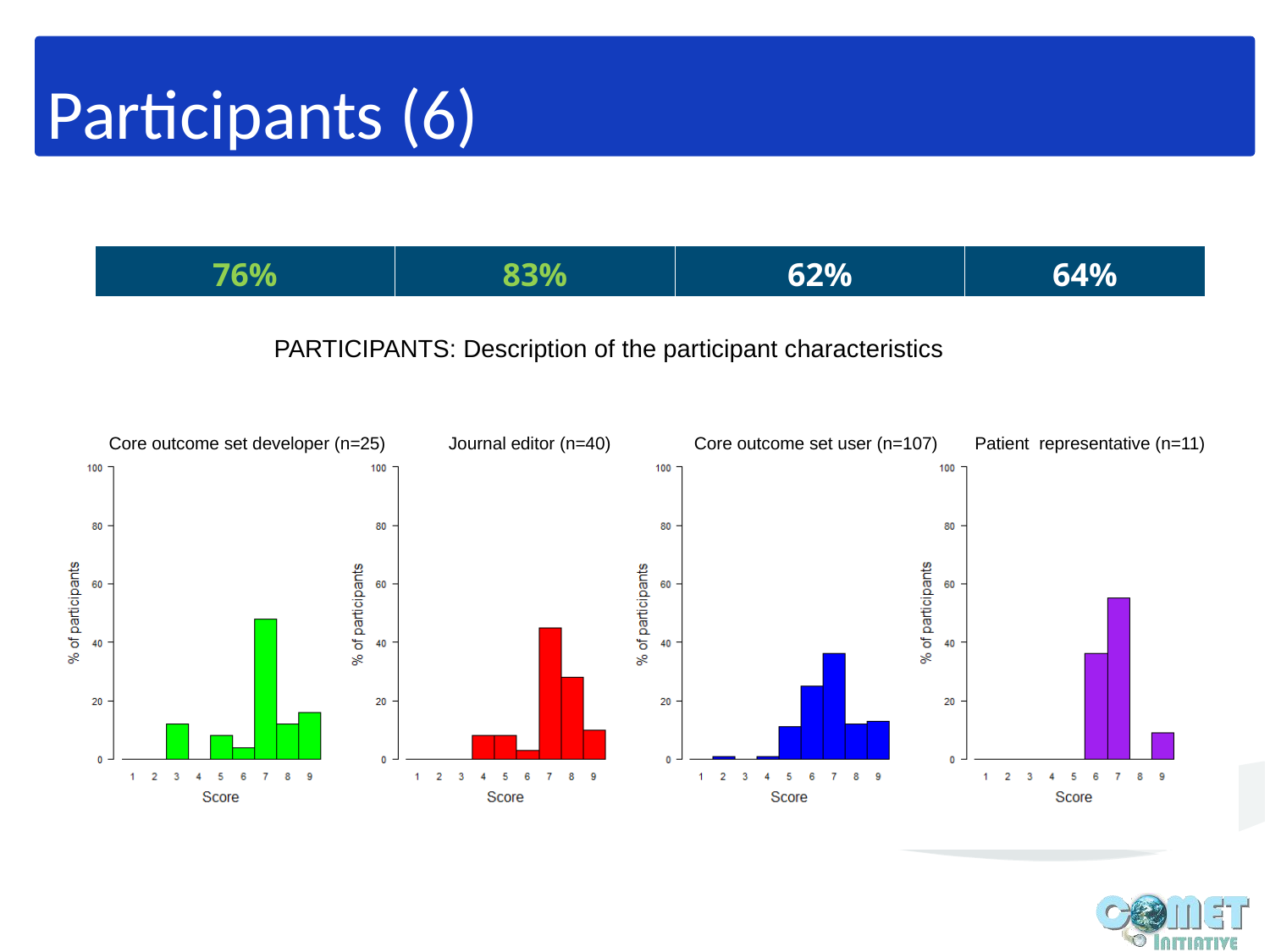

# Participants (6)
| 76% | 83% | 62% | 64% |
| --- | --- | --- | --- |
PARTICIPANTS: Description of the participant characteristics
Core outcome set developer (n=25)
Journal editor (n=40)
Core outcome set user (n=107)
Patient representative (n=11)

## Slide 39
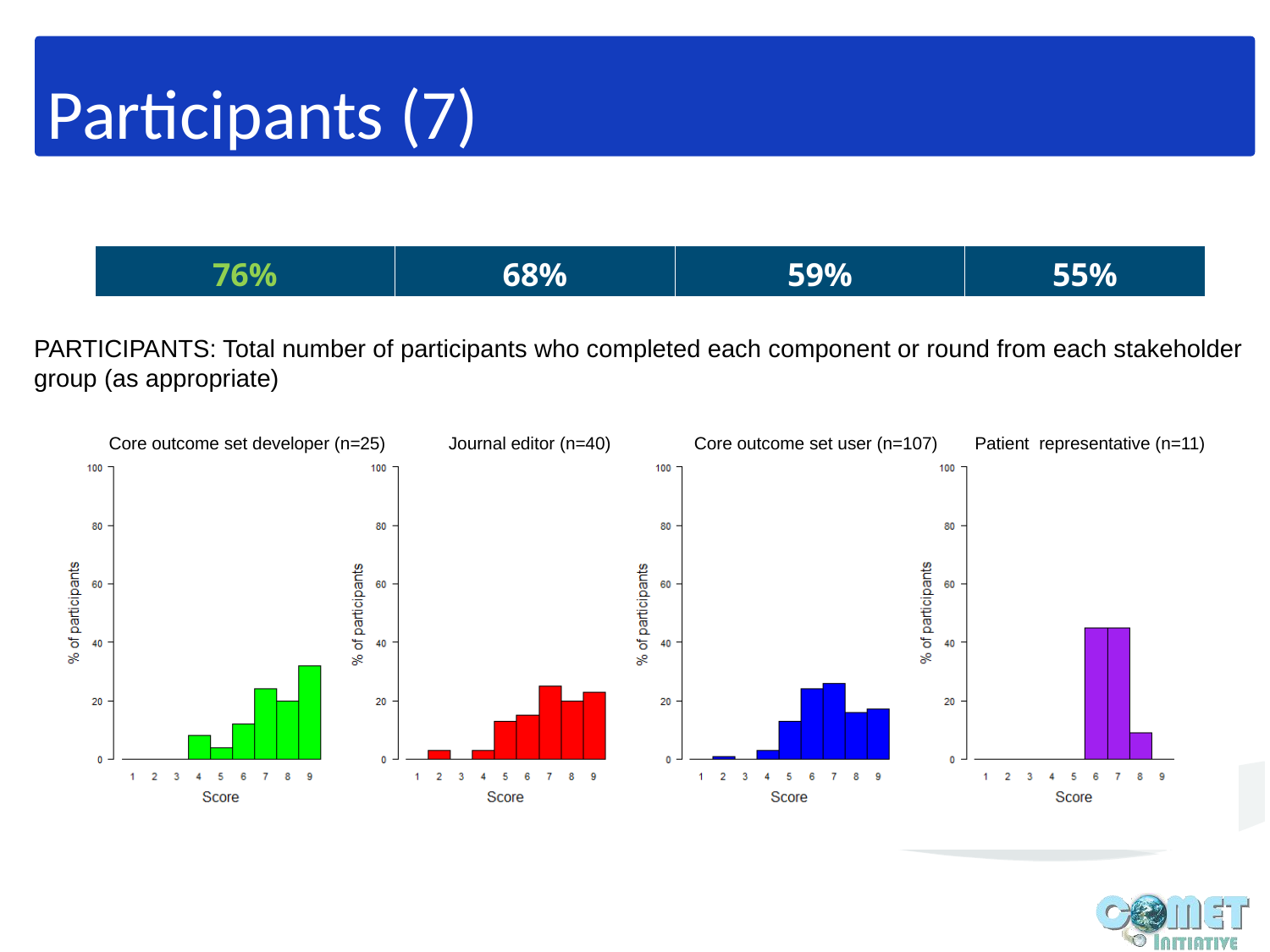

# Participants (7)
| 76% | 68% | 59% | 55% |
| --- | --- | --- | --- |
PARTICIPANTS: Total number of participants who completed each component or round from each stakeholder
group (as appropriate)
Core outcome set developer (n=25)
Journal editor (n=40)
Core outcome set user (n=107)
Patient representative (n=11)

## Slide 40
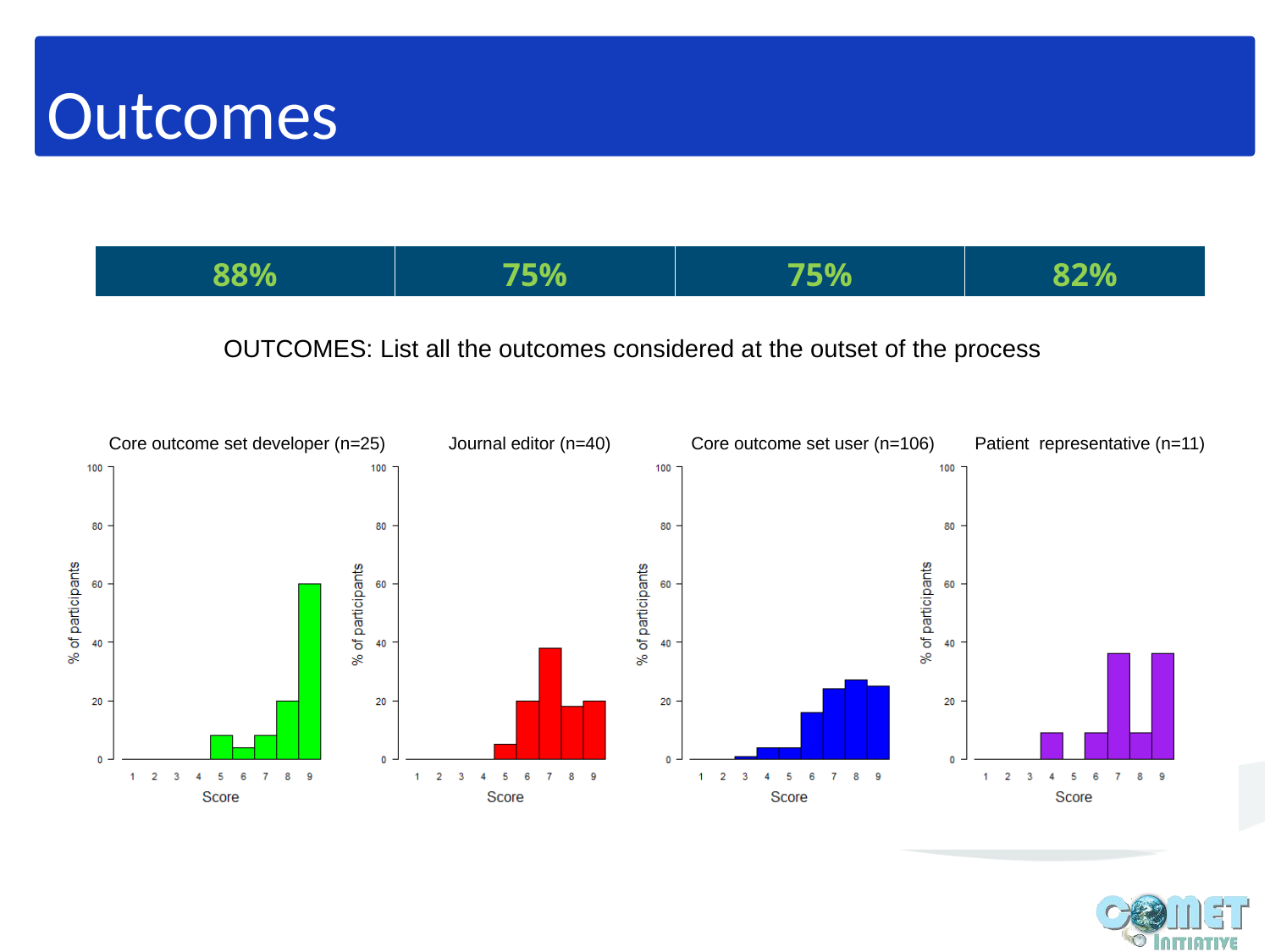

# Outcomes
| 88% | 75% | 75% | 82% |
| --- | --- | --- | --- |
OUTCOMES: List all the outcomes considered at the outset of the process
Core outcome set developer (n=25)
Journal editor (n=40)
Core outcome set user (n=106)
Patient representative (n=11)

## Slide 41
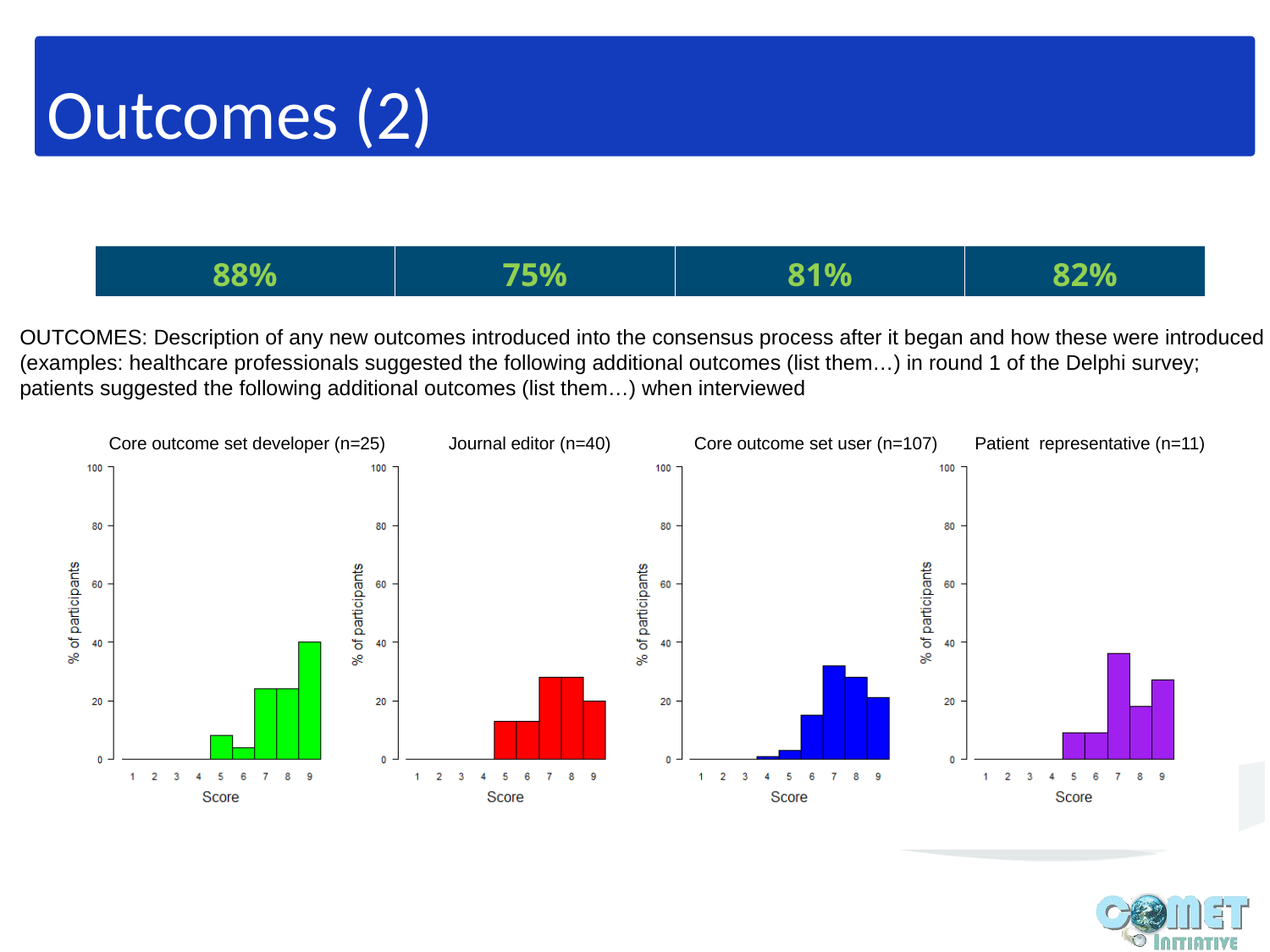

# Outcomes (2)
| 88% | 75% | 81% | 82% |
| --- | --- | --- | --- |
OUTCOMES: Description of any new outcomes introduced into the consensus process after it began and how these were introduced
(examples: healthcare professionals suggested the following additional outcomes (list them…) in round 1 of the Delphi survey;
patients suggested the following additional outcomes (list them…) when interviewed
Core outcome set developer (n=25)
Journal editor (n=40)
Core outcome set user (n=107)
Patient representative (n=11)

## Slide 42
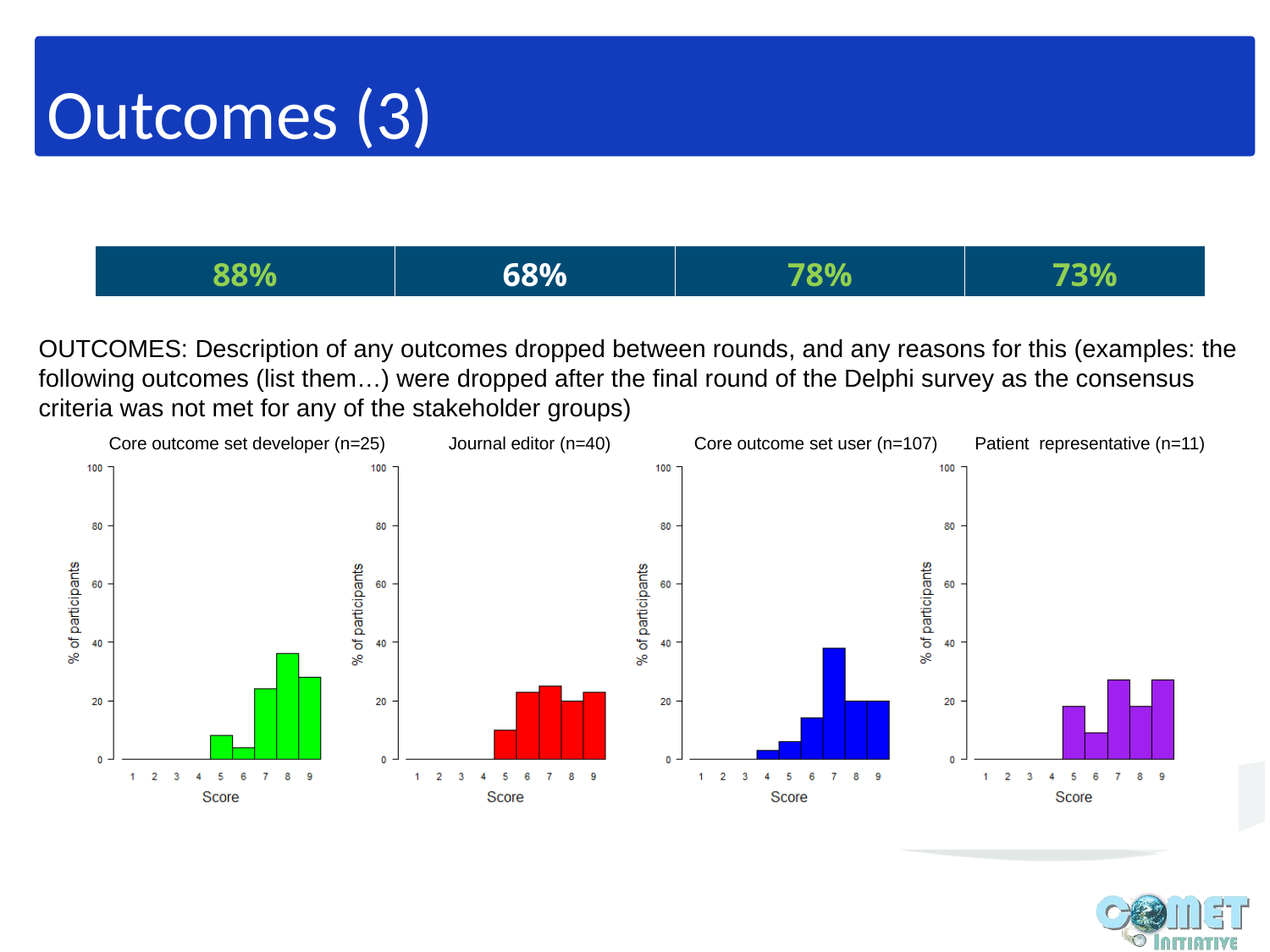

# Outcomes (3)
| 88% | 68% | 78% | 73% |
| --- | --- | --- | --- |
OUTCOMES: Description of any outcomes dropped between rounds, and any reasons for this (examples: the
following outcomes (list them…) were dropped after the final round of the Delphi survey as the consensus
criteria was not met for any of the stakeholder groups)
Core outcome set developer (n=25)
Journal editor (n=40)
Core outcome set user (n=107)
Patient representative (n=11)

## Slide 43
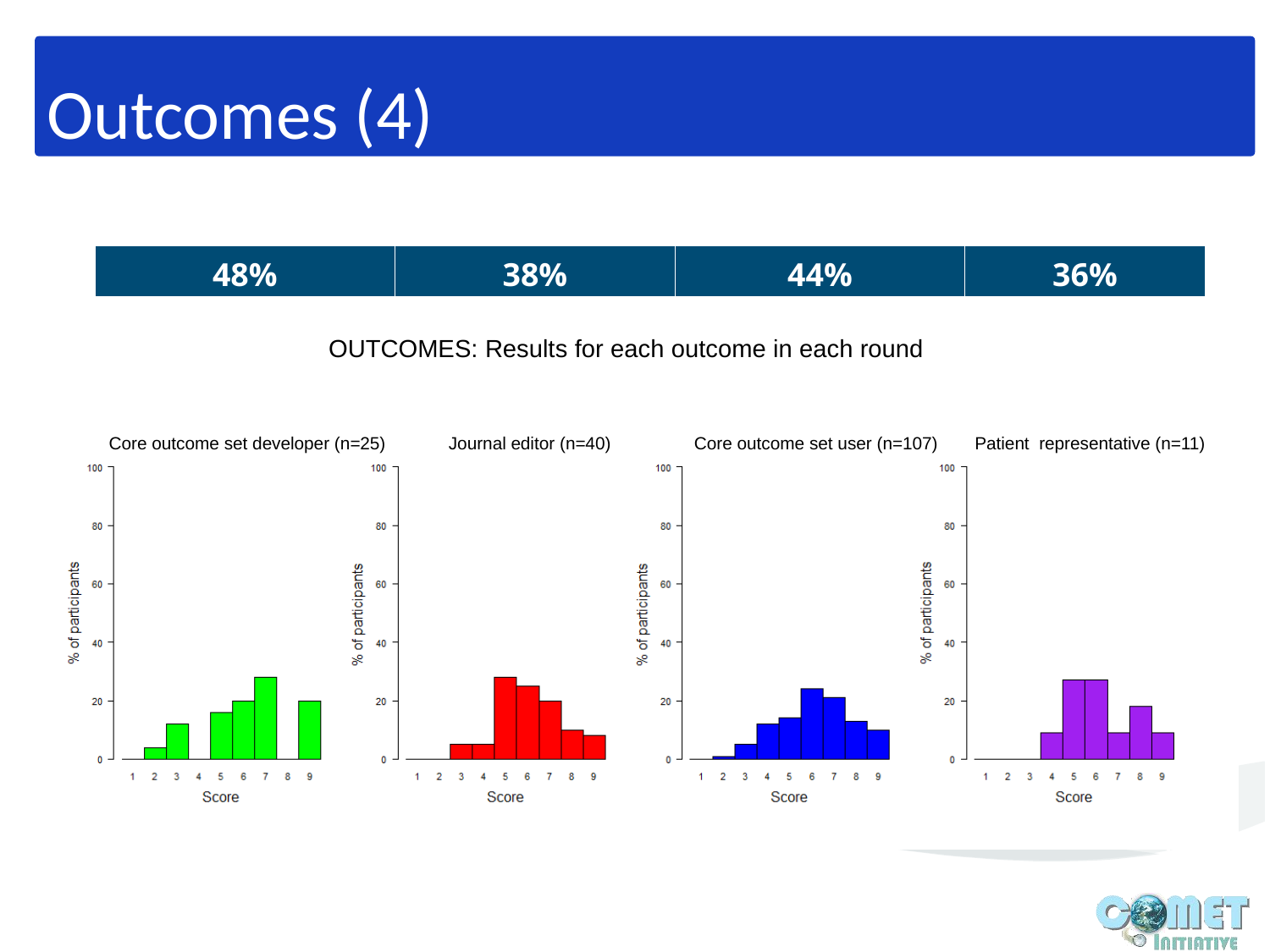

# Outcomes (4)
| 48% | 38% | 44% | 36% |
| --- | --- | --- | --- |
OUTCOMES: Results for each outcome in each round
Core outcome set developer (n=25)
Journal editor (n=40)
Core outcome set user (n=107)
Patient representative (n=11)

## Slide 44
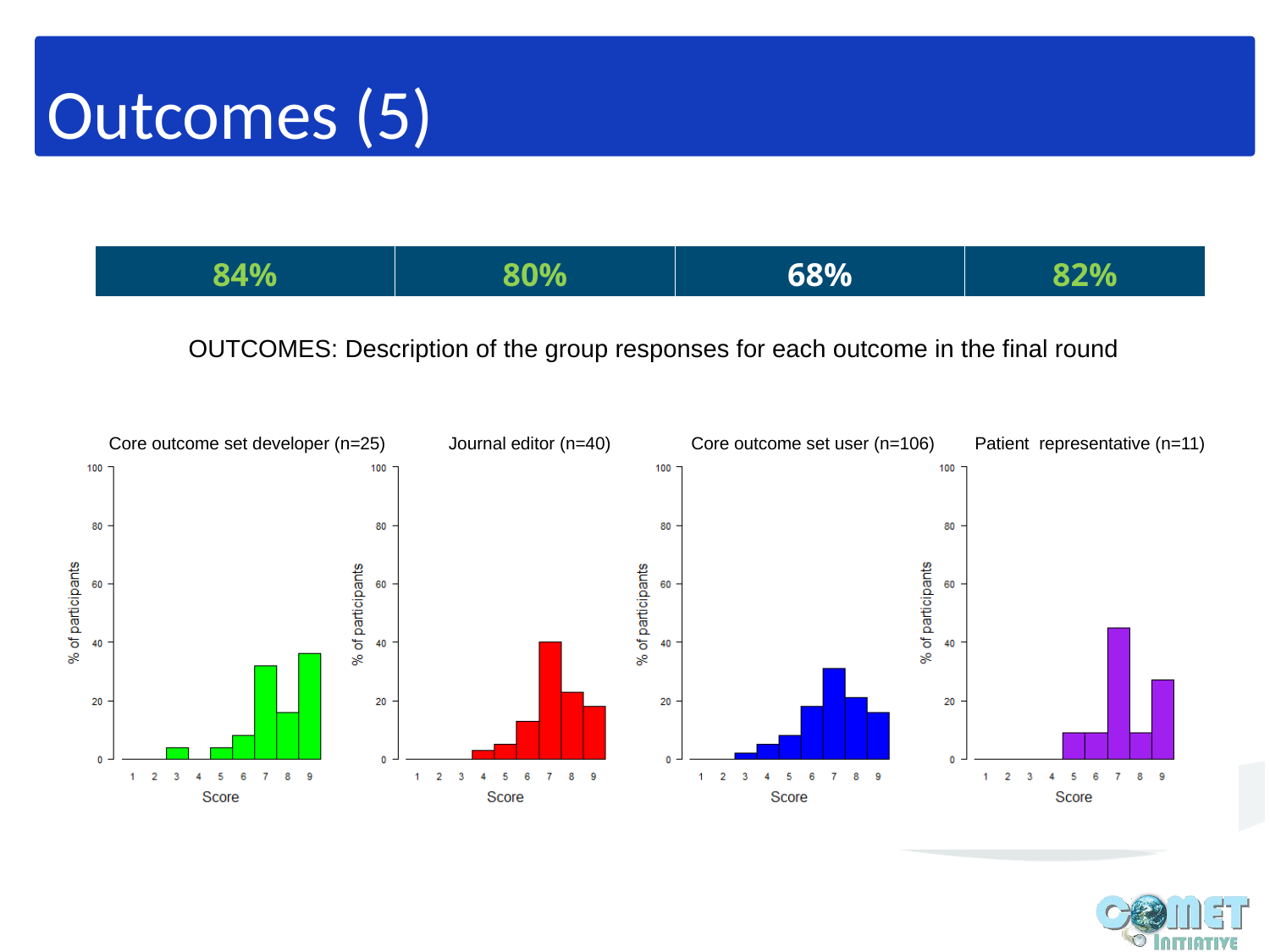

# Outcomes (5)
| 84% | 80% | 68% | 82% |
| --- | --- | --- | --- |
OUTCOMES: Description of the group responses for each outcome in the final round
Core outcome set developer (n=25)
Journal editor (n=40)
Core outcome set user (n=106)
Patient representative (n=11)

## Slide 45
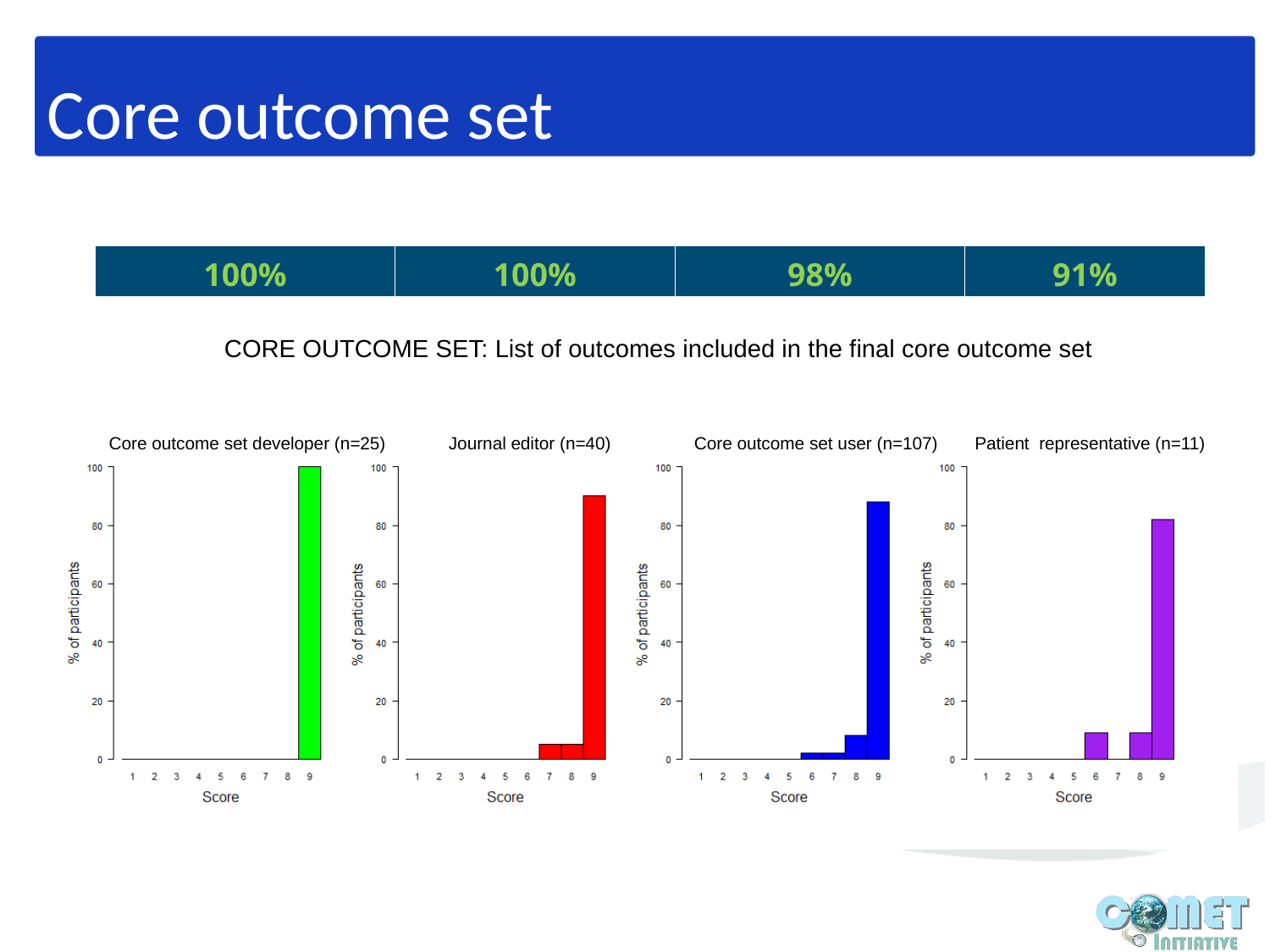

# Core outcome set
| 100% | 100% | 98% | 91% |
| --- | --- | --- | --- |
CORE OUTCOME SET: List of outcomes included in the final core outcome set
Core outcome set developer (n=25)
Journal editor (n=40)
Core outcome set user (n=107)
Patient representative (n=11)

## Slide 46
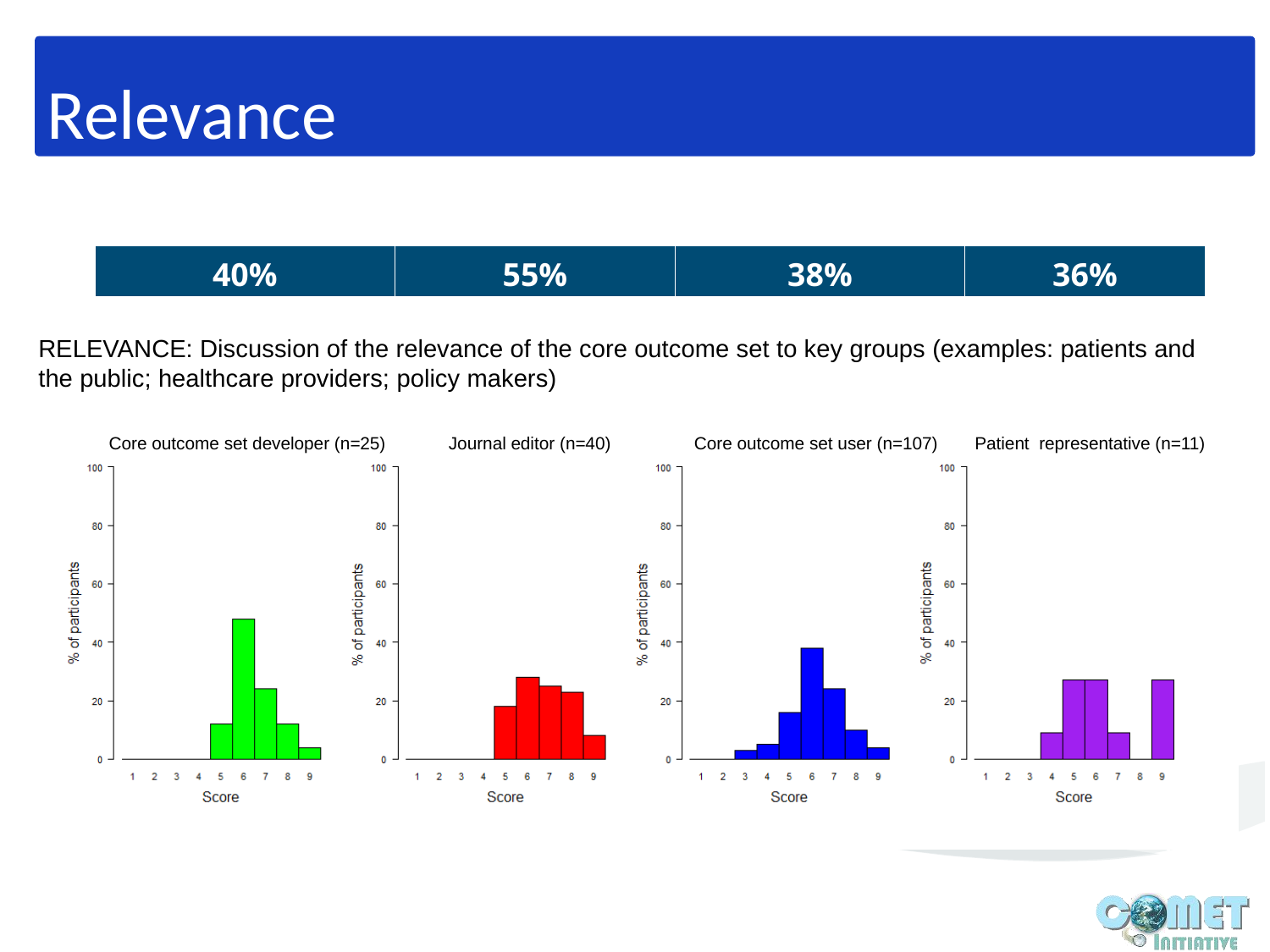

# Relevance
| 40% | 55% | 38% | 36% |
| --- | --- | --- | --- |
RELEVANCE: Discussion of the relevance of the core outcome set to key groups (examples: patients and
the public; healthcare providers; policy makers)
Core outcome set developer (n=25)
Journal editor (n=40)
Core outcome set user (n=107)
Patient representative (n=11)

## Slide 47
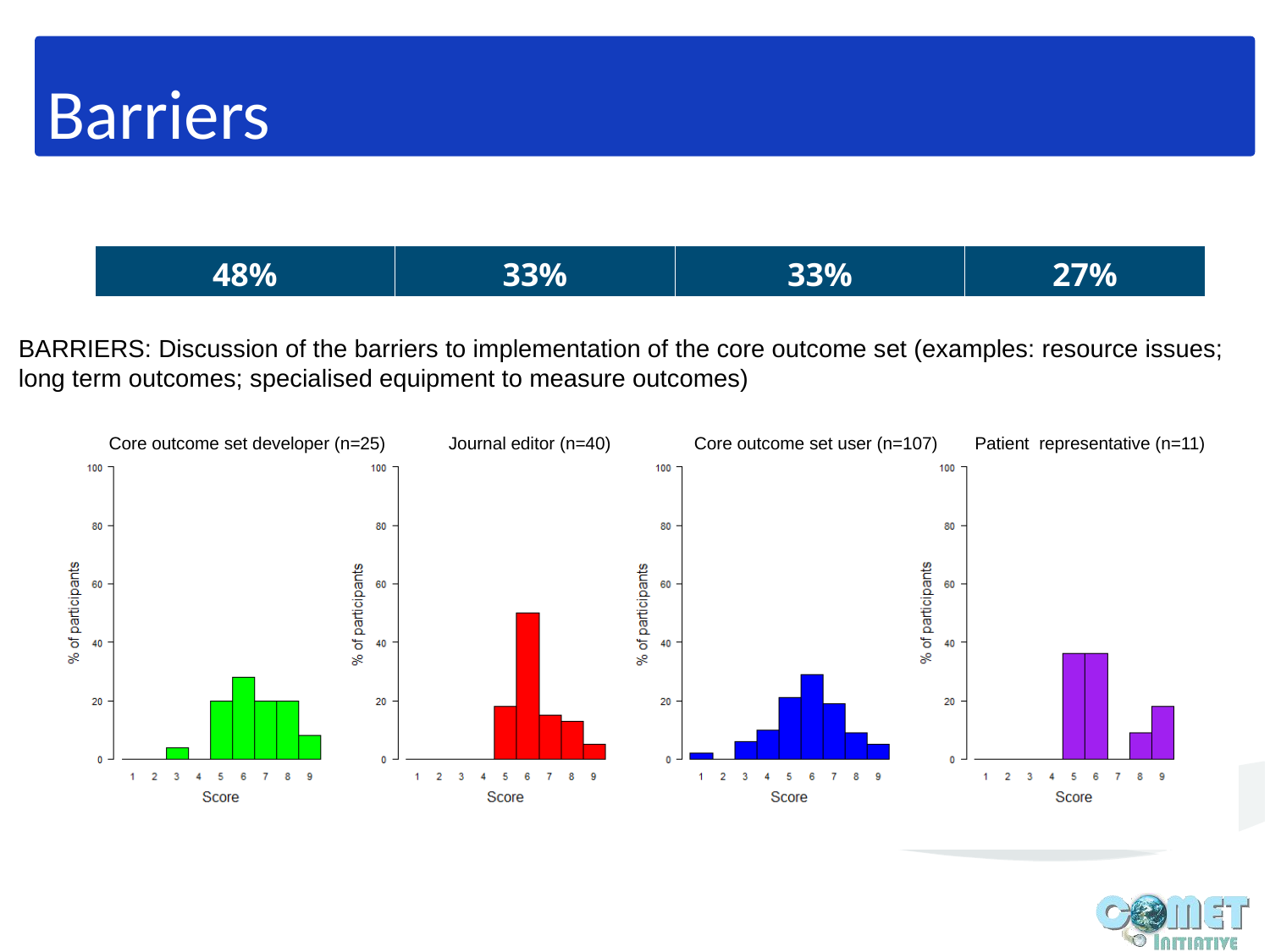

# Barriers
| 48% | 33% | 33% | 27% |
| --- | --- | --- | --- |
BARRIERS: Discussion of the barriers to implementation of the core outcome set (examples: resource issues;
long term outcomes; specialised equipment to measure outcomes)
Core outcome set developer (n=25)
Journal editor (n=40)
Core outcome set user (n=107)
Patient representative (n=11)

## Slide 48
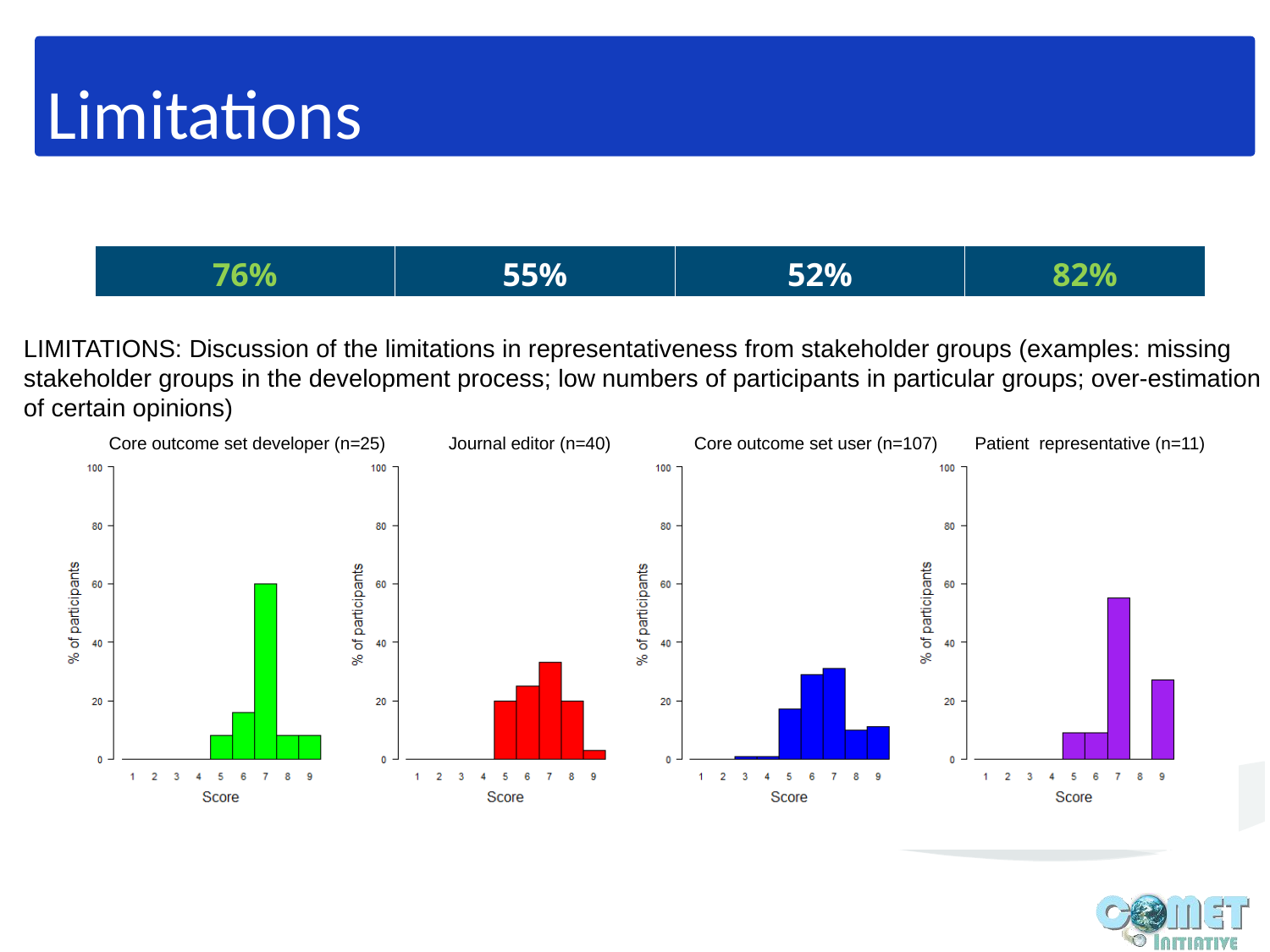

# Limitations
| 76% | 55% | 52% | 82% |
| --- | --- | --- | --- |
LIMITATIONS: Discussion of the limitations in representativeness from stakeholder groups (examples: missing
stakeholder groups in the development process; low numbers of participants in particular groups; over-estimation
of certain opinions)
Core outcome set developer (n=25)
Journal editor (n=40)
Core outcome set user (n=107)
Patient representative (n=11)

## Slide 49
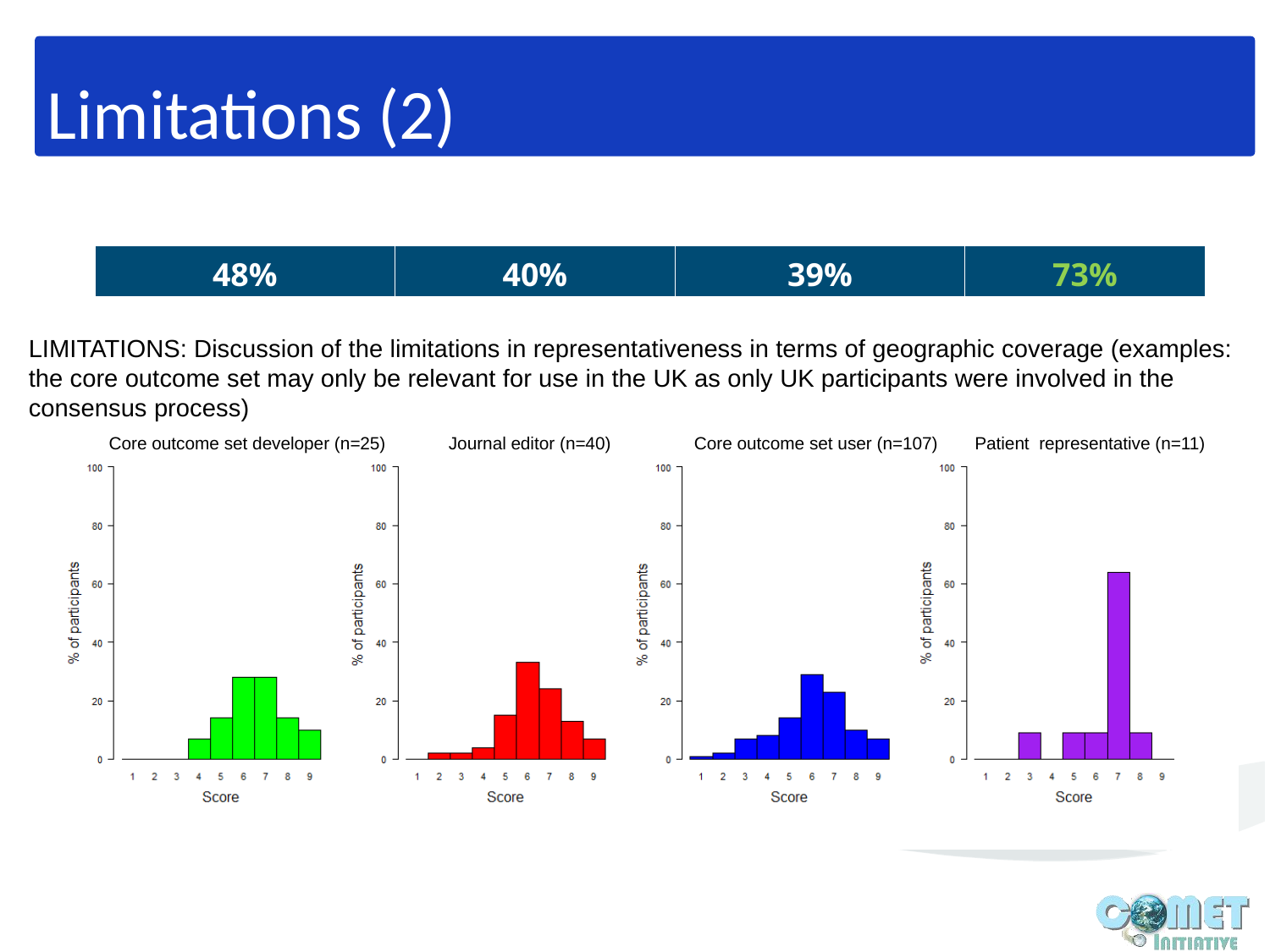

# Limitations (2)
| 48% | 40% | 39% | 73% |
| --- | --- | --- | --- |
LIMITATIONS: Discussion of the limitations in representativeness in terms of geographic coverage (examples:
the core outcome set may only be relevant for use in the UK as only UK participants were involved in the
consensus process)
Core outcome set developer (n=25)
Journal editor (n=40)
Core outcome set user (n=107)
Patient representative (n=11)

## Slide 50
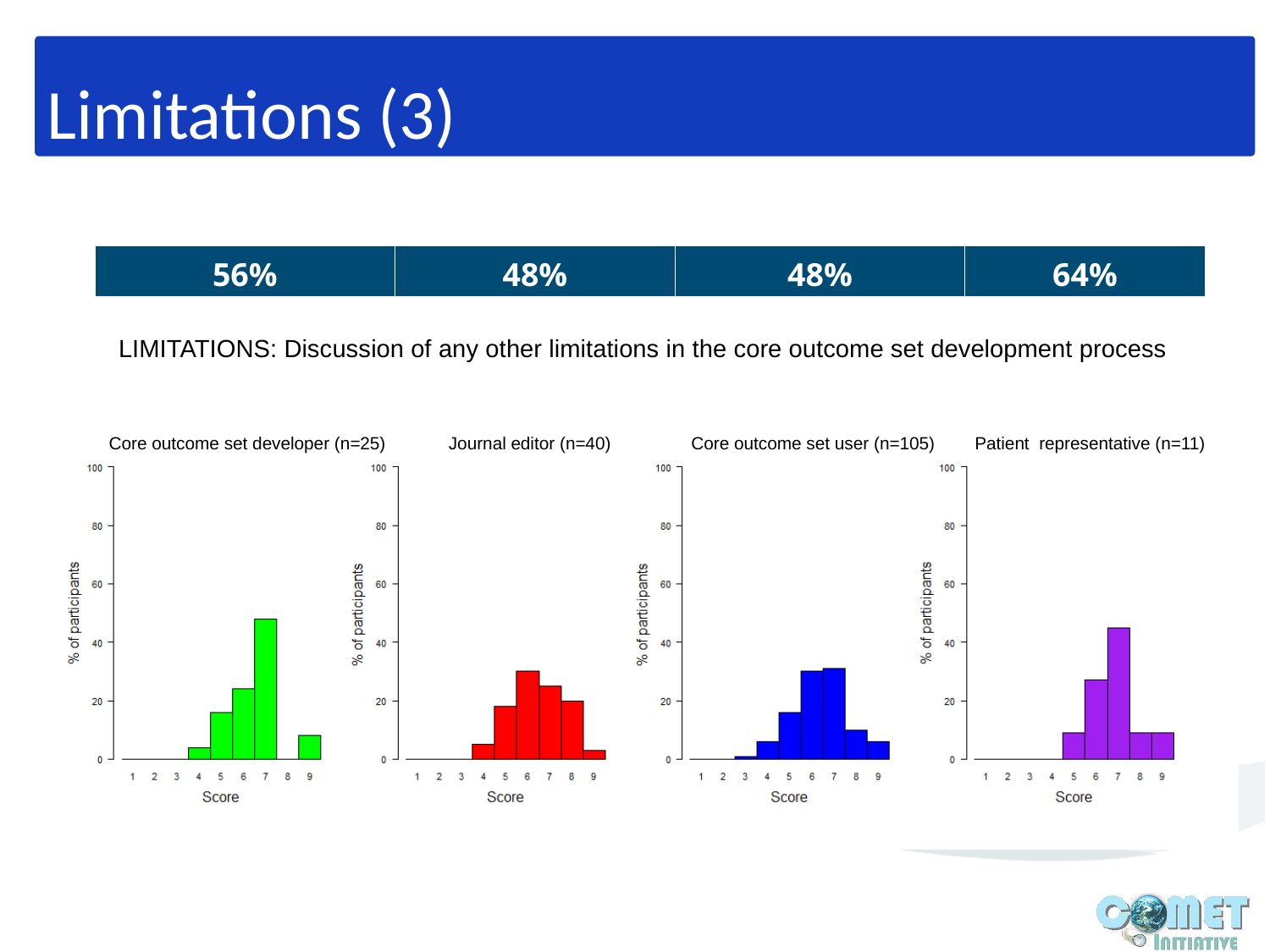

# Limitations (3)
| 56% | 48% | 48% | 64% |
| --- | --- | --- | --- |
LIMITATIONS: Discussion of any other limitations in the core outcome set development process
Core outcome set developer (n=25)
Journal editor (n=40)
Core outcome set user (n=105)
Patient representative (n=11)

## Slide 51
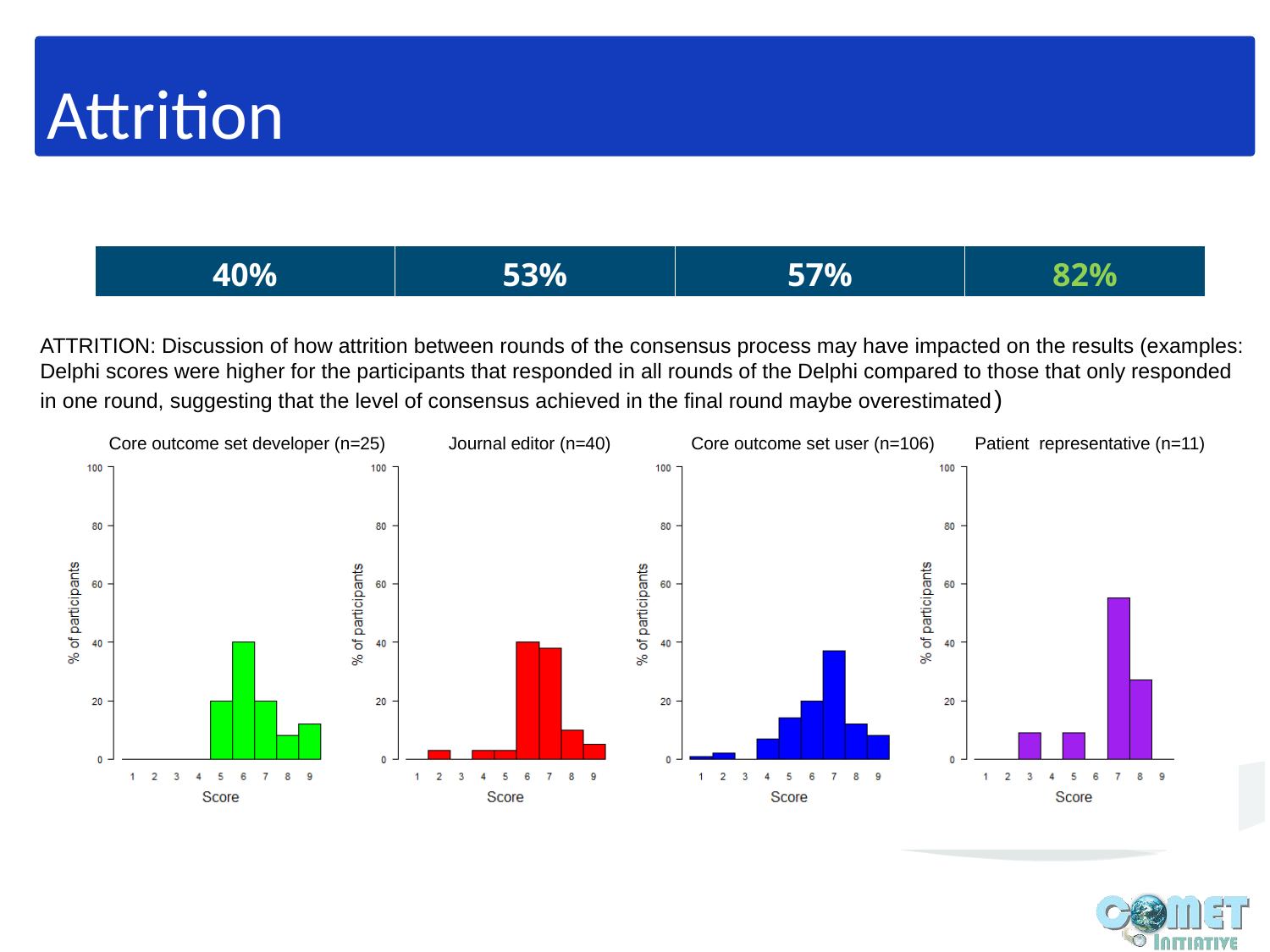

# Attrition
| 40% | 53% | 57% | 82% |
| --- | --- | --- | --- |
ATTRITION: Discussion of how attrition between rounds of the consensus process may have impacted on the results (examples:
Delphi scores were higher for the participants that responded in all rounds of the Delphi compared to those that only responded
in one round, suggesting that the level of consensus achieved in the final round maybe overestimated)
Core outcome set developer (n=25)
Journal editor (n=40)
Core outcome set user (n=106)
Patient representative (n=11)

## Slide 52
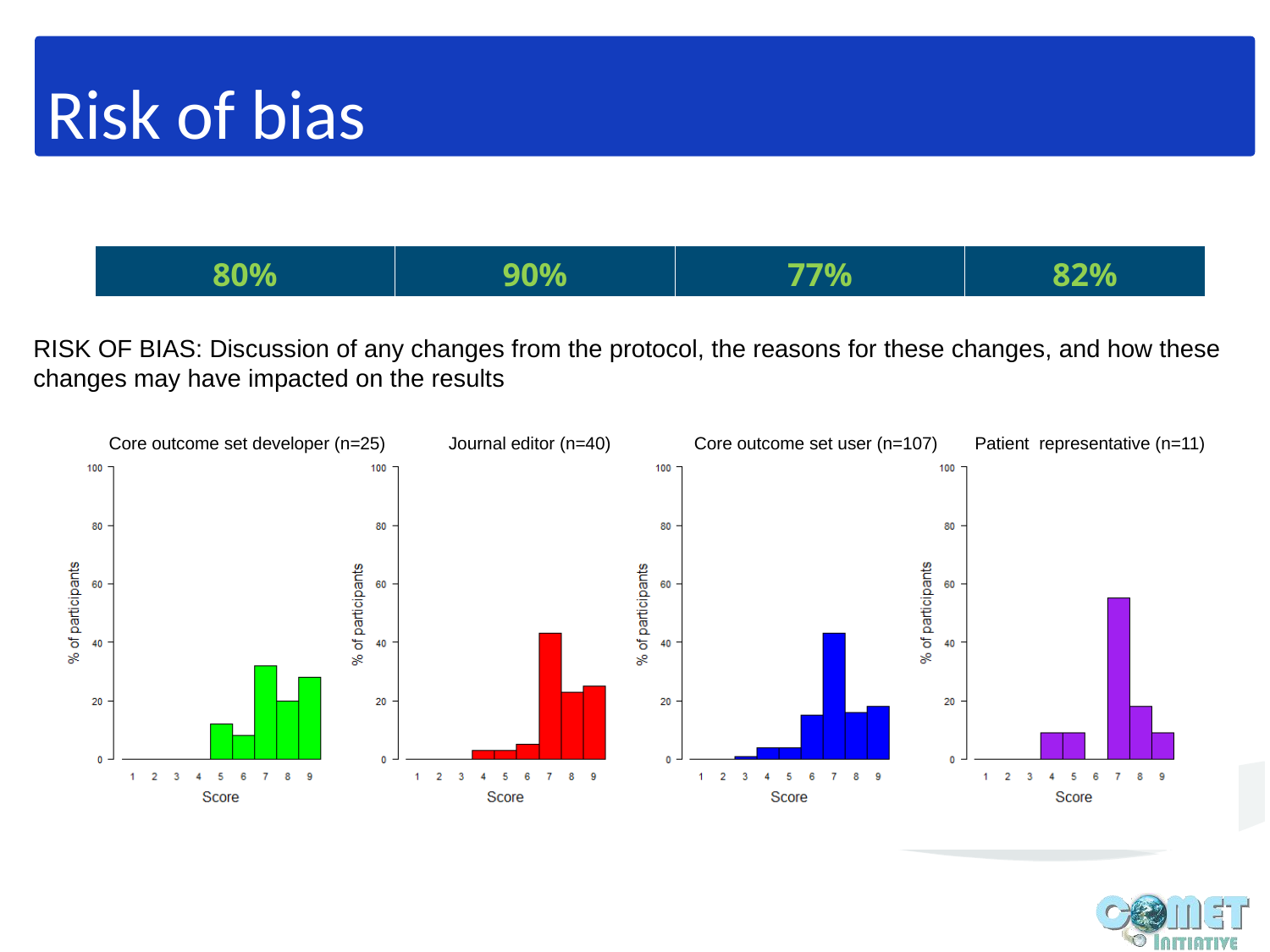

# Risk of bias
| 80% | 90% | 77% | 82% |
| --- | --- | --- | --- |
RISK OF BIAS: Discussion of any changes from the protocol, the reasons for these changes, and how these
changes may have impacted on the results
Core outcome set developer (n=25)
Journal editor (n=40)
Core outcome set user (n=107)
Patient representative (n=11)

## Slide 53
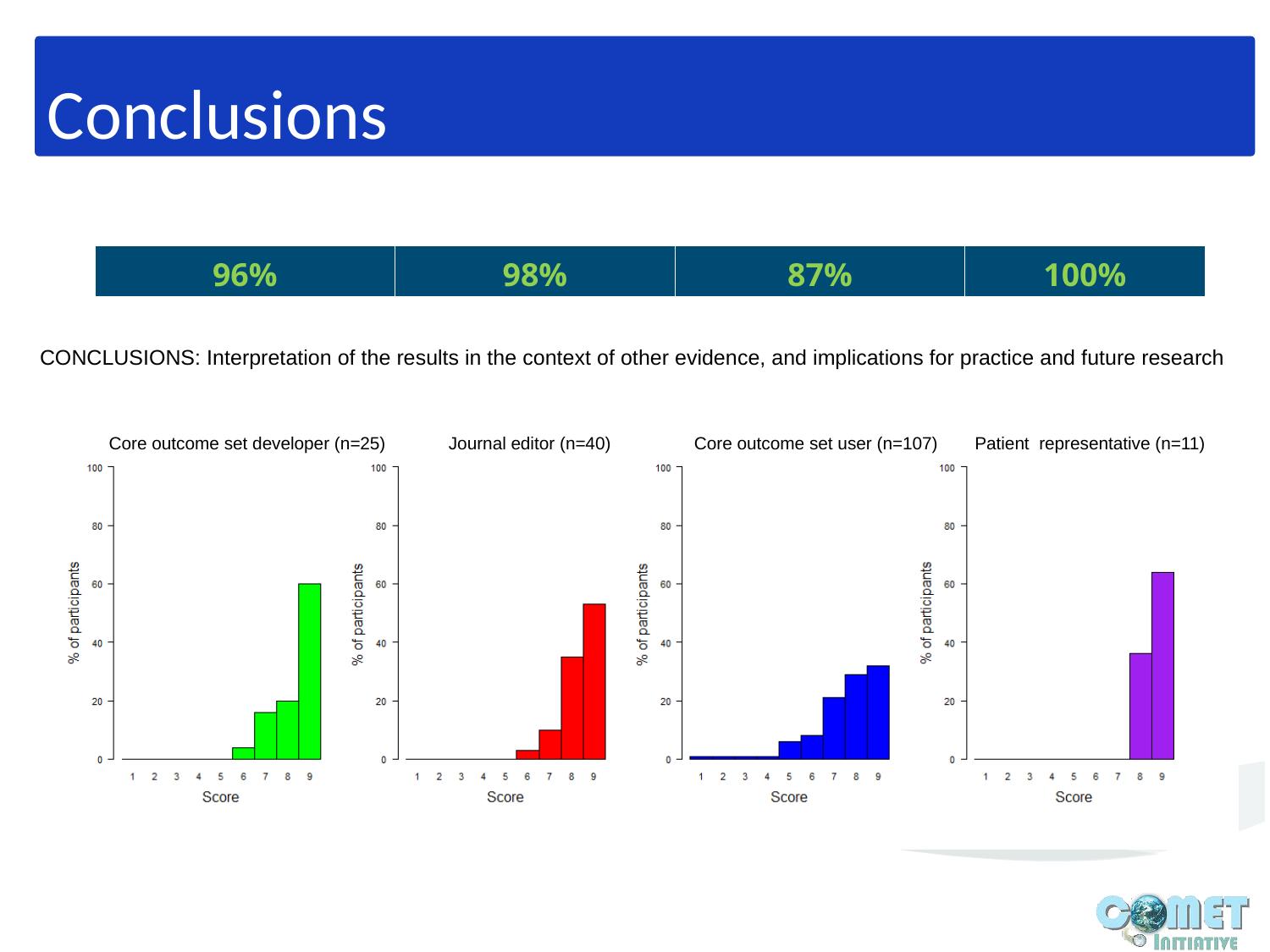

# Conclusions
| 96% | 98% | 87% | 100% |
| --- | --- | --- | --- |
CONCLUSIONS: Interpretation of the results in the context of other evidence, and implications for practice and future research
Core outcome set developer (n=25)
Journal editor (n=40)
Core outcome set user (n=107)
Patient representative (n=11)

## Slide 54
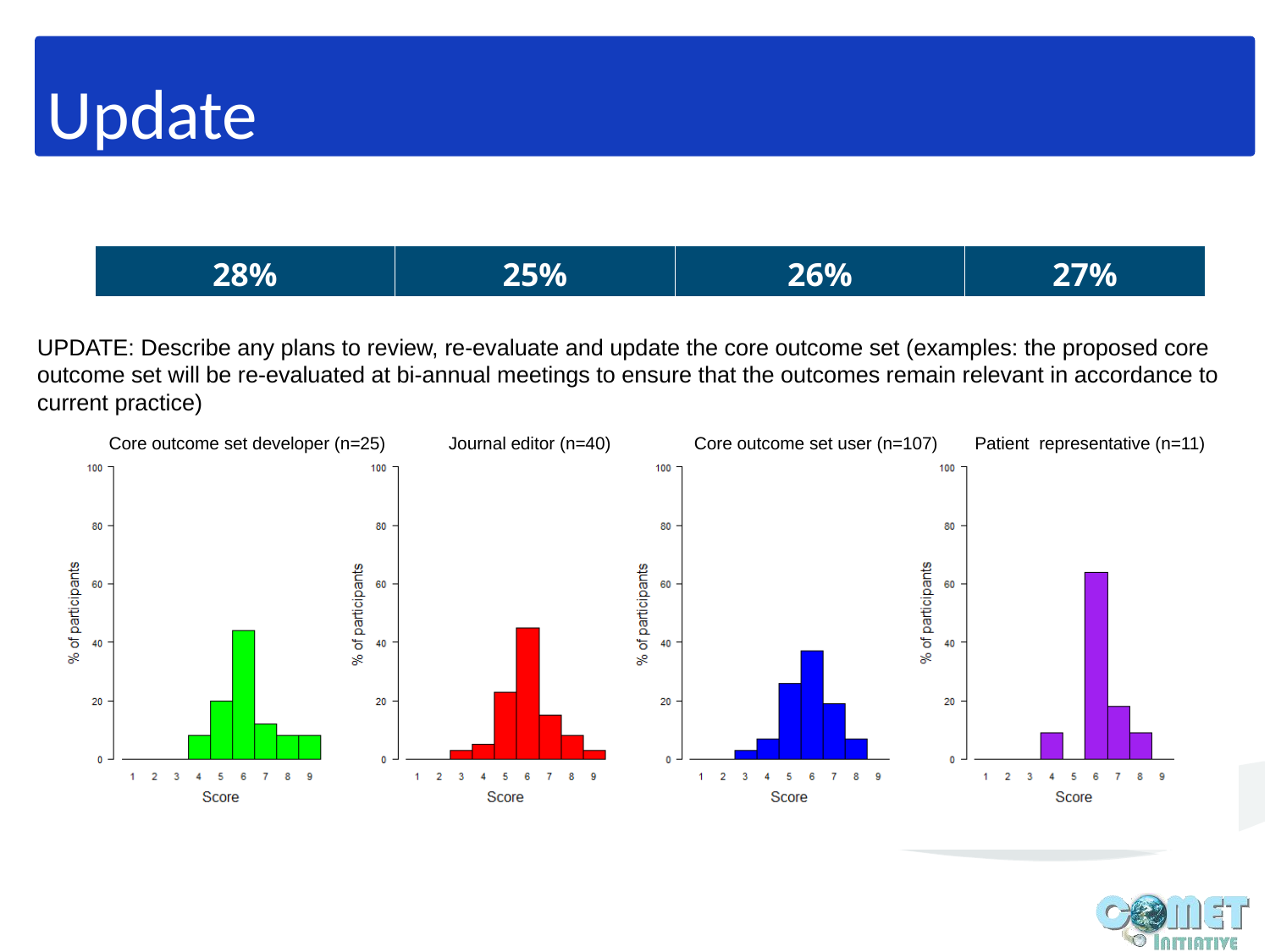

# Update
| 28% | 25% | 26% | 27% |
| --- | --- | --- | --- |
UPDATE: Describe any plans to review, re-evaluate and update the core outcome set (examples: the proposed core
outcome set will be re-evaluated at bi-annual meetings to ensure that the outcomes remain relevant in accordance to
current practice)
Core outcome set developer (n=25)
Journal editor (n=40)
Core outcome set user (n=107)
Patient representative (n=11)

## Slide 55
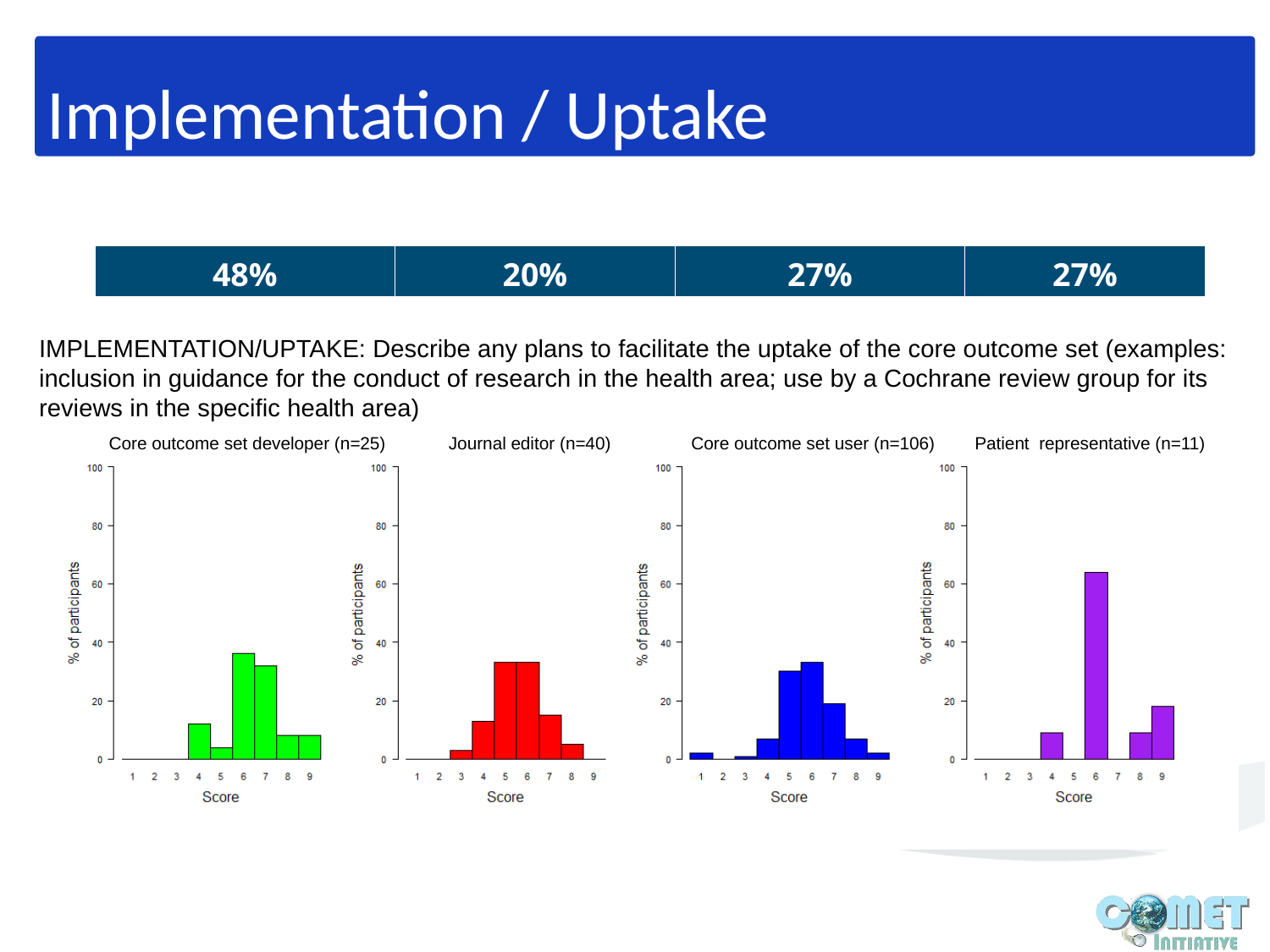

# Implementation / Uptake
| 48% | 20% | 27% | 27% |
| --- | --- | --- | --- |
IMPLEMENTATION/UPTAKE: Describe any plans to facilitate the uptake of the core outcome set (examples:
inclusion in guidance for the conduct of research in the health area; use by a Cochrane review group for its
reviews in the specific health area)
Core outcome set developer (n=25)
Journal editor (n=40)
Core outcome set user (n=106)
Patient representative (n=11)

## Slide 56
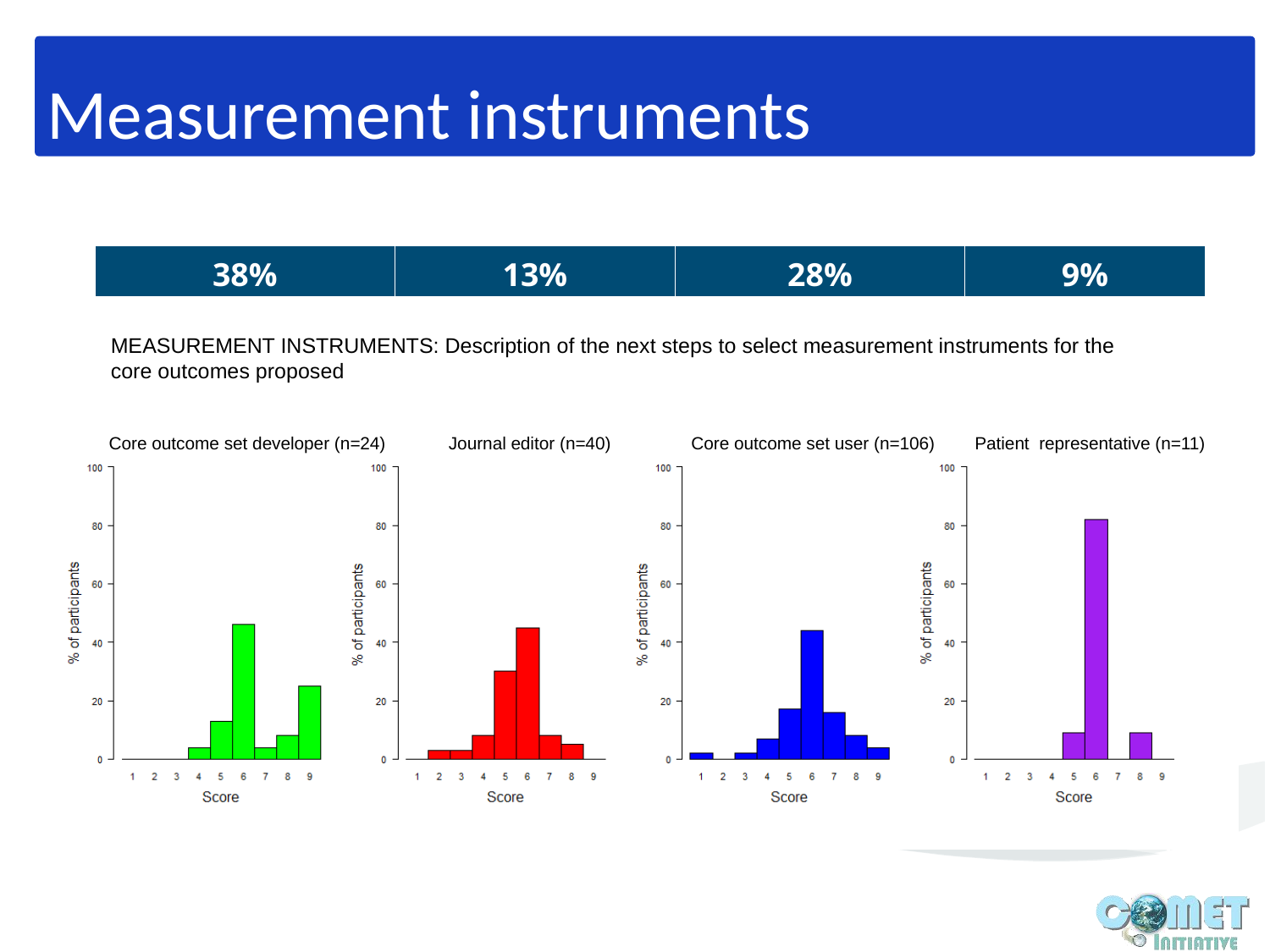

# Measurement instruments
| 38% | 13% | 28% | 9% |
| --- | --- | --- | --- |
MEASUREMENT INSTRUMENTS: Description of the next steps to select measurement instruments for the
core outcomes proposed
Core outcome set developer (n=24)
Journal editor (n=40)
Core outcome set user (n=106)
Patient representative (n=11)

## Slide 57
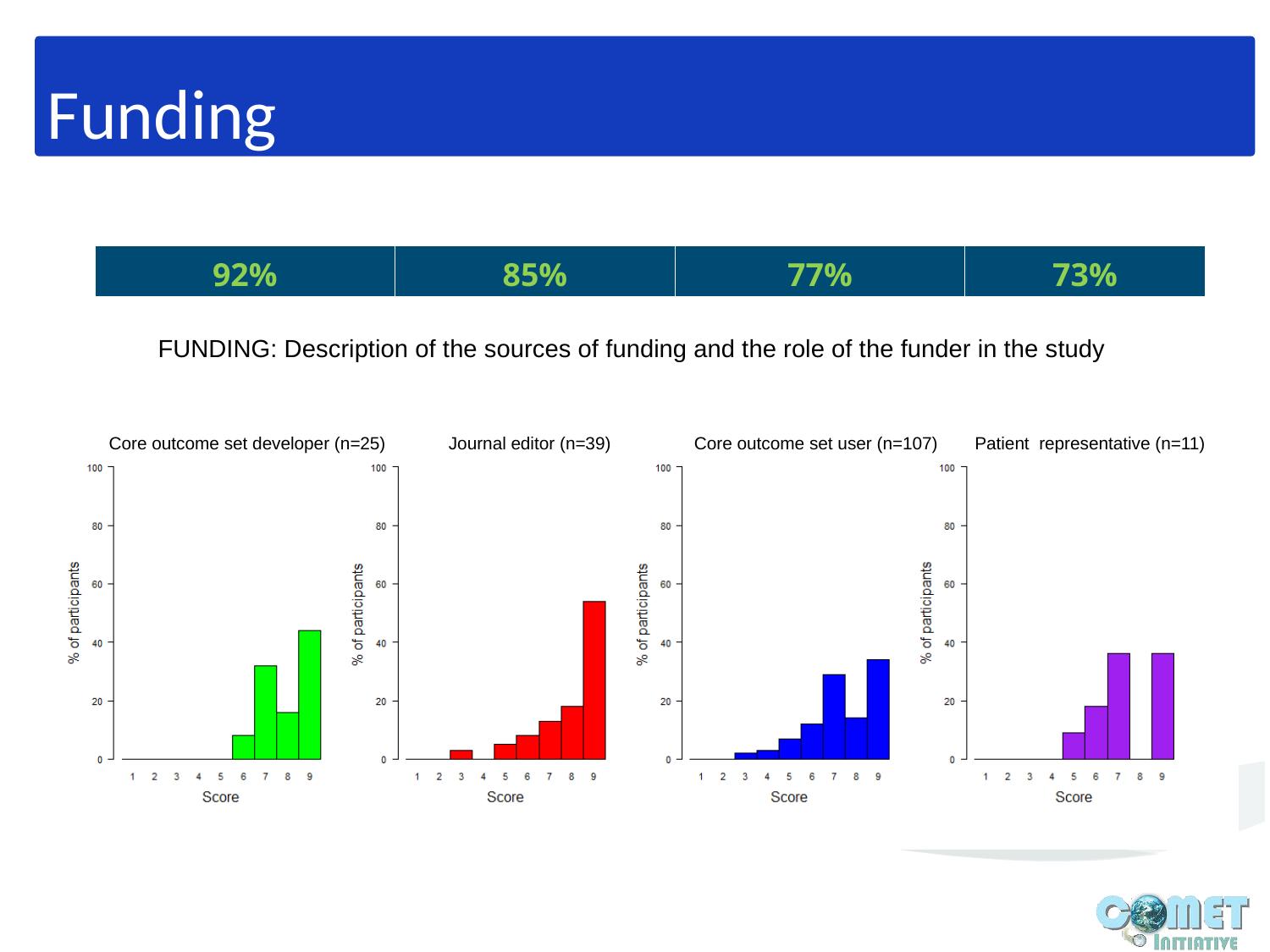

# Funding
| 92% | 85% | 77% | 73% |
| --- | --- | --- | --- |
FUNDING: Description of the sources of funding and the role of the funder in the study
Core outcome set developer (n=25)
Journal editor (n=39)
Core outcome set user (n=107)
Patient representative (n=11)

## Slide 58
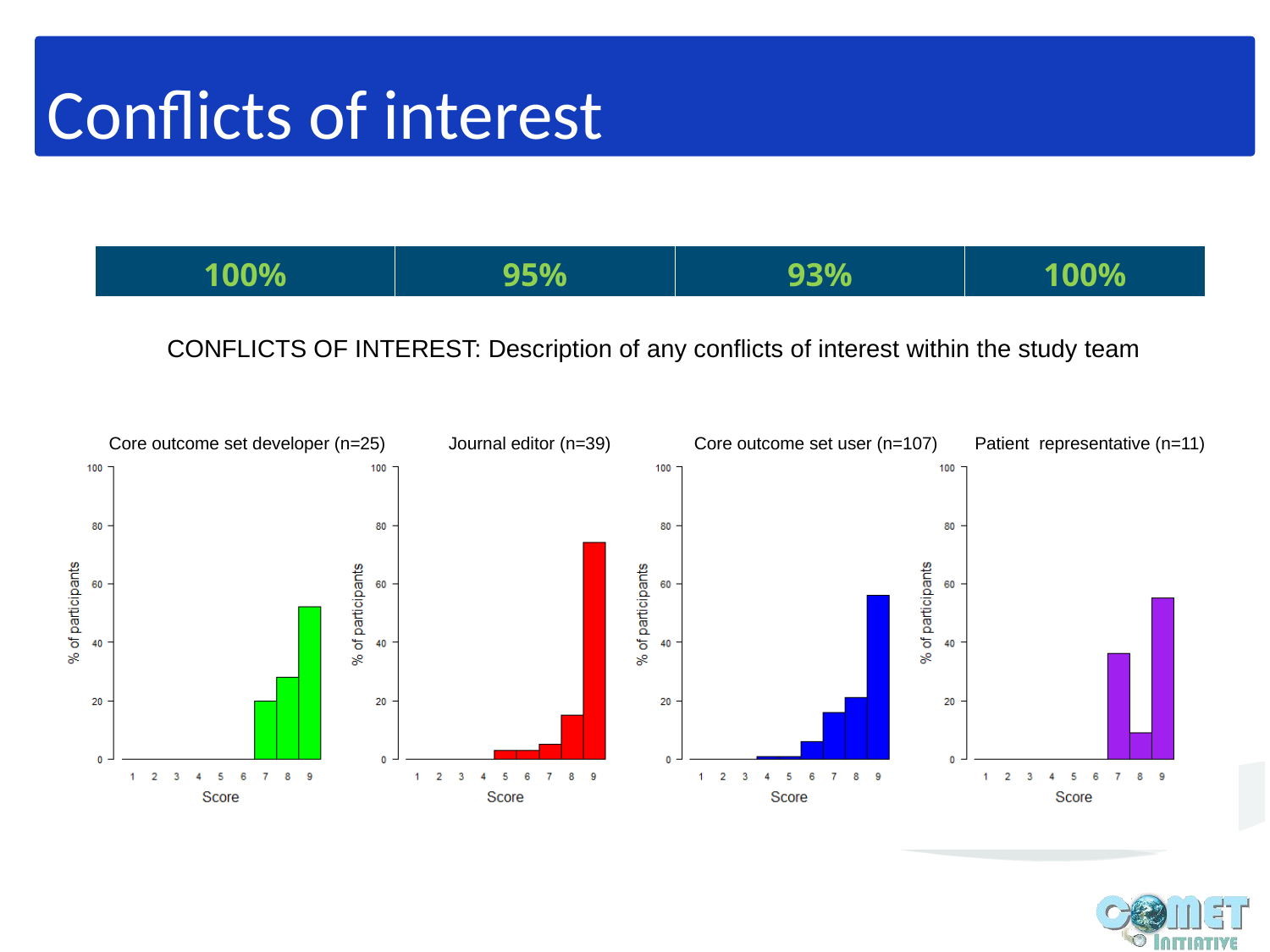

# Conflicts of interest
| 100% | 95% | 93% | 100% |
| --- | --- | --- | --- |
CONFLICTS OF INTEREST: Description of any conflicts of interest within the study team
Core outcome set developer (n=25)
Journal editor (n=39)
Core outcome set user (n=107)
Patient representative (n=11)

## Slide 59
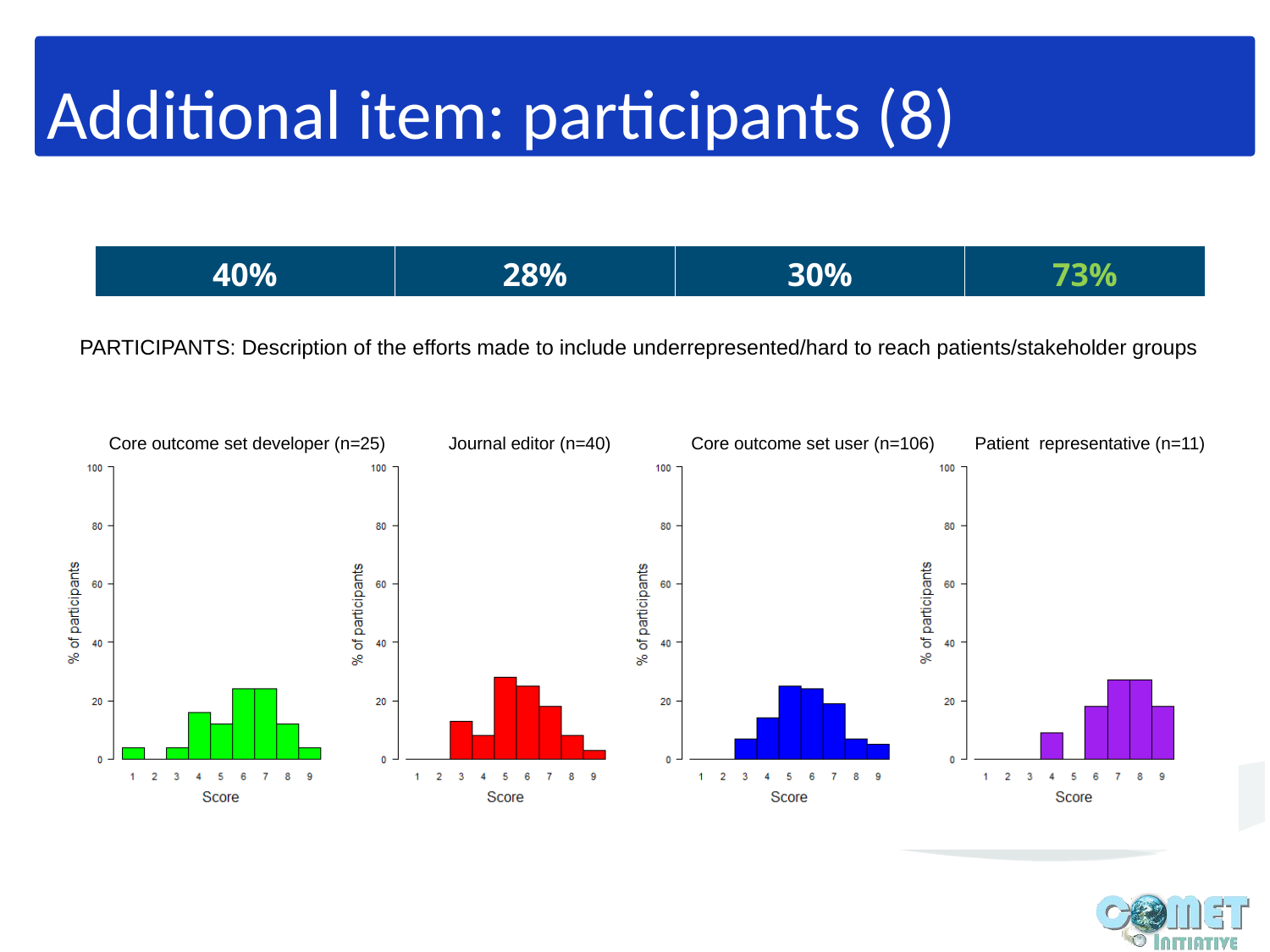

# Additional item: participants (8)
| 40% | 28% | 30% | 73% |
| --- | --- | --- | --- |
PARTICIPANTS: Description of the efforts made to include underrepresented/hard to reach patients/stakeholder groups
Core outcome set developer (n=25)
Journal editor (n=40)
Core outcome set user (n=106)
Patient representative (n=11)

## Slide 60
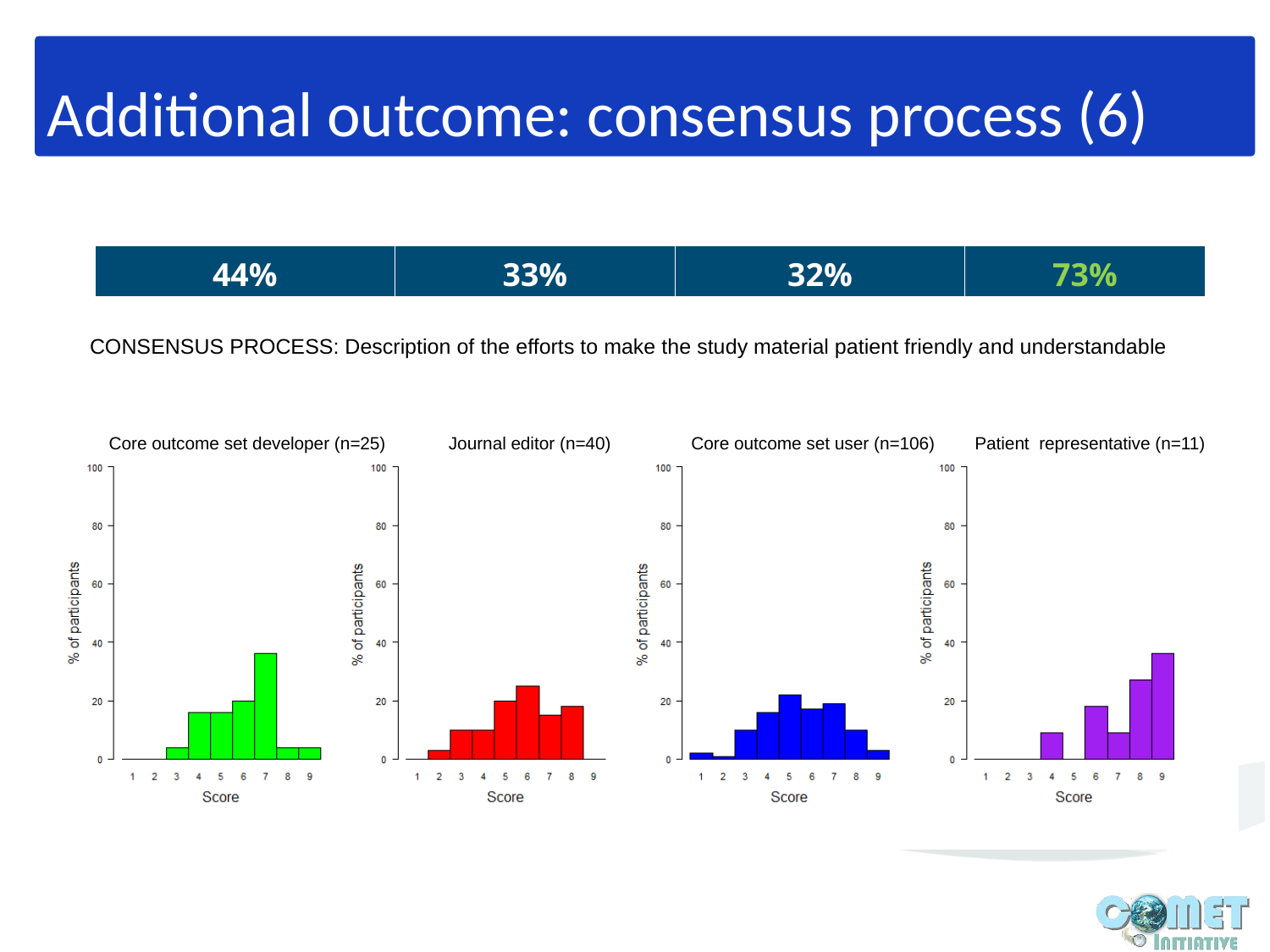

# Additional outcome: consensus process (6)
| 44% | 33% | 32% | 73% |
| --- | --- | --- | --- |
CONSENSUS PROCESS: Description of the efforts to make the study material patient friendly and understandable
Core outcome set developer (n=25)
Journal editor (n=40)
Core outcome set user (n=106)
Patient representative (n=11)

## Slide 61
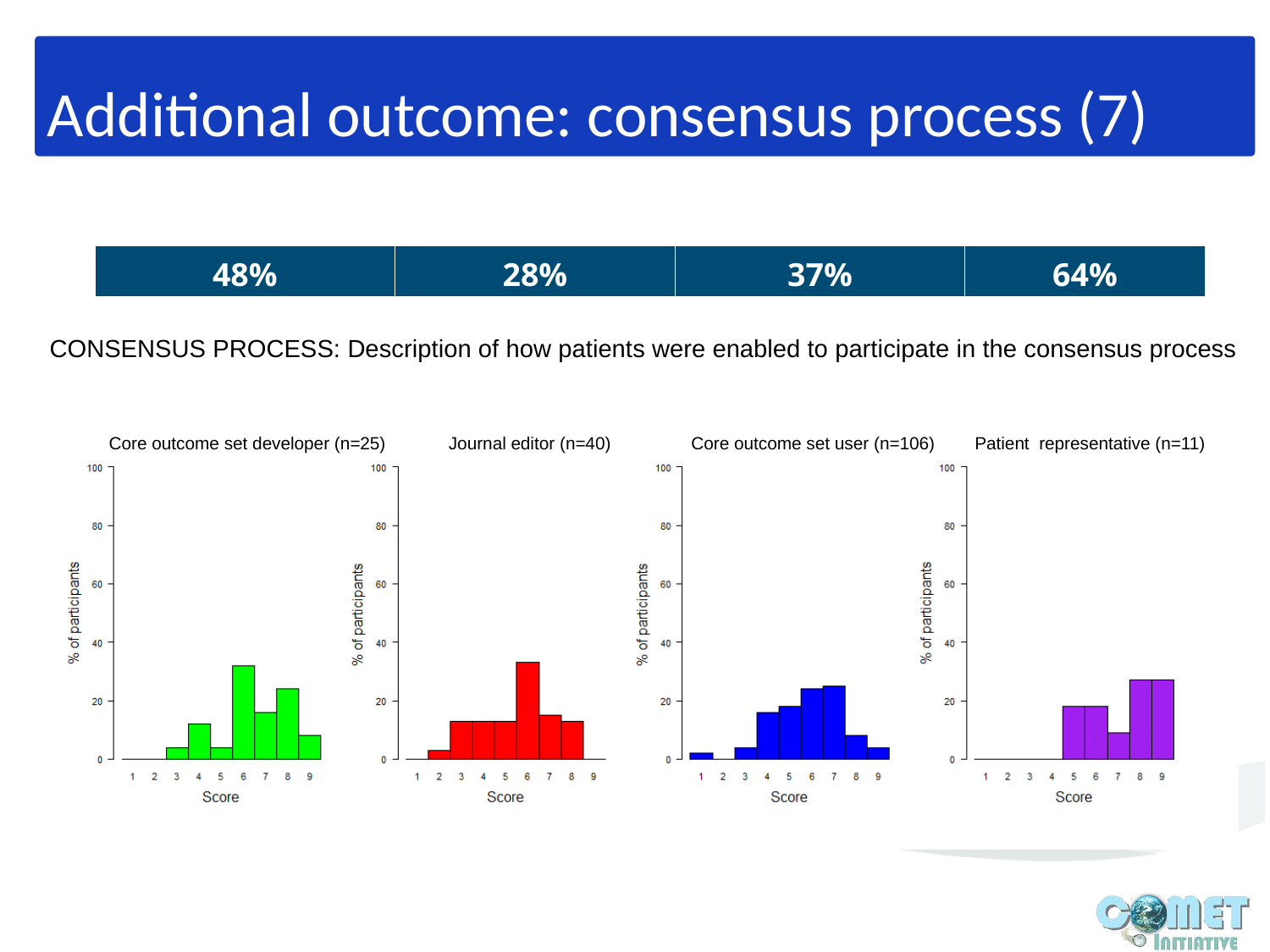

# Additional outcome: consensus process (7)
| 48% | 28% | 37% | 64% |
| --- | --- | --- | --- |
CONSENSUS PROCESS: Description of how patients were enabled to participate in the consensus process
Core outcome set developer (n=25)
Journal editor (n=40)
Core outcome set user (n=106)
Patient representative (n=11)

## Slide 62
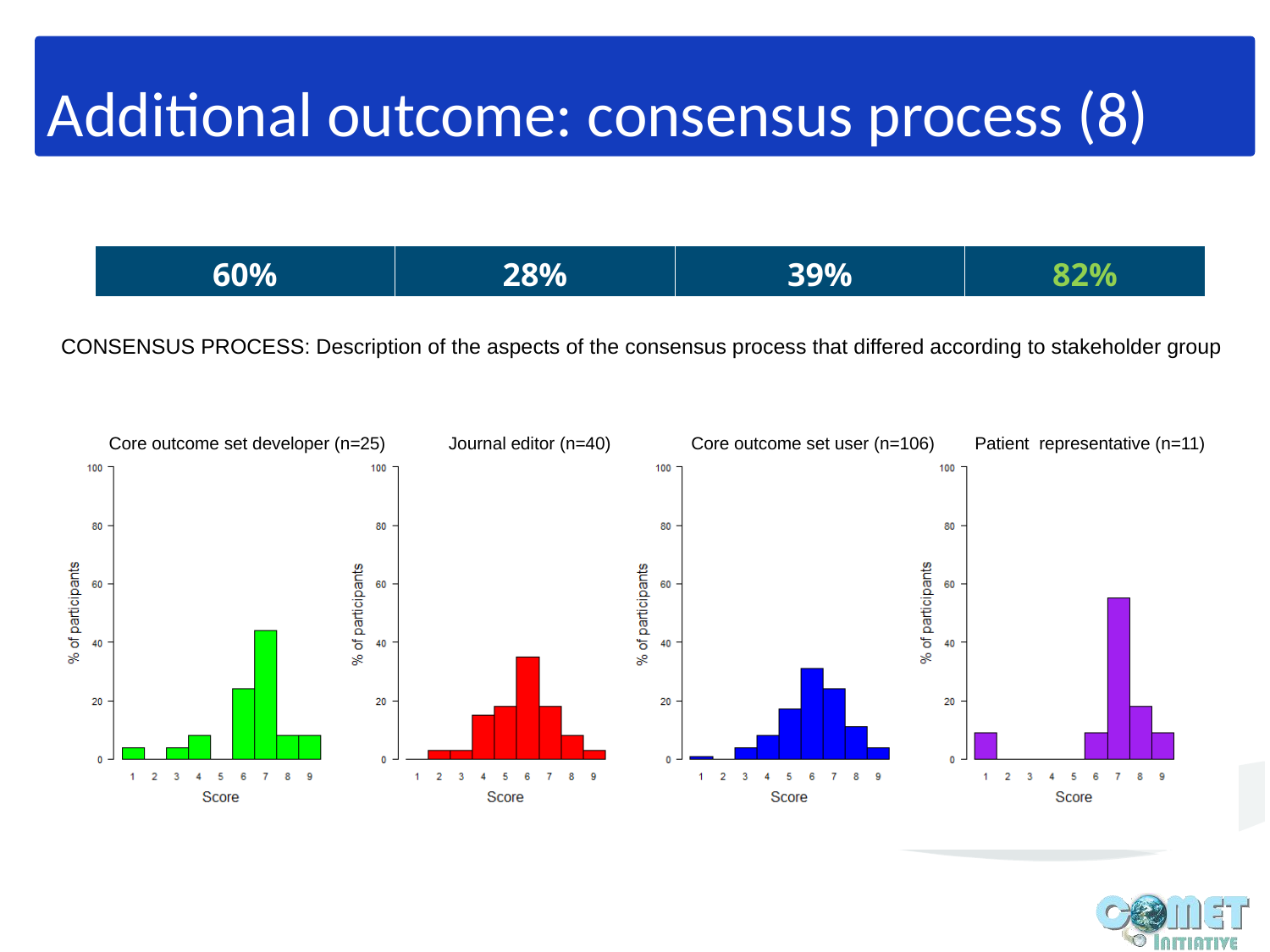

# Additional outcome: consensus process (8)
| 60% | 28% | 39% | 82% |
| --- | --- | --- | --- |
CONSENSUS PROCESS: Description of the aspects of the consensus process that differed according to stakeholder group
Core outcome set developer (n=25)
Journal editor (n=40)
Core outcome set user (n=106)
Patient representative (n=11)

## Slide 63
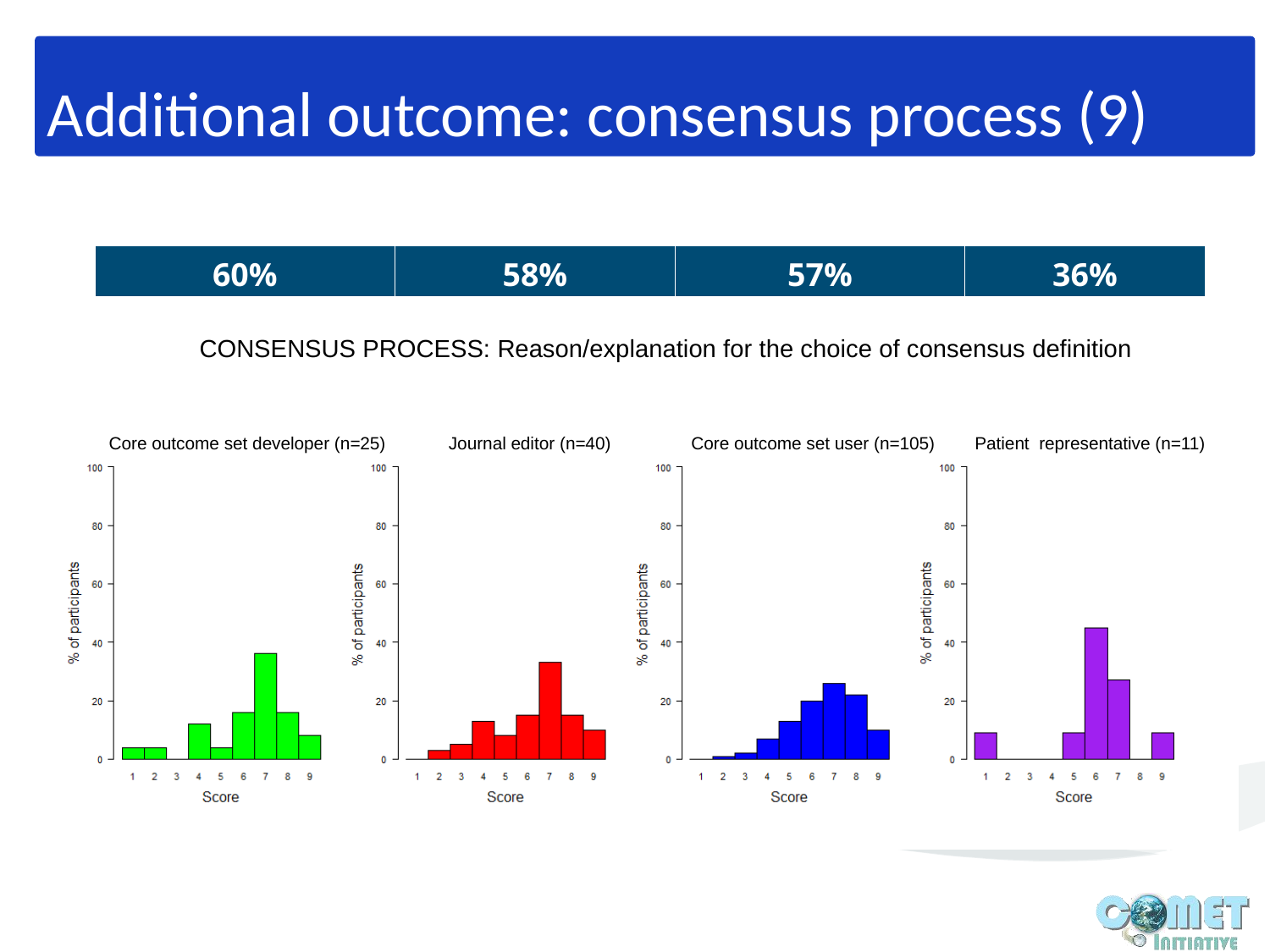

# Additional outcome: consensus process (9)
| 60% | 58% | 57% | 36% |
| --- | --- | --- | --- |
CONSENSUS PROCESS: Reason/explanation for the choice of consensus definition
Core outcome set developer (n=25)
Journal editor (n=40)
Core outcome set user (n=105)
Patient representative (n=11)

## Slide 64
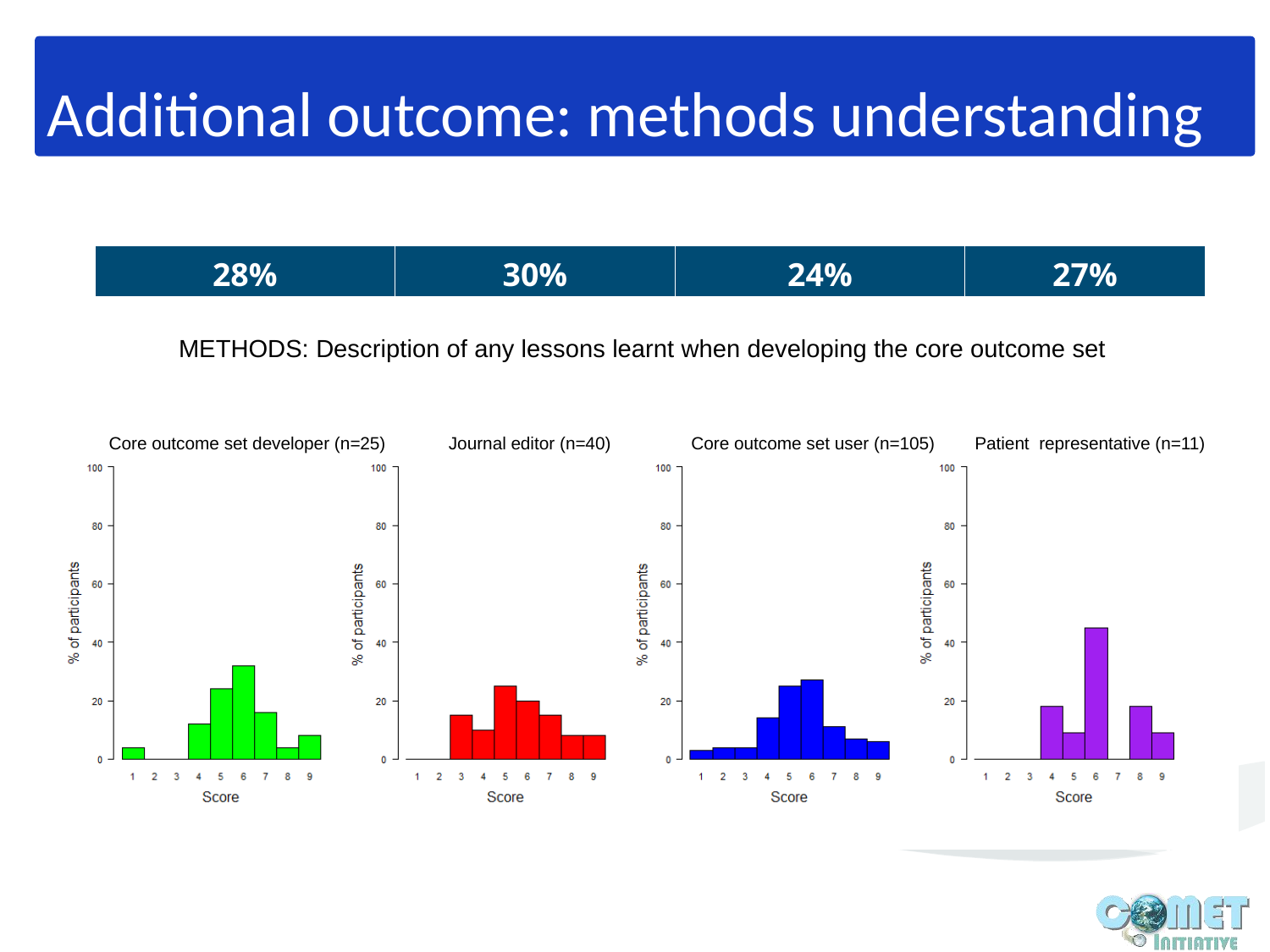

# Additional outcome: methods understanding
| 28% | 30% | 24% | 27% |
| --- | --- | --- | --- |
METHODS: Description of any lessons learnt when developing the core outcome set
Core outcome set developer (n=25)
Journal editor (n=40)
Core outcome set user (n=105)
Patient representative (n=11)

## Slide 65
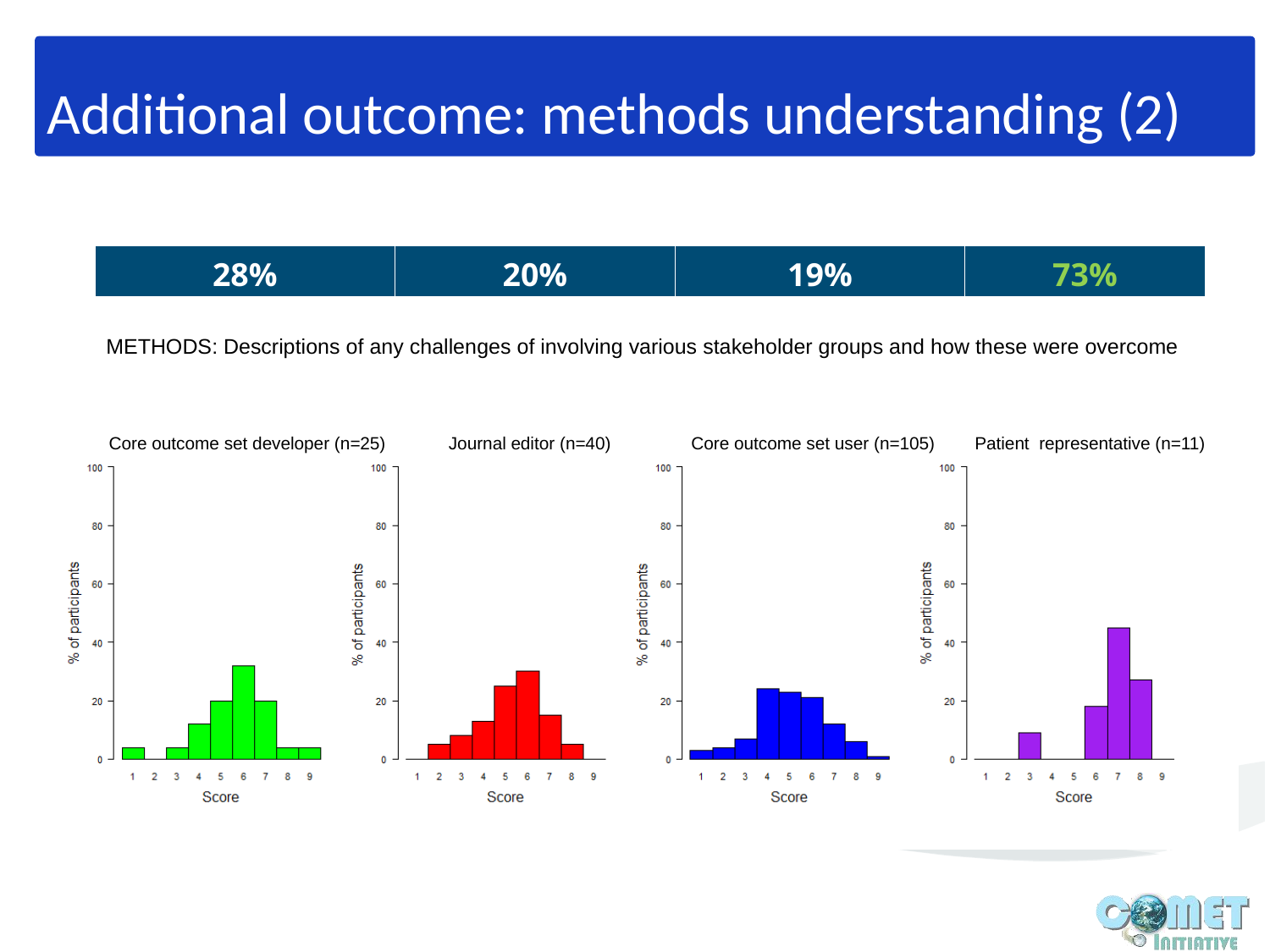

# Additional outcome: methods understanding (2)
| 28% | 20% | 19% | 73% |
| --- | --- | --- | --- |
METHODS: Descriptions of any challenges of involving various stakeholder groups and how these were overcome
Core outcome set developer (n=25)
Journal editor (n=40)
Core outcome set user (n=105)
Patient representative (n=11)

## Slide 66
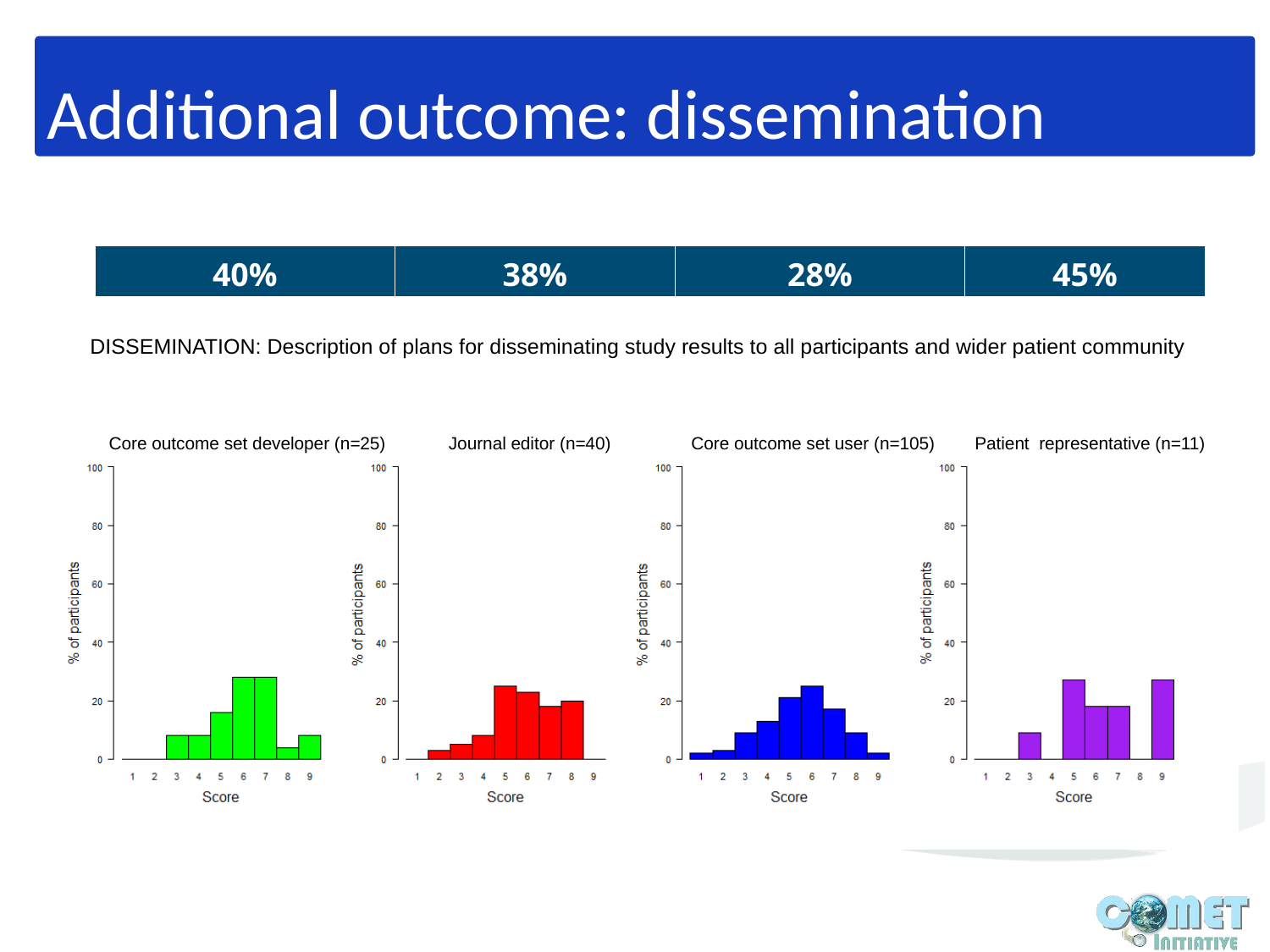

# Additional outcome: dissemination
| 40% | 38% | 28% | 45% |
| --- | --- | --- | --- |
DISSEMINATION: Description of plans for disseminating study results to all participants and wider patient community
Core outcome set developer (n=25)
Journal editor (n=40)
Core outcome set user (n=105)
Patient representative (n=11)

## Slide 67
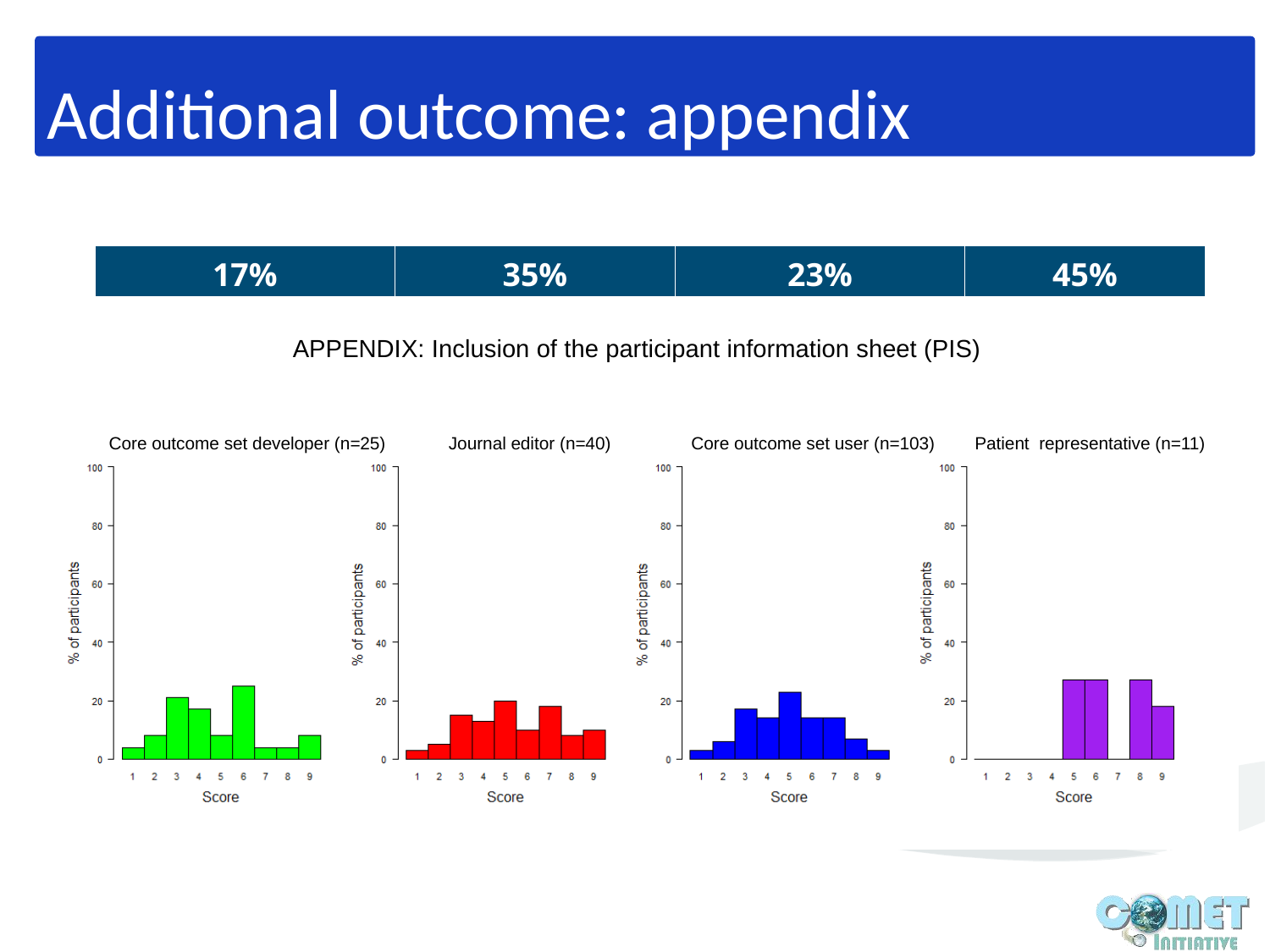

# Additional outcome: appendix
| 17% | 35% | 23% | 45% |
| --- | --- | --- | --- |
APPENDIX: Inclusion of the participant information sheet (PIS)
Core outcome set developer (n=25)
Journal editor (n=40)
Core outcome set user (n=103)
Patient representative (n=11)
