# Supplementary material for: Core Outcome Set–STAndards for Reporting: The COS-STAR Statement
Source: PLoS Med. 2016 Oct 18;13(10):e1002148. doi: 10.1371/journal.pmed.1002148 (PMC5068732; doi:10.1371/journal.pmed.1002148)
Supplement: S1 Explanation and Elaboration — (DOCX) [file pmed.1002148.s005.docx]

**The COS-STAR Statement for Reporting what Outcomes should be Measured from Core Outcome Set Development Studies: Explanation and Elaboration**

**Jamie J Kirkham^1^, Sarah Gorst^1^, Douglas G Altman^2^, Jane M Blazeby^3^, Mike Clarke^4^, Declan Devane^5^, Elizabeth Gargon^1^, David Moher^6^, Jochen Schmitt^7^, Peter Tugwell^8^, Sean Tunis^9^, Paula R Williamson^1*^**

^1^MRC North West Hub for Trials Methodology Research, Department of Biostatistics, University of Liverpool, Liverpool, United Kingdom

^2^Centre for Statistics in Medicine, Nuffield Department of Orthopaedics, Rheumatology & Musculoskeletal Sciences, University of Oxford, United Kingdom

^3^MRC ConDuCT II Hub for Trials Methodology Research, School of Social & Community Medicine, University of Bristol, Bristol, United Kingdom

^4^ Northern Ireland Hub for Trials Methodology Research, Centre for Public Health, Queen's University Belfast, Belfast, United Kingdom

^5^National University of Ireland Galway & HRB Trials Methodology Research Network, Ireland

^6^Ottawa Methods Centre, Ottawa Hospital Research Institute, Ottawa, Canada

^7^Center for Evidence-based Healthcare, Medizinische Fakultät, Technische Univesität Dresden, Dresden, Germany

^8^Institute of Population Health, University of Ottawa, Ottawa, Canada

^9^Center for Medical Technology Policy, Baltimore, USA

**^*^Corresponding Author:**

Professor Paula Williamson

Department of Biostatistics

University of Liverpool

Block F Waterhouse Building,

1-5 Brownlow Street, Liverpool,

L69 3GL

Email: [prw@liv.ac.uk](mailto:prw@liv.ac.uk)

Tel: +44 (0) 151 794 9758

## Abstract

Core outcome sets (COS) can enhance the relevance of research by ensuring outcomes of importance to decision-makers in a particular topic area are measured routinely. Over 200 COS have been developed but the clarity and transparency of these reports is sub-optimal. COS studies will not achieve their goal if reports of COS are not clear, complete and transparent.

In recognition of these issues, an international group that included experienced COS developers, methodologists, journal editors, COS users and patient representatives developed the COS-STAR (Core Outcome Set-STAndards for Reporting) Statement as a reporting guideline for COS studies. The COS-STAR Statement consists of a checklist of 18 items considered essential for transparent and complete reporting in all COS studies.

In this Explanation and Elaboration document, we explain the meaning and rationale for each checklist item. For each item, we include an example of good reporting, and where possible, references to relevant methodological literature. With many COS studies underway, the COS-STAR Statement should be a helpful resource to improve the reporting of COS studies for the benefit of all COS users.

**Introduction**

Core outcome set (COS) studies provide researchers with an agreed standardised set of outcomes that should be measured and reported, as a minimum, in all clinical trials in specific areas of health or health care [1]. COS are also being developed for other settings including routine care. A COS can help to improve healthcare and health by reducing heterogeneity and facilitating data synthesis [1]. This will ultimately help policy makers judge the benefits and harms of health care interventions from the perspectives of all relevant stakeholders in an unbiased way.

Unfortunately, the reporting quality of COS studies is variable, thus diminishing the ability of COS users to assess the relevance to their research. This issue led to the development of the COS-STAR Statement, detailing the minimum set of items that COS developers should report fully and transparently. In this manuscript, we provide some guidance to ensure these recommended COS-STAR reporting items are clearly presented.

**Development of the COS-STAR Statement**

The COS-STAR Statement was developed by a group of COS developers, journal editors, COS users and patient representatives [2] who attended a one-day meeting in 2016. The consensus process was informed by the views of nearly 200 participants across key stakeholder groups in a two-round international, online Delphi survey. The evidence from the survey, consensus meeting discussion and post-meeting correspondence were used to develop the 18-item checklist. After several revisions, the core management group and external panel members approved the checklist [Table 1] and this explanatory paper. Further details regarding the background to and development of the COS-STAR Statement are provided in the publication [2]. This accompanying Explanation and Elaboration document explains the meaning and rationale for each checklist item. Several COS-STAR group participants helped draft specific items for this document and discussed the content to further refine the document, which was circulated and approved by the larger COS-STAR group.

**Scope of COS-STAR Statement**

This work focuses on ways in which authors can ensure the transparent and complete reporting of COS development studies but does not address directly or in a detailed manner the methodologies of stakeholder involvement for developing a COS, for which there is other guidance [1,3-5]. We regard the COS-STAR Statement and this explanatory document as relevant to all COS developers wishing to report their studies, regardless of the methodology used to develop the COS and the various participant groups who may have been involved in selecting outcomes.

**How to use this paper**

We developed this Explanation and Elaboration document using a similar format for other reporting guidelines, such as the PRISMA Statement (Preferred Reporting Items for Systematic Reviews and Meta-Analyses) [6]. To maximise the benefit of this document, we would encourage authors to read it in conjunction with the COS-STAR Statement [2].

We present each checklist item and explain the rationale for and importance of including the item in the report of a COS study. We also provide relevant evidence from the literature, where available. We supplement each reporting item with published examples of good reporting practice that covers key aspects that should be considered when addressing each reporting item. Different terminologies for *outcomes* in COS studies have been used, for example *domains* [7], *endpoints* [8] and *symptoms* [9]. Throughout this paper, we use the term *outcome,* but other terms are used in the examples provided. We would encourage COS study authors to provide definitions of the terms they use to avoid misunderstanding.

Unlike the literature for previous guidelines, the pool of published COS studies is limited to just over 200 [10], compared to more than half a million reports of randomised controlled trials and more than 40,000 systematic reviews [11] that could have been used as examples for the CONSORT (Consolidated Standards of Reporting Trials) [12] and PRISMA [13] guidelines, respectively. For this reason, some of the examples were amended from what had been published in a COS report. We identify where edits were made to the published examples and add in any necessary information such as expanding abbreviations or explaining the context in further detail, if this was considered to be important for understanding of the example. No systematic search was carried out to identify any of the examples, but they were identified by a variety of methods by the authors and the wider COS-STAR group.

**The COS-STAR Checklist**

**TITLE and ABSTRACT**

**Item 1a: TITLE:** Identify in the title that the paper reports the development of a COS

**Explanation:** Authors should identify their report as a COS development study. Although sensitive search strategies [14] have been developed and databases have been identified [15] to search for COS development studies, inclusion of the term “core outcome set” in the title may improve indexing and identification. However, there may be some situations where the focus of the manuscript is on general design issues for a study for which recommended outcomes is just one component [16-20]. In this specific situation, inclusion of ‘core outcome set’ in the title may not be entirely appropriate, but we strongly recommend that the study is registered in a COS database (see item 4) for identification purposes and that the term ‘core outcome set’ is used in the abstract.

**Example:** “Developing a core outcome set for hip fracture trials” [21]

**Example:** “Assessment of chronic post-surgical pain after knee replacement: Development of a core outcome set” [22]

**Item 1b: ABSTRACT:** Provide a structured summary

**Explanation:** An abstract provides the key information that enables readers to understand the scope, the methods and the findings of a COS development project and may be the only part of the report that a reader may have access to from a bibliographic database, for example, if the article was not published in an open-access journal. The abstract should therefore present a clear and balanced assessment (albeit brief) of the main text of the report and should contain the key information, such as a list of the recommended outcomes in the COS.

Journals will often differ in their required abstract format and word length and while we do not favour a specific format over another, a structured abstract can often provide readers with more complete information, and can facilitate finding the key information more easily [6].

A highly structured abstract of a COS development study could include the following headings: Background (or Context), Objectives (or Purpose), Data Sources, Stakeholder Eligibility Criteria, Methods, Results (Core Outcome Set), Limitations and Conclusions. In a simpler abstract structure, the above headings could be collapsed (e.g. Background and Objectives) or some of the headings such as Limitations could be omitted.

Taking the highly structured abstract mentioned above, authors use the *Background* heading to set the context for the readers and explain the importance and the rationale for developing a COS in the area. Under the *Objectives* heading, they describe the purpose of the study and define the scope [see item 3] of the COS. Under the *Data Sources* heading, they summarise how an original list of outcomes for potential inclusion in the COS was identified. *Stakeholder Eligibility Criteria* describes who was eligible to be involved in the consensus process and how they were identified. The *Methods* section describes the methods that were used to reach a consensus. Under the *Results* heading, the recommended set of core outcomes should be reported. Under the *Limitations* heading, authors might describe any important weaknesses in the COS development process (such as poor stakeholder representativeness) or in the relevance of the COS to certain settings. The authors should then provide clear and balanced *Conclusions* that are closely linked to the objectives and findings of the COS development process.

**Example:** (adapted [23], abstract length < 300 words)

**Background:** Approximately 75% of children with cleft palate (CP) have Otitis Media with Effusion (OME) histories. Evidence for the effective management of OME in these children is lacking. The inconsistency in outcome measurement in previous studies has led to a call for the development of a Core Outcome Set (COS).

**Objectives:** To develop a core outcome set for use in the management of OME in children with CP.

**Data sources:** A list of outcomes measured in previous research was identified through reviewing the literature.

**Stakeholder eligibility criteria:** Ear, Nose and Throat (ENT) surgeons, audiologists, cleft surgeons, speech and language therapists, specialist cleft nurses, psychologists affiliated to a UK Cleft Centre; parents and children identified from two cleft centres in the UK, the Cleft Lip and Palate Association (CLAPA) children and young person’s council (CYPC), and a local CLAPA ‘Happy Faces’ group.

**Methods:** A three round Delphi survey of health care professionals, a survey and semi-structured interviews with parents and children, and a consensus meeting of key stakeholders.

**Results:** We recommend the following eleven core outcomes that apply to OME in children with CP: hearing, chronic otitis media, OME, receptive language skills, speech development, psychosocial development, acute otitis media, cholesteatoma, side effects of treatment, listening skills, otalgia.

**Limitations:** Whilst clinical stakeholder representation was good the number of parents and children completing the online survey and attending the face to face consensus meeting was lower than expected. The opinions were sought from UK centres only.

**Conclusions:** We have produced a recommendation about the outcomes that should be measured, as a minimum, in studies of the management of OME in children with CP. The development process included input from key stakeholders and used novel methodology to integrate the opinion of healthcare professionals, parents and children.

**INTRODUCTION**

**Item 2a: BACKGROUND:** Describe the background and explain the rationale for developing the COS

**Explanation:** Readers need to understand the rationale behind the COS development study in the context of the health area and identify the reasons why a COS is needed. Lack of consensus between experts on what outcomes to measure can lead to inconsistencies in the reporting of outcomes, heterogeneity of outcome measurements and outcome reporting bias [24], all of which are valid reasons for using a consensus-based approach to identify a COS. If a COS already exists in the area (see item 4), then the authors should tell the readers why they developed a new COS. For example, while the new COS is in the same area as an existing COS, the scope (see item 3) may be different in terms of the stage of the disease for example. Alternatively, there may be known limitations or quality concerns associated with an existing COS. As an example, key stakeholders (e.g. patients) may not have been involved in the development process, suggesting perhaps that outcomes of importance to those stakeholders may be missing.

As an ideal background or introduction that describes the context for the COS, readers might consider the following. First, the authors might want to describe known problems with outcome selection and measurement in relation to the condition. Second, authors might want to describe the importance of a COS from different stakeholder perspectives (e.g. health care professionals, patients). Third, authors might want to add in the study aims and discuss the extent to which the limitations of any existing COS in the area will be overcome in this current study.

**Example:** Study Background: recommended patient-reported core set of symptoms [outcomes] to measure in adult cancer treatment trials

“The lack of an agreed-upon core set of symptoms to be collected in adult oncology treatment trials reflects the heterogeneity of cancer types and effects of treatments on patients’ lives. For example, treatments for localized prostate cancers are associated with diarrhea…, whereas treatments for head and neck cancers are associated with mucositis…. However, several symptoms, including fatigue… are commonly experienced across different cancer sites and treatment modalities. Systematic assessment of a core symptom set across all trials where patient-reported endpoints are included would 1) encourage the inclusion of the patient’s perspective consistently across clinical trials and facilitate comparative effectiveness research; 2) enhance our understanding of the impact of cancer and its treatment on patients’ lives, which in turn may help identify effective treatment and supportive care strategies; and 3) enhance data harmonization across trials, permitting integrated data analysis and meta-analysis. Ultimately, this would lead to more efficient and robust research approaches.” [9]

**Item 2b: OBJECTIVES:** Describe the specific objectives with reference to developing a COS

**Explanation:** The objectives are the questions that the COS development study was designed to answer. In the context of COS development studies, the objectives will often refer to a consensus based approach for establishing important outcomes for a particular health condition.

**Example:** “Specific objectives of the COS development in the MOMENT [Management of Otitis Media with Effusion in childreN with cleft palaTe] study were: to identify outcomes that had been previously reported in studies of the treatment of OME [Otitis Media with Effusion]; to prioritise outcomes from the perspective of health professionals; to prioritise outcomes from the perspective of patients who can express their views, and parents; and to integrate the opinions of patients, parents and health professionals into a combined COS.” [23]

**Example:** “To develop a consensus based set of core domains for outcome studies in psoriatic arthritis.” [7]

**Example:** “In this article, we report the recommended core set of symptoms and HRQOL [health related quality of life] domains agreed upon by the H&N [head and neck] working group subcommittee that should be assessed in future clinical trials for H&N cancer patients.” [25]

**Item 3: SCOPE**

**Explanation:** The scope of a COS refers to the specific area of health or health care to which the COS is to be applied [1]. The scope should be described in terms of the health condition and target population, interventions that the COS is to be applicable to and the setting for which the COS is to be applied. The scope therefore covers the first three elements of the PICO (Population, Intervention, Comparator, Outcomes) structure for a clinical trial.

Defining the scope of the COS can be challenging. Ensuring it is clear, however, is recommended from the outset. This will assist outcome prioritisation and discussions in consensus meetings. It will likely reduce discussions at a late stage and possibly the need to repeat earlier work (it there was ambiguity about the scope). This will also help potential users decide on its relevance to their work.

**Item 3a: SCOPE (Health condition and population):** Describe the *health condition(s)* and *population(s)* covered by the COS

The health condition and population covered by the COS should be adequately described. For example in cancer, the COS may cover cancer generally [26] or it may be specific to a particular cancer type such as head and neck cancer [27]. Similarly, a COS may be developed for all patients but it could also be developed for a specific subset of the population in mind, such as localised prostate cancer patients [28,29] or advanced prostate cancer patients [30], women [31] or children [32].

**Example:** “The aim of this study, therefore, was to develop and pilot a method by which to identify outcomes of particular relevance when evaluating the effects of regular therapies for *chronic childhood asthma* [health condition]…of *children* with asthma… *younger than 18* years [population].” [32]

**Example:** “The Standard Set was designed around *men with clinical American Joint Committee of Cancer (AJCC) stages T1–T4* [population] *localized prostate* *cancer* [health condition] treated with curative intent or followed with active surveillance.” [29]

**Item 3b: SCOPE (Interventions):** Describe the *intervention(s)* covered by the COS

The types of interventions covered by the COS should be adequately described.  A COS may be developed to apply to all interventions for a particular condition or for a specific intervention, but details about what is actually covered should be provided.  As an example, if the COS is only relevant to specific classes of intervention, such as surgery or specific drug types (e.g. biologics), this detail should be presented.

**Example:** “The scope of this COS includes clinical effectiveness trials (rather than trials of treatment efficacy) of all surgical interventions for cancer of the colon and rectum. Excluded were oncological interventions.” [33]

**Example:** Study Background: to identify a set of critical and important outcomes (core outcome set) for the evaluation of preventive interventions for preterm birth in asymptomatic pregnant women

“Preventive treatment [interventions] of preterm birth was defined as one started before any symptoms of preterm labor were present. This preventive strategy could be *pharmacologic* (e.g., progesterone, marine oils, probiotics) or *non-pharmacologic* (e.g., cerclage, pessary, lifestyle interventions, and alternative therapies).” [34]

**Item 3c: SCOPE (Settings):** Describe the *setting(s)* in which the COS is to be applied

The focus of many COS development projects is about application in effectiveness trials.  However COS are applicable in other settings such as for use in other research designs, systematic reviews [35], routine care or audit; and a recent study is developing a COS for both clinical and research purposes [36].  It is important for COS developers to describe the COS setting as outcomes may be different for different settings. Defining the setting is also important as there are COS that have been developed for clinical trials that are now being endorsed by Cochrane Review Groups; an example is the **C**ochrane **S**kin **G**roup - **Co**re **Ou**tcome **S**et **In**itiative (CSG-COUSIN) [37].

**Example:** “The main goal of this WG [working group] is to develop a core outcome set for *clinical trials* in acute diarrhoea.” [38]

**Example:** “…to reach consensus among key stakeholders including cancer survivors on the relevance, acceptability, and feasibility of a core outcome set for collection in *routine clinical care*.” [39]

**Example:** “To aid international collaboration, it is essential to have a core set of data that all researchers and clinicians collect in a standardised way for *clinical purposes* and for *research*.” [36]

**Further example of Scope (covering items 3a, 3b and 3c)**

**Example:** “The Steering Committee recommended that this COS should apply to measuring efficacy or effectiveness [research design] of health interventions in *clinical trials* [setting] for *patients with NSLBP* (non-specific low back pain) [population], … *All interventions* [intervention] for NSLBP [health condition] are targeted by this COS, regardless of type, setting, or mode of administration.” [40]

**Example:** “Our objective was to develop robust, valid composite maternal and neonatal outcome measures for clinical trials [setting] evaluating *all* interventions [intervention] in women [population] with late-onset mild and moderate pre-eclampsia [health condition].” [31]

**METHODS**

**Item 4: PROTOCOL/REGISTRY ENTRY:** Indicate where the COS development protocol can be accessed, if available and/or the study registration details

**Explanation:** There are potential sources of bias in the COS development process. One of these relates to how and why the consensus criteria were defined a priori, to avoid changing the criteria after the results have been analysed. To help reduce these biases and to improve transparency, we recommend that a protocol is developed before the start of the study and made publically available to help restrict the likelihood of biased post-hoc decisions in COS development [41]. A growing number of COS study protocols are being published [e.g., 28, 36, 42-43].

The registration of COS is one way to reduce waste in research by helping to avoid unnecessary duplication of effort and also makes it easier for researchers to identify a COS if one already exists [44]. Registration of COS studies is possible via the COMET database which is freely available on the internet (<http://www.cometinitiative.org/contactus/submitnewstudy>).

**Example:** “The study protocol for this work [core outcome set development: otitis media with effusion in children with cleft palate]… has been previously published.” [23]

**Example:** Database Registration: “COMET Initiative, http:// www.comet-initiative.org/studies/details/603, Registration Number: 603.” [34]

**Item 5: PARTICIPANTS:** Describe the rationale for stakeholder groups involved in the COS development process, eligibility criteria for participants from each group and a description of how the individuals involved were identified

**Explanation:** Knowledge of the processes for selection of the types of stakeholder groups and individuals from within them is essential for appraising the COS. Provision of these details is therefore recommended wherever possible. Eligibility criteria may include the level of experience or specific knowledge of the condition/intervention. Describing how individuals of stakeholder groups were identified is important as it indicates if the list of those targeted is inclusive.

**Example:** Study Background: two-round Delphi survey looking to develop a COS for oesophageal cancer surgery

“The questionnaire developed in phase 1 was sent to key stakeholders, including upper gastrointestinal and thoracic consultant surgeons and senior surgical trainees, clinical nurse specialists and patients who were awaiting or who had undergone surgery for oesophageal cancer. Surgeons and nurses were identified through a meeting of the Association of Upper

Gastrointestinal Surgeons of Great Britain and Ireland, and by personal knowledge of surgeons in oesophageal cancer surgery centres. Patients were recruited from Bristol and Birmingham in the UK, and from Amsterdam in the Netherlands.” [45]

**Example:** Study Background: two-round Delphi survey looking to identify important outcomes for acupuncture and moxibustion to promote cephalic version for women with a breech presentation

“Sixteen English speaking international, Australian and New Zealand acupuncturists working in the area of pregnancy were invited to participate in the study. The criteria used to identify participants included published on acupuncture or moxibustion and pregnancy, or currently undertaking research on acupuncture/moxibustion in pregnancy, experienced teachers of this TCM [Traditional Chinese Medicine] technique, and/or a minimum of five years’ experience of using acupuncture in pregnancy.” [18]

**Example:** Study Background: to develop a core outcome set for research and audit studies in reconstructive breast surgery including patients, breast and plastic surgeons, specialist nurses and psychologists

“Key stakeholders were defined as individuals who may be involved in decision-making for RBS [reconstructive breast surgery] and would have an in-depth understanding of which outcomes should be measured in research and audit studies in this area. These were identified by the BRAVO [Breast Reconstruction And Valid Outcomes] Steering Group as patients, breast and plastic surgeons, clinical nurse specialists and psychologists…Patients were purposively sampled from three centres (Bristol, Liverpool and Glasgow). All women who had undergone RBS using expander/implants, latissimus dorsi or abdominal flaps as either immediate or delayed procedures, or who had undergone therapeutic mammoplasty, defined as reduction pattern wide local excision and contralateral symmetrization, within 5 years of the start of the study were eligible to participate…Professionals were recruited purposively from breast and plastic surgical units across the UK.” [46]

**Item 6a: INFORMATION SOURCES:** Describe the information sources used to identify an initial list of outcomes

**Explanation:** Generating an initial list of candidate outcomes for potential inclusion is an important component of COS development. Reporting this list enables the reader to determine if outcomes considered in the consensus process are likely to be representative of the perspectives of relevant stakeholders. Authors should describe the different information sources used to generate the initial list of outcomes. For example, a list of outcomes from published clinical trials may be supplemented by undertaking interviews with patients or obtaining input from an advisory group whose membership reflects the key stakeholders. Authors should indicate who provided the information where possible. Outcomes reported from a review of trials may be mostly reflective of outcomes from a health professional’s perspective while interviews with patients might reflect outcomes that are more important to them.

**Example:** “The Steering Committee took responsibility for drawing a list of potential core domains that was used in the Delphi study. This list resulted from a search of outcome domains measured in clinical trials included in five recent systematic reviews (one of which not published yet) with addition of the (sub) domains included in the comprehensive International Classification of Functioning (ICF) core set for LBP [lower back pain], and in a conceptual model developed to characterize the burden of LBP. This conceptual model and the ICF core set were adopted to account for the patients’ perspective in this early phase.” [40]

**Example:** “A list of outcomes previously reported in studies of the treatment of OME was generated by updating a 2009 systematic review… All outcomes and domains were discussed with members of the Study Advisory Group (SAG) prior to being finalised… Semi-structured interviews with parents and children gave in-depth information on outcomes of importance for these groups.” [23]

**Item 6b: INFORMATION SOURCES:** Describe how outcomes were dropped/combined, with reasons (if applicable)

**Explanation:** COS developers may drop or combine outcomes from the initial list if outcomes are considered to be overlapping in content or repetitious (Item 6a). It is necessary to provide details of why and how this was done and by whom. A description of these details allows full transparency for readers.

**Example:** “An initial instrument was developed containing 299 outcomes identified from the aforementioned systematic review of randomized trials comparing 2 or more models of maternity care (to be reported separately). Outcome measures addressing similar dimensions or events were discussed by the team and collapsed where possible. For example, various modes of delivery/birth were presented as ‘‘mode of birth (e.g., spontaneous vaginal, forceps, vaginal breech, caesarean section, vacuum extraction).’’ This pilot tool was tested for clarity, with a sample of 12 participants, including 3 maternity care consumers, and subsequently refined.” [47]

**Example:** “Because we expected a large diversity in reported outcomes [from the systematic review of outcomes], we grouped similar outcomes into overarching outcome categories by a small-group consensus process. The group of experts consisted of 6 doctors in dental surgery specialized in pediatric dentistry, including 3 clinical research investigators. First, the group identified outcomes that were identical despite different terms used across trials. Second, different but close outcomes (i.e., outcomes that could be compared across studies or combined in a meta-analysis) were grouped together into outcome domains. Finally, the group, with consensus, determined several outcome categories and produced a reduced-outcome inventory.” [48]

**Example:** Study Background: pain outcomes were identified for inclusion into a Delphi study through three sources: a systematic review, structured interviews and four focus groups

“Data about the assessment of pain were extracted from these sources and coded into pain features. Coding was performed in duplicate by two researchers and pain features were then refined and modified through further discussion and review. The resulting 68 pain features were further refined through discussion with a patient and public involvement group specializing in musculoskeletal research [Patient Experience Partnership in Research; PEP-R] and the Project Steering Committee.” [22]

**Item 7: CONSENSUS PROCESS:** Describe how the consensus process was undertaken

**Explanation:** There is currently wide variation in the approaches used to achieve consensus [14] and research to identify optimal methods for developing COS is ongoing. Methods include the Delphi technique [38, 49], nominal group technique [21,50], consensus development conference [51] and semi-structured group discussion [8] amongst others. Many studies have used a combination of methods to reach consensus, for example, Ruperto (2003) used the Delphi approach followed by the nominal group technique [52]. Therefore it is important for authors to describe the consensus process methodology in full. For more complex designs, where different stakeholder groups engage in the consensus process via different approaches, a diagram or figure may help describe the consensus process.

**Example:** “We integrated key aspects of the modified nominal group technique (NGT)…with an approach towards achieving consensus…from a group of experts through highly structured, facilitator-led discussion.” [21]

**Example:** “The group’s process included 1) a systematic literature review to determine the prevalence and severity of symptoms, 2) a multistakeholder meeting sponsored by the NCI [National Cancer Institute] to review the evidence and build consensus, and 3) a postmeeting expert panel synthesis of findings to finalize recommendations.” [53]

**Example:** “We report the results of a three-component process: medical expert Delphi exercise, patient perspective investigations and a combined medical expert and patient participant nominal group technique (NGT) meeting leading to identification of preliminary core sets of domains with corresponding instruments that are clinically meaningful and feasible in the context of

a 1-year multi-centre RCT for each CTD-ILD [connective tissue disease related interstitial lung] and IPF [idiopathic pulmonary fibrosis].” [54]

**Example:** “Methods used…systematic review, health professional Delphi survey, semi-structured interviews [with parents and children]…final study consensus meeting... An online survey of parents and children…. An overview of the COS development process is provided in [Figure 1].” [23]

**Figure 1 | Overview of the COS development process**

[taken from [23] (Figure 1)]

**
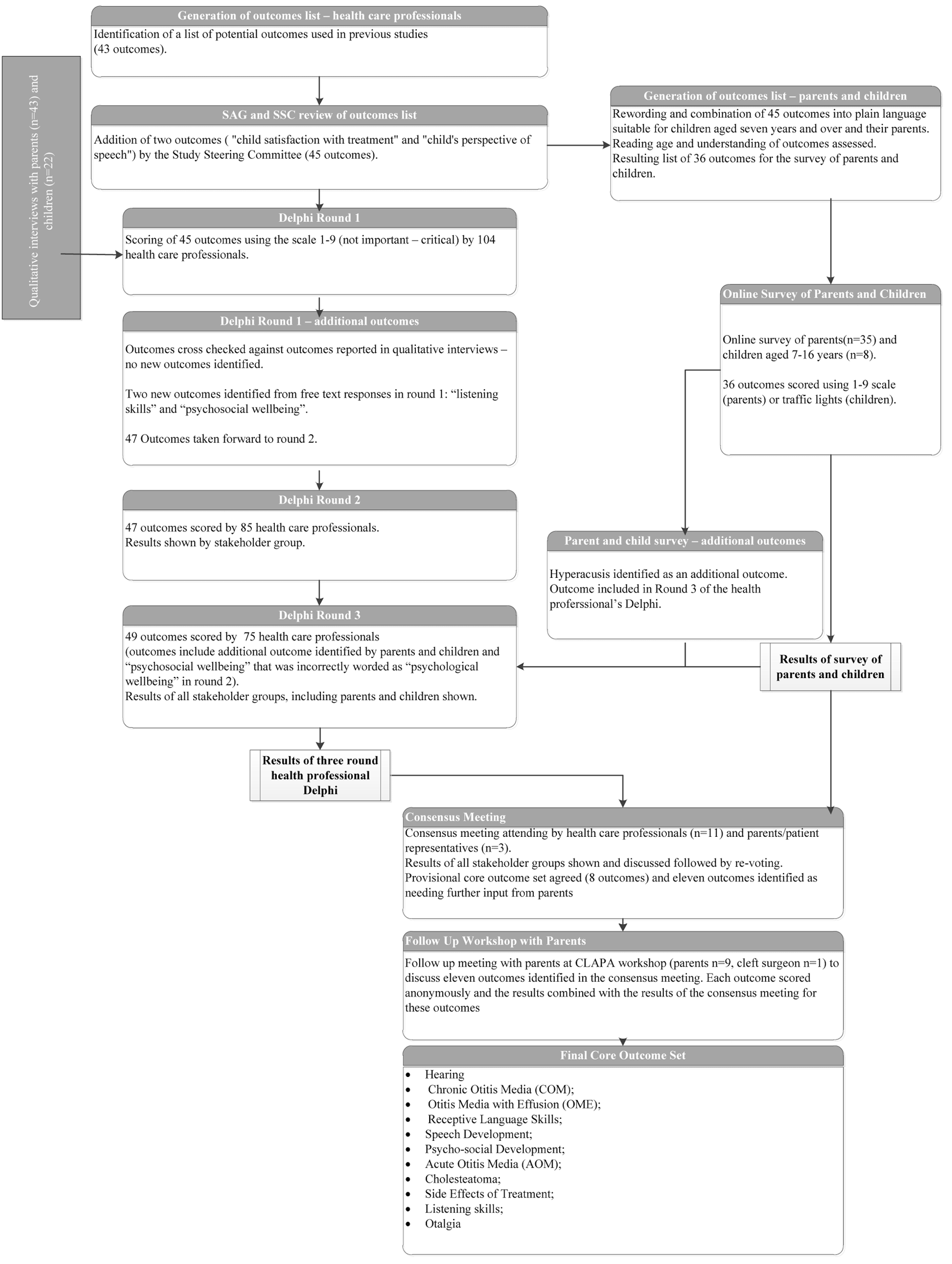
**

**Item 8: OUTCOME SCORING:** Describe how outcomes were scored and scores summarised

**Explanation:** A variety of different scoring systems have been used in COS studies to rate the importance of outcomes and therefore it is important to describe how this was done. The majority of studies have used Likert scales [23, 45-46, 55-56] although others have used ranking of outcomes [52, 57] and allocation of points [58-59]. Authors should tell the readers how the scoring method used signifies the level of importance of an outcome and if appropriate, describe any differences in the scoring method used for different stakeholder groups.

In many consensus exercises, the results are often summarised and fed back to participants to re-consider. The results may also be summarised for use at a consensus meeting. The method of summarising and feeding back the results should be described.

**Example:** “…health professionals were asked to score a list of outcomes using the Grading of Recommendations, Assessment, Development and Evaluations scale [GRADE] of 1 to 9, with 1 to 3 labelled ‘not important’, 4 to 6 labelled ‘important but not critical’ and 7 to 9 labelled ‘critical’… The labels of the 1–9 scale were modified for parents whilst children, under the recommendation of the CYPC [children and young person’s council], scored each outcome using a traffic light system where the scores 1–3 were represented by a red box labelled as “not that important”, scores 4–6 as an amber box labelled as “important” and scores 7–9 as a green box labelled “really important”… Participants [health care professionals] were shown their own score from [a previous round] alongside the percentage of participants giving each score [1 to 9] from their own stakeholder group.”^*^ [23]

*^*^In the above example, the health care professionals were also shown the results to all stakeholder groups (including patient and parents) in a further round [See Figure 1].*

**Example:** “Participants were asked to divide 100 points among the domains they considered important. In Delphi round 2 a list of domains, with the average score assigned in the first round, were distributed. Domains with high agreement (more than an average of 10 points) were included; domains with low agreement (less than an average score of 3 points) were excluded. Invitees were again asked to divide 100 points among the remaining domains that were considered very important.” [58]

**Item 9a: CONSENSUS DEFINITION:** Describe the consensus definition

**Explanation:** The choice of what consensus criteria to use is an important consideration in COS development. Criteria that are too accommodating may result in a long list of outcomes that are not considered to be minimal, whilst too stringent criteria may exclude key outcomes that may otherwise have been included in the COS. There are numerous ways proposed to define the consensus criteria, although the choice of criteria is rarely justified in the published literature [60]; commonly these relate to a threshold of percentage agreement or a mean or median threshold value for each outcome [22, 45-46, 55-56]. Regardless of the consensus criteria that are used, authors should clearly describe the consensus definition and justify this choice where possible. As part of an ‘issues to consider’ paper, Williamson et al 2012 describe the rationale for a ‘70/15%’ consensus definition which was used in the second example provided below [1]. As we described in Item 4, it is important for authors to define the consensus criteria a priori (and ideally to publish it in a protocol) to minimise any potential bias from changing the criteria after the results have been analysed [41].

**Example:** “Consensus that an outcome is important for eczema was defined as a score of 7 or more (as described above, a score of 7–9 indicated that the participating experts felt that a certain domain was important to include) by at least 60% of all members of at least three stakeholder groups [from four in total] including consumers.” [56]

**Example: Table 2 | Example of definition of consensus**

(Adapted from Table 2 of [42])

| Consensus classification | Description | Definition |
| --- | --- | --- |
| Consensus in | Consensus that outcome  should be included in  the core outcome set | 70% or more participants  scoring as 7 to 9 AND  <15% participants scoring  as 1 to 3 |
| Consensus out | Consensus that outcome  should not be included in  the core outcomes set | 70% or more participants  scoring as 1 to 3 AND <15%  of participants scoring  as 7 to 9 |
| No consensus | Uncertainty about  importance of outcome | Anything else |

**Example:** “‘Consensus in’ (consensus that the outcome should be included in core set) will be defined as greater than 70% of participants scoring between seven and nine [1-9 scale was used for scoring] and less than 15% of participants scoring between one and three. ‘Consensus out’ (consensus that the outcome should not be included in core set) will be defined as greater than 70% of participants scoring between one to three and less than 15% of participants scoring between seven and nine. All other combinations will be considered ‘equivocal’. The outcomes that are designated as ‘consensus in’ by both stakeholder groups will be included in the final core outcome set to be carried forward to the consensus meeting.” [28]

**Item 9b: CONSENSUS DEFINITION:** Describe the procedure for determining how outcomes were included or excluded from consideration during the consensus process

**Explanation:** Different approaches have been used to include or exclude outcomes from consideration during the consensus process, but there is currently no empirical evidence to suggest whether the different approaches impacts on the final COS. We therefore strongly recommend that authors describe any pre-specified criteria for excluding outcomes during the consensus process, and provide the reasons for these criteria. Authors should also state where outcomes are carried forward throughout the whole consensus process. Additional outcomes may also be introduced during the consensus process if new outcomes are suggested which were not identified during the initial outcome generation process (see Item 6a). Authors should describe the process for introducing new outcomes and how these new outcomes are to be considered throughout the remainder of the process.

**Example:** “In round 1 of the survey, items were categorized as ‘essential’ and retained for round 2 if they were rated between 7 and 9 by over 50 per cent of respondents *and* between 1 and 3 by less than 15 per cent. Items not meeting these criteria were discarded…Round 2 responses were analysed with stricter cut-off criteria, retaining items rated between 7 and 9 by over 70 per cent of respondents, and between 1 and 3 by less than 15 per cent… Items retained after round 2 were considered in phase 3 consensus meetings…All items retained from both meetings were included in the final core set.” [45]

**Example:** “Pain features were retained and carried forward [to the next round] if they were given an importance rating of 7–9 by ≥70% of both panels and 1–3 by ≤15% of both panels, or rated as 7–9 by ≥90% of one panel.” [22]

**Example:** “Likert items rated as “Very important” or “Extremely important” by at least 80% of panellists were automatically included in the list of Delphi recommendations. Conversely, items rated “Of no importance” or “Of little importance” by at least 50% of panellists, or rated as “Important”, “Very important”, or “Extremely important” by less than 75% of panellists were automatically excluded from the next survey round. Items rated “Important”, “Very important” or “Extremely important” by at least 75% of panellists were forwarded to the subsequent round for re-rating… Parameter items that received at least 50% of panellists’ votes were automatically included in the Delphi recommendations; remaining items were forwarded to the subsequent round for re-rating.” [20]

**Example:** Study Background: two-round Delphi survey

“Participants were invited to recommend additional potential outcomes for consideration at the end of the survey [Round 1] using free-text responses….Participants were asked to score *all* the individual outcomes again using the same 9-point Likert scale [Round 2]… No outcomes were excluded in this round to ensure a holistic approach to scoring in round 2”. [34]

**Item 10: ETHICS AND CONSENT:** Provide a statement regarding the ethics and consent issues for the study

**Explanation:** Consideration of ethical and consent issues is important for any medical research and an ethics statement is often a requirement for journal submissions. The level of regulatory requirements for a COS study is variable and may depend on the country where the research is undertaken or the methods and stakeholder groups that were involved in the consensus process.

Authors should describe ethical and consent issues associated with the COS project and describe how these issues were handled. For example, in a Delphi survey, where participation is optional, authors may include an informed consent statement in the initial survey invitation suggesting that informed consent is assumed if a participant responds to the survey. Authors should reference any ethical approvals obtained and should declare if ethical approval was not needed.

**Example:** “Ethical approval was received from the National Research Ethics Service North West – Greater Manchester East Research Ethics Committee (Reference 11/NW/0586) for the completion of semi-structured interviews and invitation of interviewees to the final consensus meeting. Written consent was sought for participation in semi-structured interviews, with written proxy consent sought from parents/guardians for their child’s participation. Written assent was also sought from children aged 6 years and older. Attendance at the consensus meeting was considered to be implied consent for participation with no written consent provided, this process was approved by the Research Ethics Committee. Advice was sought from the National Research Ethics Service who did not consider that ethical approval was required for an online survey of parental and child opinion. However, full information about the study was given in the initial pages of the online survey and survey completion considered to imply consent.” [23]

**Example:** “The Ethics Committee [in Warsaw, Poland], was contacted; no special permission was deemed to be required.” [38]

**Example:** “Following site-specific ethics approval for this study at each major paediatric rheumatology centre already participating in the Juvenile Dermatomyositis Cohort and Biomarker Study (UK and Ireland), parents and patients will be approached by their local doctors/nurses and asked whether they would be interested in taking part in this study… Informed consent will be taken from each participant. Collaborators in Italy and the Netherlands will attempt to replicate this process (with country-specific ethics approval and translation as needed).” [36]

**RESULTS**

**Item 11: PROTOCOL DEVIATIONS:** Describe any changes from the protocol (if applicable), with reasons, and a describe what impact these changes have on the results

**Explanation:** For transparency purposes, it is recommended that authors should report all departures from their protocol, including unplanned changes to the involvement of different stakeholder groups, methods and analysis of the consensus process and the consensus definition. The nature of the protocol deviation and the exact reason for the change should always be reported alongside any known impact that these changes may have on the results.

**Example:** Study Background: The stakeholder groups listed in the protocol were *“*clinician stakeholder group…are as follows: audiologist…, cleft surgeon…, ear nose and throat surgeon…, paediatrician…, specialist cleft nurse…, speech and language therapist…and psychologist…”

“[There was only one protocol deviation in this study]….of the three paediatricians who took part, all had a speciality in audiology, consequently for future rounds their scores were combined with audiologists into a new group ‘audiologists and audiological physicians’ as agreed by the SAG [scientific advisory group]. [It was thought that this change would not impact on the final result to the COS as the opinions of the two combined stakeholder groups were likely to be similar.]” ([23] adapted).

**Item 12: PARTICIPANTS:** Present data on the number and relevant characteristics of the people involved at all stages of COS development

**Explanation:** It is important to know the number and characteristics of the individual stakeholder group members who actually participated. This information allows the readers, in particular COS users, to judge the value of the COS based on considerations of generalizability and the numbers and relative proportion of the different stakeholders groups participating.

**Example:** Study Background: to develop a core outcome set for research and audit studies in reconstructive breast surgery including patients, breast and plastic surgeons, specialist nurses and psychologists

*“*Demographics of patient and professional participants...(Table 2)*”*see Table 3 [46]

**Table 3** | Demographics of patient and professional participants in both rounds of a Delphi and consensus meeting

(From Table 2 of [46])

|  | Round 1  n(%) | Round 2  n(%) | Consensus Meeting  n(%) |
| --- | --- | --- | --- |
|  |  |  |  |
| **Patient participants** | ***n* = 215** | ***n* = 190** | ***n* = 15** |
| Centre |  |  |  |
| Bristol | 77 (35·8) | 68 (35·8) | 13 (87) |
| Liverpool | 74 (34·4) | 62 (32·6) | 2 (13) |
| Glasgow | 64 (29·8) | 60 (31·6) | 0 (0) |
| Age (years) |  |  |  |
| < 45 | 21 (9·8) | 20 (10·5) | 2 (13) |
| 45–65 | 166 (77·2) | 146 (76·8) | 9 (60) |
| > 65 | 28 (13·0) | 24 (12·6) | 4 (27) |
| Median (range) | 54 (29–76) | 54 (29–76) | 55 (43–76) |
| Time since breast reconstruction (months) |  |  |  |
| 0–24 | 72 (33·5) | 67 (35·3) | 4 (27) |
| 25–48 | 88 (40·9) | 75 (39·5) | 10 (67) |
| > 48 | 49 (22·8) | 42 (22·1) | 1 (7) |
| Unknown | 6 (2·8) | 6 (3·2) | 0 (0) |
| Median (range) | 33 (4–97) | 32 (4–97) | 35 (21–72) |
| Timing of surgery |  |  |  |
| Immediate reconstruction | 110 (51·2) | 100 (52·6) | 10 (67) |
| Delayed reconstruction | 80 (37·2) | 66 (34·7) | 4 (27) |
| Therapeutic mammoplasty | 25 (11·6) | 24 (12·6) | 1 (7) |
| Type of surgery |  |  |  |
| Immediate reconstruction | 110 (51·2) | 100 (52·6) | 10 (67) |
| Delayed reconstruction | 80 (37·2) | 66 (34·7) | 4 (27) |
| Therapeutic mammoplasty | 25 (11·6) | 24 (12·6) | 1 (7) |
| Type of surgery |  |  |  |
| Implant-based reconstruction | 54 (25·1) | 47 (24·7) | 4 (27) |
| Latissimus dorsi flap | 59 (27·4) | 52 (27·4) | 5 (33) |
| Abdominal flap | 74 (34·4) | 64 (33·7) | 5 (33) |
| Therapeutic mammoplasty | 25 (11·6) | 24 (12·6) | 1 (7) |
| Other | 3 (1·4) | 3 (1·6) | 0 (0) |
| Education |  |  |  |
| Compulsory only | 65 (30·2) | 57 (30·0) | 3 (20) |
| Additional education | 139 (64·7) | 125 (65·8) | 11 (73) |
| Unknown | 11 (5·1) | 8 (4·2) | 1 (7) |
| Marital status |  |  |  |
| Single | 23 (10·7) | 18 (9·5) | 0 (0) |
| Married/living with partner | 153 (71·2) | 138 (72·6) | 13 (87) |
| Separated or divorced | 28 (13·0) | 26 (13·7) | 2 (13) |
| Widowed | 6 (2·8) | 4 (2·1) | 0 (0) |
| Unknown | 5 (2·3) | 4 (2·1) | 0 (0) |
| Employment status |  |  |  |
| Full- or part-time employment | 130 (60·5) | 118 (62·1) | 11 (73) |
| Homemaker/housewife | 17 (7·9) | 11 (5·8) | 0 (0) |
| Retired | 45 (20·9) | 42 (22·1) | 3 (20) |
| Not working | 16 (7·4) | 13 (6·8) | 0 (0) |
| Unknown | 7 (3·3) | 6 (3·2) | 1 (7) |
| **Professional participants** | ***n* = 88** | ***n* = 69** | ***n* = 23** |
| Sex |  |  |  |
| F | 46 (52) | 37 (53·6) | 14 (61) |
| M | 42 (48) | 32 (46·4) | 9 (39) |
| Profession |  |  |  |
| Consultant breast surgeon | 40 (45) | 35 (51) | 11 (48) |
| Consultant plastic surgeon | 21 (24) | 15 (22) | 5 (22) |
| Clinical nurse specialist | 20 (23) | 15 (22) | 6 (26) |
| Psychologist | 7 (8) | 4 (6) | 1 (4) |
| Time in post (years) |  |  |  |
| < 5 | 18 (20) | 12 (17) | 4 (17) |
| 5–10 | 30 (34) | 24 (35) | 9 (39) |
| 10–20 | 29 (33) | 25 (36) | 9 (39) |
| > 20 | 8 (9) | 6 (9) | 0 (0) |
| Unknown | 3 (3) | 2 (3) | 1 (4) |

**Item 13a: OUTCOMES:** List all outcomes considered at the start of the consensus process

**Explanation:** It is important for authors to list all the outcomes that were considered at the start of the consensus process to allow COS users to identify whether any important outcomes were missing. Listing the outcomes can confirm whether the item was considered but was subsequently not included in the final COS because it did not reach the consensus criteria. As the list of outcomes may be long, authors may consider including the full list of outcomes considered at the start of the consensus process in a supplementary document to their main report.

**Example:** Study Background: to develop a core outcome set for otitis media with effusion in children with cleft palate

**Table 4** | Domains, outcomes and outcome tips used in the health professional Delphi

(Adapted from Table 19 of [61])

| **Domain** | **Outcome** | **Tip [further explanation]** |
| --- | --- | --- |
| Outcomes related to  Behaviour | Externalising behaviour | Externalising behaviours are directed outwards (e.g. having a tantrum) |
|  | Internalising behaviour | Internalising behaviours are directed inwards (e.g. being withdrawn or lonely) |
| Outcomes related to COM [chronic otitis media] | Atelectasis | Retraction of the thin tympanic membrane with loss of the normal middle ear space |
|  | Cholesteatoma | Structure made of keratin not usually found in the middle ear. Has tendency to enlarge and cause recurrent ear discharge and hearing loss |
|  | COM | Fluid in the middle ear persisting for over 3 months |
|  | Persistent tympanic membrane  Perforation | Hole in the tympanic membrane |
|  | Persistent tympanic membrane retraction | Tympanic membrane pulled backwards due to negative pressure in the middle ear |
|  | Tympanosclerosis | Damage to the tympanic membrane with resultant deposition of calcium within tympanic membrane |

**Cont…**

**Item 13b: OUTCOMES:** Describe any new outcomes introduced and any outcomes dropped, with reasons, during the consensus process

**Explanation:** The procedure for determining how outcomes were included or excluded from consideration during the consensus process was addressed in Item 9b. Authors should detail with reason which outcomes were introduced and dropped and at what stage during the consensus process, to enable the reader to assess whether the decisions were made in an unbiased way. It is also important that newly introduced outcomes are mentioned as these will not be amongst those listed at the start of the consensus process (Item 13a). Details of who suggested the additional outcomes (e.g. a patient or health care professional) would also be beneficial as this denotes the importance of the outcome to a particular stakeholder group.

**Example:** Study Background: three round Delphi of healthcare professionals

**“**In round 2 a total of 47 outcomes, representing the original list of 45 plus the addition of two outcomes [listening skills, psychosocial wellbeing], identified by health professionals’ free-text responses in round 1, were scored by participants" [See Figure 1] [61]

**Example:** Study Background: two-round Delphi survey followed by a consensus meeting.  During the Delphi, items were disregarded if they did not meet certain criteria (see example 1, item 9b)

*“*In round 1, none of the items met the exclusion criteria, so all 34 items were carried forward to round 2…. Provision of feedback, advice regarding reprioritization of the most important items and application of the more stringent cut-off criteria resulted in the exclusion of 15 items following round 2 (*Fig. 1*). Nineteen items were carried forward for discussion in the phase 3 consensus meetings.*”*

[Fig. 1 in the paper identifies that the 15 excluded items were:  Systemic complications, Long-term wound complications, Breast symptoms, Arm and shoulder symptoms, Implant symptoms, Objective cosmetic outcome, No. of procedures, Fatigue, Recovery time, Duration of procedure, Time to complete reconstruction, Clothing issues, Financial issues, Economic issues, Cosmetic outcome assessed by patient’s partner] [45]

**Item 14: CORE OUTCOME SET:** List the outcomes in the final core outcome set

**Explanation:** The ultimate objective of a COS study is to develop a minimum set of outcomes. There are examples in the literature where the set of recommended outcomes from a COS study are unclear or ambiguous, which may deter or limit researchers from using the COS. Authors of COS studies should clearly list the core outcomes that are recommended, either as a single sentence statement or preferably under a separate section of “Recommended set of core outcomes” for added clarity. The authors should also describe any important detail about any of the core outcomes that a user of the core outcomes would need to know. In the first example below, the requirement to measure the core outcome ‘radiographs of the joint’, is only relevant to studies where the duration of follow-up is more than one year.

**Example: “**This core set includes the following measures: pain, patient global assessment, physical disability, swollen joints, tender joints, acute phase reactants, and physician global assessment; in studies of one or more years’ duration, radiographs of joints should be performed.” [62]

**Example:** “Respondents agreed that symptoms of eczema, clinical signs assessed by a physician, and a measurement for long term control of flares should be included in the core set of outcomes for eczema trials.” [56]

**DISCUSSION**

**Item 15: LIMITATIONS:** Discuss any limitations in the COS development process

**Explanation:** A discussion of the limitations should address issues relevant to the validity and generalizability (applicability) of the COS. Readers may find it useful if authors discuss whether any changes from the original protocol (see item 11) may have impacted on the final COS.

Limitations of the consensus process might include issues with the representativeness from stakeholder groups, e.g. missing stakeholder groups from the COS developmental process, low numbers of participants in particular groups, or high attrition rates (between rounds of a Delphi survey). Applicability of the COS may be affected by the scope of the COS (as defined in Item 3) or representativeness in terms of geographic coverage, if for example, all participants in the COS decision making were from a single country or region.

Authors should also use this section to inform the reader about any other limitations in the COS development process that they feel are important.

**Example:** “Although individuals from all areas of the world were invited, colleagues from Africa, China or India were not able to attend this particular meeting and only one US invitee was able to attend. Also, a more equal representation of some stakeholders groups, such as the regulators, pharmaceutical industry and patients with a range of disease severity or in remission, could enhance the representativeness of our findings and recommendations.” [56]

**Example:** “Low response rate is another potential limitation of our study. Among clinicians, very few initially answered the invitation to phase 1. Those who finally did were asked more directly. This potentially could have resulted in bias.” [38]

**Example:** “The main limitation of the study is that it was conducted solely within the UK, which has a state-funded healthcare system. It is unclear to what degree the outcomes valued in this setting would be concordant with those valued in other healthcare systems or cultural settings. This may limit the generalizability of the results.” [46]

**Item 16: CONCLUSIONS:** Provide an interpretation of the final COS in the context of other evidence, and implications for future research

**Explanation:** Authors should try to relate the final COS to other evidence, for example if other COS exist in the same area of health then authors should help readers better understand the similarities and differences between them. For example, authors should discuss any differences in methodology, scope of the COS (Item 3) or stakeholder involvement. We also advise authors to make explicit recommendations regarding future work. The COS-STAR reporting guideline refers to the reporting of COS studies that look to identify what outcomes should be measured, but authors should discuss the need to identify how the outcomes in the COS should be measured.

**Example: “**A consensus-based COS for NSLBP [non-specific lower back pain] was developed and included the domains ‘physical functioning’, ‘pain intensity’, ‘health-related quality of life’ and ‘number of deaths’. This COS represents the update of the standardized set proposed by [another study group]. The brevity of this COS should facilitate its implementation in clinical trials assessing efficacy or effectiveness of health interventions for NSLBP. Future research should establish measurement instruments are the most appropriate to measure these core outcome domains.” [49]

**Example:** “In conclusion, this study has highlighted the importance of a comprehensive and multidimensional approach to the assessment of CPSP [chronic post-surgical pain] after TKR [total knee replacement]. This is reflected in the 8-item core outcome set, which recommends that pain assessment should include the following pain domains: pain intensity, pain interference with daily living, pain and physical functioning, temporal aspects of pain (time and pain), pain description, emotional aspects of pain, use of pain medication, and improvement and satisfaction with pain relief. This condition-specific core outcome set reflects IMMPACT [Initiative on Methods, Measurement, and Pain Assessment in Clinical Trials] assessment recommendations for clinical trials investigating treatments for chronic pain. The findings from this study have the potential to facilitate a move towards improving the quality and consistency of pain assessment within orthopaedic surgery.” [22]

**OTHER INFORMATION**

**Item 17: FUNDING:** Describe sources of funding, role of funders

**Explanation:** Authors of COS studies, like those of any other research study, should disclose any funding they received to develop the COS, or state if no funding was received. Given the potential role of funding bodies as key stakeholders in the COS development process (e.g. authorities and industry representatives) [14], it is important to be transparent about funding and the role of funders, if any. A funder may provide a service, such as the venue for a consensus meeting, with representatives of the funding body involved in the consensus meeting discussions. Any level of funding or service provided to the COS development team should be reported. Authors should also declare whether the funder had a role in the conduct or the report of the COS study.

**Example:** “This article presents independent research funded by the National Institute for Health Research (NIHR) under its Programme Development Grants scheme (Reference Number: RP-DG-1211-10002). The views expressed are those of the authors and not necessarily those of the NHS, the NIHR or the Department of Health. The research team acknowledge the support of the NIHR, through the Comprehensive Clinical Research Network.” [22]

**Example:** *A description of how funder was involved in the consensus process*

“…the NCI’s [National Cancer Institute] Symptom Management and Health-Related Quality of Life Steering Committee sponsored a CTPM [Clinical Trials Planning Meeting]… The meeting included interdisciplinary investigators in cancer outcomes research and clinical trials, representing expertise in developmental therapeutics, cancer symptom and HRQOL [health related quality of life] assessment, measurement methodology, and statistics, as well as representatives from the patient advocacy community, clinical trial cooperative group administration, the pharmaceutical industry, the NCI, and the US Food and Drug Administration… The recommended core symptom set for adult cancer treatment trials was endorsed by NCI’s Symptom Management and Health-Related Quality of Life Steering Committee, Clinical and Translational Research Operations Committee, and Clinical Trials and Translational Research Advisory Committee.” [9]

**Item 18: CONFLICTS OF INTEREST:** Describe any conflicts of interest within the study team and how these were managed

**Explanation:** In addition to funding issues (Item 17), authors should report any perceived conflicts of interest related to their role (or that of other members of the study team) within the COS development process which might be seen as influential. Sometimes there might be situations where an author has developed a measurement instrument for one of the outcomes being discussed for potential inclusion into the COS. Authors should declare if there are no known conflicts of interest.

**Example:** “Although attempts were made to control for conflicts of interest (such as developers of measures in contention in round three [of the Delphi survey] not participating in the final vote), there is no guarantee that they were eliminated...Individuals [attending the panel meeting] were not excluded if they were the developer of one of the measures under consideration, but all panel members declared at the meeting whether they had any conflicts of interest related to participating in balance COS recommendations (including authorship) of measures under consideration for the balance COS.” [63]

**Example:** “The authors have no conflicts of interest to report.” [64]

**Endorsement and Implementation**

If the COS-STAR Statement is endorsed by journals and adhered to by COS developers, there should be evidence of improved reporting of COS development studies. A strength of this study is that we have involved the combined experience of COS experts from both the COMET management group (DA, JB, MC, ST, PRW) and the executives of The EQUATOR (Enhancing the QUAlity and Transparency Of health Research) Network (DGA, DM) in the development of this guideline. The overall aim of EQUATOR is to improve the quality of reporting of all health science research through the development and translation of reporting guidelines.

The post-publication activities recommended by EQUATOR include seeking and responding to criticism, encouraging the endorsement of and adherence to the guideline from various stakeholders, translating the guideline into other languages, evaluating its impact, ensuring website development, and updating of the guideline (see Discussion section). The COS-STAR checklist and related publications are freely available on the websites of the COMET website (<http://www.comet-initiative.org/>) and EQUATOR network (www.equator-network.org). The COMET website and social media [@COMETinitiative](https://twitter.com/COMETinitiative), [www.twitter.com/COMETinitiative](http://www.twitter.com/COMETinitiative)) will be used to make announcements about the launch of COS-STAR, educational initiatives and the COMET website will serve as a portal to receive comments and feedback via the general enquiry link (http://www.cometinitiative.org/contactus). We will encourage journals publishing COS development studies to modify their ‘Instructions for Authors’ section to endorse COS-STAR and to consider publishing COS protocols, if they do not do so already. Following sufficient time for wide dissemination of COS-STAR, we aim to evaluate the endorsement and adherence to the COS-STAR Statement using similar methods to those used to evaluate other guidelines [65-67].

To overcome some of the challenges with reporting guideline uptake we will develop an implementation strategy similar to that developed for PRISMA-P (Preferred reporting items for systematic review and meta-analysis protocols) [68]. For instance, we will engage with relevant stakeholders (including those involved in our Delphi exercise and consensus meeting) to assist in the implementation of COS-STAR. Any planned implementation will help improve the quality, completeness and consistency of the reporting of COS studies.

**Discussion**

The COS-STAR checklist was developed using an approach that has been recommended for developing medical reporting guidelines [69]. The overall aim of the COS-STAR Statement is to improve the clarity and transparency of reporting of COS development studies.

This COS-STAR Explanation and Elaboration document was developed to help facilitate the understanding and uptake of the COS-STAR Statement and provide examples of good reporting practice using a structured framework for those interested in conducting and reporting COS development studies. It follows a format similar to other explanatory documents for reporting guidelines [6]. The COS-STAR Statement does not make recommendations about which methodology should be used or which stakeholder groups should be included in reaching consensus in COS development projects. COS-STAR is not a quality assessment tool and should not be used in this way, but the reporting items may facilitate discussion around some of the design considerations when conducting a COS study. Importantly, we believe that use of the COS-STAR Statement by authors will help COS users (e.g. trialists, systematic reviewers and policy makers) critically appraise the COS, and help them decide whether the COS is useful for their own purpose.

The COMET (Core Outcome Measures in Effectiveness Trials) Initiative was launched in 2010 (<http://www.comet-initiative.org/about/overview>) and while COS development pre-dates this, the literature on COS and the publication of COS development studies has grown rapidly since this launch. Nevertheless, a limitation is that there are a relatively small number of studies to draw examples of rationale and good reporting practice. Our familiarisation with the COS development studies identified in a recent review [10], goes some way in ensuring that our exemplar case selections were evidence-based wherever possible. For some checklist items such as reporting the abstract (Item 1b), we have adapted a published example to improve the reporting standard further. We hope that the COS-STAR Statement will act as a catalyst to help generate further examples and evidence that can be used in future revisions of the checklist.

Table 1: Core Outcome Set-STandards for Reporting: The COS-STAR Statement

| **SECTION/TOPIC** | **ITEM No.** | **CHECKLIST ITEM** |
| --- | --- | --- |
| **TITLE/ABSTRACT** |  |  |
| Title  Abstract | 1a | Identify in the title that the paper reports the development of a COS |
|  | 1b | Provide a structured summary |
| **INTRODUCTION** |  |  |
| Background and objectives | 2a | Describe the background and explain the rationale for developing the COS |
|  | 2b | Describe the specific objectives with reference to developing a COS |
| Scope | 3a | Describe the health condition(s) and population(s) covered by the COS |
|  | 3b | Describe the intervention(s) covered by the COS |
|  | 3c | Describe the setting(s) in which the COS is to be applied |
| **METHODS** |  |  |
| Protocol/Registry Entry | 4 | Indicate where the COS development protocol can be accessed, if available and/or the study registration details |
| Participants | 5 | Describe the rationale for stakeholder groups involved in the COS development process, eligibility criteria for participants from each group and a description of how the individuals involved were identified |
| Information sources | 6a | Describe the information sources used to identify an initial list of outcomes |
|  | 6b | Describe how outcomes were dropped/combined, with reasons (if applicable) |
| Consensus process | 7 | Describe how the consensus process was undertaken |
| Outcome scoring | 8 | Describe how outcomes were scored and scores summarised |
| Consensus definition | 9a | Describe the consensus definition |
|  | 9b | Describe the procedure for determining how outcomes were included or excluded from consideration during the consensus process |
| Ethics and consent | 10 | Provide a statement regarding the ethics and consent issues for the study |
| **RESULTS** |  |  |
| Protocol deviations | 11 | Describe any changes from the protocol (if applicable), with reasons, and a describe what impact these changes have on the results |
| Participants | 12 | Present data on the number and relevant characteristics of the people involved at all stages of COS development |
| Outcomes | 13a | List all outcomes considered at the start of the consensus process |
|  | 13b | Describe any new outcomes introduced and any outcomes dropped, with reasons, during the consensus process |
| Core outcome set | 14 | List the outcomes in the final core outcome set |
| **DISCUSSION** |  |  |
| Limitations | 15 | Discuss any limitations in the COS development process |
| Conclusions | 16 | Provide an interpretation of the final COS in the context of other evidence, and implications for future research |
| **OTHER INFORMATION** |  |  |
| Funding | 17 | Describe sources of funding, role of funders |
| Conflicts of interest | 18 | Describe any conflicts of interest within the study team and how these were managed |

**Funding**

Financial support for the COS-STAR consensus meeting was provided by the MRC Network of Hubs for Trials Methodology Research (MR/L004933/1-R55).

**Author Contributions**

ICMJE criteria for authorship read and met: JJK, SG, DGA, JB, MC, DD, EG, DM, JS, PT, ST, PRW. Conceived the idea for the study: PRW. Wrote the first draft of the paper: JJK, and identified initial examples: JJK, SG, PRW. All authors read and approved the final manuscript.

**Conflicts of Interest**

DGA, JMB, MC, EG, PRW and ST are members of the COMET Management Group. DM is a member of the Editorial Board of PLOS Medicine. DD, SG, JJK, JS and PT declare no competing interests.

**Ethical approval**

The University of Liverpool Ethics Committee was consulted and granted ethical approval for this study (Reference RETH000841).

**Acknowledgments**

The study team would like to thank all those members who contributed to the consensus meeting and provided some of the rationale for including the items into the COS-STAR checklist:

Sara Brookes, School of Social and Community Medicine, University of Bristol (Bristol, UK); Sally Crowe, Crowe Associates (Oxen, UK); Mandy Daly, Irish Neonatal Health Alliance (Wicklow, Ireland); Christopher Eccleston, Department for Health, University of Bath (Bath, UK); Trish Groves, The BMJ, (London, UK); Kirstie Haywood, Royal College of Nursing Research Institute, University of Warwick (Warwick, UK); Angelos Kolias, Cambridge Neuroscience, University of Cambridge (Cambridge, UK); Monika Nothacker, Guidelines International Network (Berlin, Germany); Larry Peiperl, PLOS Medicine (San Francisco, USA); Daniel Shanahan, BioMed Central (London, UK); Toni Tan, The National Institute for Health and Care Excellence (Manchester, UK); Sean Tunis, Center for Medical Technology Policy (Baltimore, USA); Jilda Vargus-Adams, [Cincinnati Children's Hospital Medical Center](http://www.cincinnatichildrens.org/) (Cincinnati, USA); Jos Verbeek, Finnish Institute of Occupational Health (Helsinki, Finland).

**References**

[1] Williamson PR, Altman DG, Blazeby JM, Clarke M, Devane D et al. Developing core outcome sets for clinical trials: issues to consider. *Trials* 2012; 13:132. **doi:** 10.1186/1745-6215-13-132

[2] Kirkham JJ, Gorst S, Altman D, Blazeby J, Clarke M et al. Core Outcome Set-STAndards for Reporting: The COS-STAR Statement. *Submitted to PLoS Medicine*

[3] Williamson PR, Blazeby J, Clarke M, Bagley H, McNair A et al. The COMET Handbook. *Submitted to Trials*

[4] Schmitt J, Apfelbacher C, Spuls PI, Thomas KS, Simpson E et al. The Harmonizing Outcome Measures for Eczema (HOME) roadmap: A methodological framework to develop core sets of outcome measurements in dermatology. Journal of Investigative Dermatology. 2015; 135 (1):24-30. [doi:10.1038/jid.2014.320](http://dx.doi.org/10.1038/jid.2014.320)

[5] Boers M, Kirwan JR, Wells G, Beaton D, Gossee L et al. Developing core outcome measurement sets from clinical trials: OMERACT filter 2.0. *Journal of Clinical Epidemiology* 2014; 67:745-53. doi: http://dx.doi.org/10.1016/j.jclinepi.2013.11.013

[6] Liberati A, Altman DG, Tetzlaff J, Mulrow C, Gøtzsche PC, Ioannidis JPA, et al. The PRISMA Statement for Reporting Systematic Reviews and Meta-Analyses of Studies That Evaluate Health Care Interventions: Explanation and Elaboration. *PLoS Medicine* 2009; 6(7): e1000100. doi:10.1371/journal.pmed.1000100

[7] Taylor WJ. Preliminary identification of core domains for outcome studies in psoriatic arthritis using Delphi methods. *Ann Rheum Dis* 2005; 64(Suppl II):ii110–ii112 doi:  [10.1136/ard.2004.030874](http://dx.doi.org/10.1136%2Fard.2004.030874)

[8] Zannad F, Garcia AA, Anker SD, Armstrong PW, Calvo G et al. Clinical outcome endpoints in heart failure trials. A European society of cardiology heart failure association consensus document. *Eur J Heart Fail* 2013; 15(10):1082-94. doi: [10.1093/eurjhf/hft095](http://dx.doi.org/10.1093/eurjhf/hft095)

[9] Reeve BB, Mitchell SA, Dueck AC, Basch E, Cella D et al. Recommended patient-reported core set of symptoms to measure in adult cancer treatment trials. [J Natl Cancer Inst.](http://www.ncbi.nlm.nih.gov/pubmed/25006191) 2014; 8: 106(7): dju129. doi:10.1093/jnci/dju129

[10] Gorst SL, Gargon E, Clarke M, Blazeby JM, Altman DG et al. Choosing Important Health Outcomes for Comparative Effectiveness Research: An Updated Review and User Survey. *PLoS ONE* 2016; 11(1): e0146444. doi:10.1371/journal.pone.0146444

[11] Bastian H, Glasziou P, Chalmers I. Seventy-five trials and eleven systematic reviews a day: how will we ever keep up? *PloS Medicine* 2010; 7(9): e1000326. doi: 10.1371/journal.pmed.1000326

[12] Schulz KF, Altman DG, Moher D, for the CONSORT Group. CONSORT 2010 Statement: updated guidelines for reporting parallel group randomised trials. *PLoS Medicine* 2010; 7(3): e1000251. doi: 10.1371/journal.pmed.1000251

[13] Moher D, Liberati A, Tetzlaff J, Altman DG, The PRISMA Group. Preferred Reporting Items for Systematic Reviews and Meta-Analyses: The PRISMA Statement. *PLoS Medicine* 2009; 6(7): e1000097. doi:10.1371/journal.pmed.1000097

[14] Gargon E, Gurung B, Medley N, Altman DG, Blazeby JM et al. Choosing Important Health Outcomes for Comparative Effectiveness Research: A Systematic Review. *PLoS ONE* 2014; 9(6): e99111. doi:10.1371/journal.pone.0099111

[15] Gargon E, Williamson PR and Clarke M. Collating the knowledge base for core outcome set development: developing and appraising the search strategy for a systematic review. *BMC Medical Research Methodology* 2015; 15:26. **doi:** 10.1186/s12874-015-0019-9

[16] Cook RJ, Heddle NM. Clinical trials evaluating pathogen-reduced platelet products: methodologic issues and recommendations. *Transfusion* 2013; 53(8): 1843-55. doi: 10.1111/j.1537-2995.2012.03951.x

[17] [Eliasson AC](http://www.ncbi.nlm.nih.gov/pubmed/?term=Eliasson%20AC%5BAuthor%5D&cauthor=true&cauthor_uid=24266735), [Krumlinde-Sundholm L](http://www.ncbi.nlm.nih.gov/pubmed/?term=Krumlinde-Sundholm%20L%5BAuthor%5D&cauthor=true&cauthor_uid=24266735), [Gordon AM](http://www.ncbi.nlm.nih.gov/pubmed/?term=Gordon%20AM%5BAuthor%5D&cauthor=true&cauthor_uid=24266735), [Feys H](http://www.ncbi.nlm.nih.gov/pubmed/?term=Feys%20H%5BAuthor%5D&cauthor=true&cauthor_uid=24266735), [Klingels K](http://www.ncbi.nlm.nih.gov/pubmed/?term=Klingels%20K%5BAuthor%5D&cauthor=true&cauthor_uid=24266735) et al. Guidelines for future research in constraint-induced movement therapy for children with unilateral cerebral palsy: an expert consensus. *Developmental Medicine & Child Neurology* 2014; 56(2): 125-37.

[18] [Smith CA](http://www.ncbi.nlm.nih.gov/pubmed/?term=Smith%20CA%5BAuthor%5D&cauthor=true&cauthor_uid=24559820), [Betts D](http://www.ncbi.nlm.nih.gov/pubmed/?term=Betts%20D%5BAuthor%5D&cauthor=true&cauthor_uid=24559820). The practice of acupuncture and moxibustion to promote cephalic version for women with a breech presentation: implications for clinical practice and research. *Complementary Therapies in Medicine* 2014; 22(1): 75-80. doi: 10.1016/j.ctim.2013.12.005

[19] [Spragg RG](http://www.ncbi.nlm.nih.gov/pubmed/?term=Spragg%20RG%5BAuthor%5D&cauthor=true&cauthor_uid=20224063), [Bernard GR](http://www.ncbi.nlm.nih.gov/pubmed/?term=Bernard%20GR%5BAuthor%5D&cauthor=true&cauthor_uid=20224063), [Checkley W](http://www.ncbi.nlm.nih.gov/pubmed/?term=Checkley%20W%5BAuthor%5D&cauthor=true&cauthor_uid=20224063), [Curtis JR](http://www.ncbi.nlm.nih.gov/pubmed/?term=Curtis%20JR%5BAuthor%5D&cauthor=true&cauthor_uid=20224063), [Gajic O](http://www.ncbi.nlm.nih.gov/pubmed/?term=Gajic%20O%5BAuthor%5D&cauthor=true&cauthor_uid=20224063) et al. Beyond mortality - future clinical research in acute lung injury. *Am J Respir Crit Care Med* 2010; 181(10):1121-7. doi: 10.1164/rccm.201001-0024WS

[20] [Ward L](http://www.ncbi.nlm.nih.gov/pubmed/?term=Ward%20L%5BAuthor%5D&cauthor=true&cauthor_uid=24942270), [Stebbings S](http://www.ncbi.nlm.nih.gov/pubmed/?term=Stebbings%20S%5BAuthor%5D&cauthor=true&cauthor_uid=24942270), [Sherman KJ](http://www.ncbi.nlm.nih.gov/pubmed/?term=Sherman%20KJ%5BAuthor%5D&cauthor=true&cauthor_uid=24942270), [Cherkin D](http://www.ncbi.nlm.nih.gov/pubmed/?term=Cherkin%20D%5BAuthor%5D&cauthor=true&cauthor_uid=24942270), [Baxter GD](http://www.ncbi.nlm.nih.gov/pubmed/?term=Baxter%20GD%5BAuthor%5D&cauthor=true&cauthor_uid=24942270). Establishing key components of yoga interventions for musculoskeletal conditions: a Delphi survey. *BMC Complementary & Alternative Medicine* 2014; 14:196. doi: 10.1186/1472-6882-14-196

[21] Haywood KL, Griffin XL, Achten J, Costa ML. Developing a core outcome set for hip fracture trials. *The Bone and Joint Journal* 2014; 96-B:1016–23. doi 10.1302/0301-620X.96B8.33766

[22] Wylde V, MacKichan F, Bruce J, Gooberman-Hill R. Assessment of chronic post-surgical pain after knee replacement: Development of a core outcome set. *European Journal of Pain* 2015; 19: 611–20. doi: 10.1002/ejp.582

[23] Harman NL, Bruce IA, Kirkham JJ, Tierney S, Callery P et al. The importance of integration of stakeholder views in core outcome set development: otitis media with effusion in children with cleft palate. *PLoS ONE* 2015; **10**(6): e0129514. doi: 10.1371/journal.pone.0129514

[24] Dwan KM, Gamble C, Williamson PR, Kirkham JJ. Systematic Review of the Empirical Evidence of Study Publication Bias and Outcome Reporting Bias - an updated review. *PLoS ONE* 2013; 8(7): e66844. doi: 10.1371/journal.pone.0003081

[25] [Chera BS](http://www.ncbi.nlm.nih.gov/pubmed/?term=Chera%20BS%5BAuthor%5D&cauthor=true&cauthor_uid=25006189), [Eisbruch A](http://www.ncbi.nlm.nih.gov/pubmed/?term=Eisbruch%20A%5BAuthor%5D&cauthor=true&cauthor_uid=25006189), [Murphy BA](http://www.ncbi.nlm.nih.gov/pubmed/?term=Murphy%20BA%5BAuthor%5D&cauthor=true&cauthor_uid=25006189), [Ridge JA](http://www.ncbi.nlm.nih.gov/pubmed/?term=Ridge%20JA%5BAuthor%5D&cauthor=true&cauthor_uid=25006189), [Gavin P](http://www.ncbi.nlm.nih.gov/pubmed/?term=Gavin%20P%5BAuthor%5D&cauthor=true&cauthor_uid=25006189), [Reeve BB](http://www.ncbi.nlm.nih.gov/pubmed/?term=Reeve%20BB%5BAuthor%5D&cauthor=true&cauthor_uid=25006189), [Bruner DW](http://www.ncbi.nlm.nih.gov/pubmed/?term=Bruner%20DW%5BAuthor%5D&cauthor=true&cauthor_uid=25006189), [Movsas B](http://www.ncbi.nlm.nih.gov/pubmed/?term=Movsas%20B%5BAuthor%5D&cauthor=true&cauthor_uid=25006189). Recommended patient-reported core set of symptoms to measure in head and neck cancer treatment trials. *Journal of the National Cancer Institute* 2014; 106(7). pii: dju127. doi: 10.1093/jnci/dju127

[26] Miller AB, Hoogstraten B, Staquet M, Winkler A. Reporting results of cancer treatment. *Cancer* 1981; 47(1):207-14. doi: 10.1002/1097-0142

[27] Fraser JF, Hussain MS, Eskey C, Abruzzo T, Bulsara K et al. Reporting standards for endovascular chemotherapy of head, neck and CNS tumors. *Journal of Neurointerventional Surgery* 2013; 5(5): 396–9. doi:[10.1136/neurintsurg-2013-010841](http://dx.doi.org/10.1136/neurintsurg-2013-010841)

[28] MacLennan S, Bekema HJ, Williamson PR, Campbell MK, Stewart, F et al. A core outcome set for localised prostate cancer effectiveness trials: protocol for a systematic review of the literature and stakeholder involvement through interviews and a Delphi survey. *Trials* 2015; 16:76. doi: 10.1186/s13063-015-0598-0

[29] Martin NE, Massey L, Stowell C, Bangma C, Briganti, A et al. Defining a standard set of patient-centered outcomes for men with localized prostate cancer. *Eur Urol*. 2014; 67(3):460-7. doi: <http://dx.doi.org/10.1016/j.ijrobp.2014.05.1794>

[30] COMET Initiative 2016. [ONLINE] Available at: <http://www.comet-initiative.org/studies/details/286> [Accessed 11 May 2016]

[31] Fong F, Rogozinska E, Allotey J, Kempley S, Shah DK et al. Development of maternal and neonatal composite outcomes for trials evaluating management of late-onset pre-eclampsia. *Hypertens Pregnancy* 2014; 33(2): 115–31. doi: 10.3109/10641955.2013.837176

[32] Sinha I, Gallagher R, Williamson PR, Smyth RL. Development of a core outcome set for clinical trials in childhood asthma: a survey of clinicians, parents, and young people. *Trials* 2012; 13:103. doi:  [10.1186/1745-6215-13-103](http://dx.doi.org/10.1186%2F1745-6215-13-103)

[33] McNair AGK, Whistance RN, Forsythe RO, Macefied R, Rees J et al. Core outcomes for colorectal cancer surgery: a consensus study. *PLoS Medicine.* In Press

[34] van 't Hooft J, Duffy JMN, Daly M, Williamson PR, Meher S et al. [A core outcome set for evaluation of interventions to prevent preterm birth](http://ovidsp.tx.ovid.com.liverpool.idm.oclc.org/sp-3.18.0b/ovidweb.cgi?&S=APDIFPBMFJDDLDLMNCJKBDGCFOHEAA00&Link+Set=S.sh.22.23.27.31%7c9%7csl_10). Obstetrics & Gynecology 2016; 127(1):49-58. doi: 10.1097/AOG.0000000000001195

[35] Clarke M and Williamson PR. Core outcome sets and systematic reviews. *Systematic Reviews* 2016; 5:11. **doi:** 10.1186/s13643-016-0188-6

[36] McCann LJ, Kirkham JJ, Wedderburn LR, Pilkington C, Huber AM et al. Development of an internationally agreed minimal dataset for juvenile dermatomyositis (JDM) for clinical and research use. *Trials* 2015; 16:268. doi: 10.1186/s13063-015-0784-0

[37] Schmitt J, Deckert S, Alam M, Apfelbacher C, Barbaric J et al. Report from the kick-off meeting of the Cochrane Skin Group core outcome set initiative (CSG-COUSIN) . *Br. J. Dermatol.* 2016; 174(2):268. doi: 10.1111/bjd.14337

[38] Karas J, Ashkenazi S, Guarino A, Lo Vecchio A, Shamir R et al. A core outcome set for trials in acute diarrhoea. *Arch Dis Child*. 2015; 100: 359-63. doi: 10.1136/archdischild-2014-307403

[39] Howell D, Fitch M, Bakker D, Green E, Sussman J, Mayo S et al. Core domains for a person-focused outcome measurement system in cancer (PROMS-Cancer Core) for routine care: A scoping review and Canadian Delphi consensus. *Value in Health* 2013; 16:76-87. doi: 10.1016/j.jval.2012.10.017.

[40] Chiarotto A, Terwee CB, Deyo RA, Boers M, Christine Lin C-W et al. A core outcome set for clinical trials on non-specific low back pain: study protocol for the development of a core domain set. *Trials* 2014; **15**:511. **doi:** 10.1186/1745-6215-15-511

[41] Sinha IP, Smyth RL, Williamson PR. Using the Delphi technique to determine which outcomes to measure in clinical trials: recommendations for the future based on a systematic review of existing studies. *PLoS Medicine* 2011; 8(1): e1000393. doi:  [10.1371/journal.pmed.1000393](http://dx.doi.org/10.1371%2Fjournal.pmed.1000393)

[42] Harman NL, Bruce IA, Callery P, Tierney S, Sharif M et al. MOMENT-management of otitis media with effusion in cleft palate: protocol for a systematic review of the literature and identification of a core outcome set using a Delphi survey. *Trials* 2013; 14:70. doi:10.1186/1745-6215-14-70

[43] Moza A, Benstoem C, Autschbach R, Stoppe C, Goetzenich A. A core outcome set for all types of cardiac surgery effectiveness trials: a study protocol for an international eDelphi survey to achieve consensus on what to measure and the subsequent selection of measurement instruments *Trials* 2015; 16:545. doi: 10.1186/s13063-015-1072-8

[44] Clarke M, Williamson PR. Core outcome sets and trial registries. *Trials* 2015; 16:216. **doi:** 10.1186/s13063-015-0738-6

[45] Blazeby JM, Macefield R, Blencowe NS, Jacobs M, McNair AG et al. Core information set for oesophageal cancer surgery. British Journal of Surgery 2015; 102(8): 936-43. doi: 10.1002/bjs.9840

[46] Potter S, Holcombe C, Ward JA, Blazeby JM. Development of a core outcome set for reconstructive breast surgery The BRAVO (Breast Reconstruction and Valid Outcomes) Study. *British Journal of Surgery* 2015; 102 (11): 1360-71. doi [10.1002/bjs.9883](http://dx.doi.org/10.1002/bjs.9883)

[47] Devane D, Begley CM, Clarke M, Horey D, O’Boyle C. Evaluating maternity care –

a core set of outcome measures. *Birth* 2007; 34 (2): 164-72

[48] Smaïl-Faugeron V, Chabouis H, Durieux P, Attal JP, Muller-Bolla M et al. Development of a core set of outcomes for randomized controlled trials with multiple outcomes – example of pulp treatments of primary teeth for extensive decay in children. *PLoS One* 2013; 8(1):e51908. doi:  [10.1371/journal.pone.0051908](http://dx.doi.org/10.1371%2Fjournal.pone.0051908)

[49] Chiarotto A, Deyo RA, Terwee CB, Boers M, Buchbinder R et al (2015). Core outcome domains for clinical trials in non-specific low back pain. *Eur Spine J*; 24(6): 1127-42. doi 10.1007/s00586-015-3892-3

[50] Rider LG, Giannini EH, Brunner HI, Ruperto N, James-Newton L et al. International consensus on preliminary definitions of improvement in adult and juvenile myositis. *Arthritis Rheum* 2004; 50(7):2281-90. doi: 10.1002/art.20349

[51] Kirchhof P, Auricchio A, Bax J, Crijns H, Camm J et al. Outcome parameters for trials in atrial fibrillation: executive summary. *European Heart Journal* 2007; 28(22):2803-2817. doi: <http://dx.doi.org/10.1093/eurheartj/ehm358>

[52] Ruperto N, Ravelli A, Murray KJ, Lovell DJ, Andersson-Gare B et al. Preliminary core sets of measures for disease activity and damage assessment in juvenile systemic lupus erythematosus and juvenile dermatomyositis. *Rheumatology* 2003; 42(12):1452-9. doi: 10.1093/rheumatology/keg403

[53] [Chen RC](http://www.ncbi.nlm.nih.gov/pubmed/?term=Chen%20RC%5BAuthor%5D&cauthor=true&cauthor_uid=25006192), [Chang P](http://www.ncbi.nlm.nih.gov/pubmed/?term=Chang%20P%5BAuthor%5D&cauthor=true&cauthor_uid=25006192), [Vetter RJ](http://www.ncbi.nlm.nih.gov/pubmed/?term=Vetter%20RJ%5BAuthor%5D&cauthor=true&cauthor_uid=25006192), [Lukka H](http://www.ncbi.nlm.nih.gov/pubmed/?term=Lukka%20H%5BAuthor%5D&cauthor=true&cauthor_uid=25006192), [Stokes WA](http://www.ncbi.nlm.nih.gov/pubmed/?term=Stokes%20WA%5BAuthor%5D&cauthor=true&cauthor_uid=25006192) et al. Recommended patient-reported core set of symptoms to measure in prostate cancer treatment trials. *Journal of the National Cancer Institute* 2014; 106(7). pii: dju132. doi: 10.1093/jnci/dju132

[54] [Saketkoo LA](http://www.ncbi.nlm.nih.gov/pubmed/?term=Saketkoo%20LA%5BAuthor%5D&cauthor=true&cauthor_uid=24368713), [Mittoo S](http://www.ncbi.nlm.nih.gov/pubmed/?term=Mittoo%20S%5BAuthor%5D&cauthor=true&cauthor_uid=24368713), [Huscher D](http://www.ncbi.nlm.nih.gov/pubmed/?term=Huscher%20D%5BAuthor%5D&cauthor=true&cauthor_uid=24368713), [Khanna D](http://www.ncbi.nlm.nih.gov/pubmed/?term=Khanna%20D%5BAuthor%5D&cauthor=true&cauthor_uid=24368713), [Dellaripa PF](http://www.ncbi.nlm.nih.gov/pubmed/?term=Dellaripa%20PF%5BAuthor%5D&cauthor=true&cauthor_uid=24368713) et al. Connective tissue disease related interstitial lung diseases and idiopathic pulmonary fibrosis: provisional core sets of domains and instruments for use in clinical trials. *Thorax* 2014; 69(5): 428-436. doi: 10.1136/thoraxjnl-2013-204202

[55] Bennett WL, Robinson KA, Saldanha IJ, Wilson LM, Nicholson WK. High priority research needs for gestational diabetes mellitus. *Journal of Women’s Health* 2012; 21(9):925-932. doi:  [10.1089/jwh.2011.3270](http://dx.doi.org/10.1089%2Fjwh.2011.3270)

[56] Schmitt J, Langan S, Stamm T, Williams HC. Core outcome set domains for controlled trials and clinical recordkeeping in eczema: international multiperspective Delphi consensus process. *Journal of Investigative Dermatology* 2011; 131(3):623-630. doi: <http://dx.doi.org/10.1038/jid.2010.303>

[57] Vargus-Adams JN, Martin LK. Measuring What Matters in Cerebral Palsy: A Breadth of Important Domains and Outcome Measures. *Archives of Physical Medicine and Rehabilitation* 2009*;* 90:2089-2095. doi:<http://dx.doi.org/10.1016/j.apmr.2009.06.018>

[58] Kloppenburg M, Bøyesen P, Smeets W, Haugen IK, Liu R et al. Report from the OMERACT hand osteoarthritis special interest group: advances and future research priorities. *J Rheumatol* 2014; 41(4): 810-8. doi: 10.3899/jrheum.131253

[59] Mease PJ, Clauw DJ, Arnold LM, Goldenberg DL, Witter J et al. Fibromyalgia syndrome. See comment in PubMed Commons below[*J Rheumatol.*](http://www.ncbi.nlm.nih.gov/pubmed/16265715) 2005; 32(11):2270-7

[60] Diamond IR, Grant RC, Feldman BM, Pencharz PB, Ling SC, Moore AM, Wales PW. Defining consensus: a systematic review recommends methodologic criteria for reporting of Delphi studies. Journal of Clinical Epidemiology 2014; 67(4):401-9. [doi:10.1016/j.jclinepi.2013.12.002](http://dx.doi.org/10.1016/j.jclinepi.2013.12.002)

[61] Bruce I, Harman N, Williamson P, Tierney S, Callery P, Mohiuddin S, Payne K, Fenwick E, Kirkham J, O'Brien K. The management of Otitis Media with Effusion in children with cleft palate (mOMEnt): a feasibility study and economic evaluation. Health Technol Assess. 2015;19(68). doi: 10.3310/hta19680

[62] Boers M, Tugwell P, Felson DT, van Riel PLCM, Kirwan JR et al. World Health Organization and International League of Associations for Rheumatology core endpoints for symptom modifying anti rheumatic drugs in rheumatoid arthritis clinical trials. *J Rheumatol* 1994*;* **(**suppl 41) 21:86-9)

[63] Sibley KM, Howe T, Lamb SE, Lord SR, Maki BE et al. Recommendations for a core outcome set for measuring standing balance in adult populations: a consensus-based approach. *PLoS ONE* 2015; 10(3). doi: 10.1371/journal.pone.0120568

[64] [Donovan KA](http://www.ncbi.nlm.nih.gov/pubmed/?term=Donovan%20KA%5BAuthor%5D&cauthor=true&cauthor_uid=25006190), [Donovan HS](http://www.ncbi.nlm.nih.gov/pubmed/?term=Donovan%20HS%5BAuthor%5D&cauthor=true&cauthor_uid=25006190), [Cella D](http://www.ncbi.nlm.nih.gov/pubmed/?term=Cella%20D%5BAuthor%5D&cauthor=true&cauthor_uid=25006190), [Gaines ME](http://www.ncbi.nlm.nih.gov/pubmed/?term=Gaines%20ME%5BAuthor%5D&cauthor=true&cauthor_uid=25006190), [Penson RT](http://www.ncbi.nlm.nih.gov/pubmed/?term=Penson%20RT%5BAuthor%5D&cauthor=true&cauthor_uid=25006190) et al. Recommended patient-reported core set of symptoms and quality-of-life domains to measure in ovarian cancer treatment trials. [*J Natl Cancer Inst*.](http://www.ncbi.nlm.nih.gov/pubmed/25006190) 2014; 106(7): dju128. doi: 10.1093/jnci/dju128

[65] Plint AC, Moher D, Morrison A, Schulz K, Altman DG et al. Does the CONSORT checklist improve the quality of reports of randomised controlled trials? A systematic review. See comment in PubMed Commons below[*Med J Aust.*](http://www.ncbi.nlm.nih.gov/pubmed/16948622) 2006;185(5):263-7.

[66] Turner L, Moher D, Shamseer L, Weeks L, Peters J et al. The influence of CONSORT on the quality of reporting of randomized controlled trials: an updated review. Trials 2011, 12 (suppl 1). doi:  [10.1186/1745-6215-12-S1-A47](http://dx.doi.org/10.1186%2F1745-6215-12-S1-A47)

[67] Turner L, Shamseer L, Altman DG, Weeks L, Peters J, Kober T, Dias S, Schulz KF, Plint AC, Moher D. Consolidated standards of reporting trials (CONSORT) and the completeness of reporting of randomised controlled trials (RCTs) published in medical journals. *Cochrane Database of Systematic Reviews* 2012, Issue 11. Art. No.: MR000030. doi: 10.1002/14651858.MR000030.pub2.

[68] Moher D, Shamseer L, Clarke M, Ghersi M, Petticrew et al. Preferred reporting items for systematic review and meta-analysis protocols (PRISMA-P) 2015 statement. *Systematic Reviews* 2015; 4:1 **doi:** 10.1186/2046-4053-4-1

[69] Moher D, Schulz KF, Simera I, Altman DG. Guidance for developers of health research reporting guidelines. *PloS Medicine* 2010; 7(2): e1000217. doi:10.1371/journal.pmed.1000217
